# Systematic elucidation of neuron-astrocyte interaction in models of amyotrophic lateral sclerosis using multi-modal integrated bioinformatics workflow

**DOI:** 10.1038/s41467-020-19177-y

**Published:** 2020-11-04

**Authors:** Vartika Mishra, Diane B. Re, Virginia Le Verche, Mariano J. Alvarez, Alessandro Vasciaveo, Arnaud Jacquier, Paschalis-Tomas Doulias, Todd M. Greco, Monica Nizzardo, Dimitra Papadimitriou, Tetsuya Nagata, Paola Rinchetti, Eduardo J. Perez-Torres, Kristin A. Politi, Burcin Ikiz, Kevin Clare, Manuel E. Than, Stefania Corti, Harry Ischiropoulos, Francesco Lotti, Andrea Califano, Serge Przedborski

**Affiliations:** 1 Departments of Pathology and Cell Biology, Columbia University, New York, NY 10032 USA; 2 Center for Motor Neuron Biology and Diseases, Columbia University, New York, NY 10032 USA; 3 Department of Environmental Health Sciences, Columbia University, New York, NY 10032 USA; 4 Department of Systems Biology, Columbia University, New York, NY 10032 USA; 5 DarwinHealth Inc., New York, NY 10032 USA; 6 Herbert Irving Comprehensive Cancer Center, Columbia University, New York, NY USA; 7 Department of Pediatrics, Children’s Hospital of Philadelphia Research Institute and the University of Pennsylvania, Philadelphia, PA 19104 USA; 8 Dino Ferrari Center, Department of Pathophysiology and Transplantation, University of Milan, Neurology Unit, IRCCS Foundation Ca’ Granda Ospedale Maggiore Policlinico, Milan, 20122 Italy; 9 Protein Crystallography Group, Leibniz Institute on Aging – Fritz Lipmann Institute (FLI), Beutenbergstr. 11, 07745 Jena, Germany; 10 J.P. Sulzberger Columbia Genome Center, Columbia University, New York, NY USA; 11 Department of Biomedical Informatics, Columbia University, New York, NY USA; 12 Department of Biochemistry and Molecular Biophysics, Columbia University, New York, NY USA; 13 Departments of Neurology and Neuroscience, Columbia University, New York, NY 10032 USA; 14 Present Address: Spark Therapeutics, 3737 Market Street, Philadelphia, PA 19104 USA; 15 Present Address: Center for Gene Therapy, City of Hope, 1500 E. Duarte Road, Duarte, CA 91010 USA; 16 Present Address: Institut NeuroMyoGène, CNRS UMR 5310 - INSERM U1217 - Université de Lyon - Université Claude Bernard Lyon 1, Lyon, France; 17 Present Address: Department of Molecular Biology, Princeton University, Princeton, USA; 18 Present Address: Henry Dunant Hospital, BRFAA, Athens, Greece; 19 Present Address: Department of Neurology and Neurological Science, Tokyo Medical and Dental University, Tokyo, Japan; 20 Present Address: New York Medical College, Valhalla, NY 10595 USA

**Keywords:** Diseases of the nervous system, Amyotrophic lateral sclerosis, Molecular neuroscience, Systems analysis

## Abstract

Cell-to-cell communications are critical determinants of pathophysiological phenotypes, but methodologies for their systematic elucidation are lacking. Herein, we propose an approach for the Systematic Elucidation and Assessment of Regulatory Cell-to-cell Interaction Networks (SEARCHIN) to identify ligand-mediated interactions between distinct cellular compartments. To test this approach, we selected a model of amyotrophic lateral sclerosis (ALS), in which astrocytes expressing mutant superoxide dismutase-1 (mutSOD1) kill wild-type motor neurons (MNs) by an unknown mechanism. Our integrative analysis that combines proteomics and regulatory network analysis infers the interaction between astrocyte-released amyloid precursor protein (APP) and death receptor-6 (DR6) on MNs as the top predicted ligand-receptor pair. The inferred deleterious role of APP and DR6 is confirmed in vitro in models of ALS. Moreover, the DR6 knockdown in MNs of transgenic mutSOD1 mice attenuates the ALS-like phenotype. Our results support the usefulness of integrative, systems biology approach to gain insights into complex neurobiological disease processes as in ALS and posit that the proposed methodology is not restricted to this biological context and could be used in a variety of other non-cell-autonomous communication mechanisms.

## Abstract (web summary)

Neuron-astrocyte communication plays a key role in pathophysiology, however systematic approaches to unveil it are limited. Here, the authors propose SEARCHIN, a multi-modal integrated workflow, as a tool to identify cross-compartment ligand-receptor interactions, applied to ALS models.

## Introduction

The rationale for the elucidation of mechanisms that mediate cell-to-cell communication processes is that ligands released by one cell type may induce specific changes in gene-product activity in one or more additional cell types, ultimately resulting in an observable molecular phenotype. The latter can range from the activation of specific immune response pathways to the aberrant reprogramming of cell state and even cell death. Classical hypothesis-driven mechanistic elucidation of these intercellular signaling pathways requires complex, time-consuming, and laborious work to gradually restrict the number of molecular players that may mediate the cell-to-cell interaction of interest. As a result, high-throughput methodologies for the systematic prioritization of process-specific ligand–receptor interactions are critically needed, yet, still largely elusive.

By combining recent results on modeling transcriptional, signal transduction and other context-specific molecular interaction networks with proteomics, we propose an approach for the Systematic Elucidation and Assessment of Regulatory Cell-to-cell Interaction Networks (SEARCHIN) to prioritize cross-compartment ligand–receptor interactions that may mediate specific cellular phenotypes. Specifically, SEARCHIN implements a workflow that integrates several algorithms, such as ARACNe^1^, VIPER^2^, MINDy^3^, and PrePPI^4^, that were not originally designed to study the cell-cell communication process. As a proof-of-concept for its utilization, we elect to study amyotrophic lateral sclerosis (ALS), a common fatal, paralytic disorder that provides an especially relevant context. Indeed, although ALS is characterized by preferential death of motor neurons (MNs)^5^, the contribution of aberrant cell-to-cell interactions involving non-neuronal cells such as glia to the neurodegenerative process is increasingly acknowledged^6^. Specifically, several investigators have exploited the fact that mutations in superoxide dismutase-1 (mutSOD1) gene not only cause a familial form of ALS^7^, but also provide an ideal molecular tool to study non-cell autonomous mechanisms given their cell-ubiquitous expression and toxic gain-of-function properties. In keeping with this, we and others have reported that astrocytes expressing mutSOD1 selectively kill wild-type (WT) mouse primary spinal MNs and embryonic stem cell-derived MNs (ES-MNs)^8–20^. This spontaneous neurodegenerative phenotype was observed either when MNs were cultured in the presence of mutSOD1-expressing astrocytes or when they were exposed to medium conditioned by mutant astrocytes^8,9,20^. Similar to these findings from mouse mutSOD1-expressing astrocytes, we showed that astrocytes derived from postmortem CNS samples from human sporadic ALS (sALS) patients were also associated with a loss of human ES-MNs^9^. Furthermore, it has been reported that mutSOD1-expressing glial-restricted precursor cells grafted onto spinal cords (SCs) of WT rats were also associated with MN loss in living animals^21^ and that selective reduction of mutSOD1 levels in astrocytes prolonged survival in transgenic (Tg) SOD1^G37R^ mice^22^. Taken together, these observations suggest that astrocyte-mediated MN degeneration is a general phenomenon in ALS and is not restricted to in vitro systems, mouse cells, or mutSOD1-linked ALS.

Since our initial observation^20^, we gained significant insights into the MN-intrinsic molecular cascade that drives neurodegeneration in vitro, both in murine mutSOD1 and in human sALS cells^8,9^. For instance, our published data support NF-κB1 as a likely apical master regulator (MR), whose translocation to the nucleus of cultured MNs mediates mutSOD1 astrocyte-induced neurodegeneration^8^. As a result, it is reasonable to assume the existence of a ligand released by mutSOD1-expressing astrocytes, capable of activating a receptor-mediated signal in MNs, ultimately leading to NF-κB1 activation. However, the precise mechanisms promoting astrocyte-induced demise of neighboring MNs in ALS remain elusive.

In this paper, we developed the SEARCHIN pipeline to integrate information on proteins enriched in medium conditioned by mutSOD1 astrocytes with regulatory network-based analysis of their cognate receptor-mediated NF-κB1 activation in MNs, a previously identified MR of MN demise. The analysis identified amyloid precursor protein (APP) as the most likely toxic factor released by the astrocytes. In addition, SEARCHIN identified the Tumor Necrosis Factor Receptor Superfamily member 21 (TNFRSF21)—also known as death receptor-6 (DR6)—as the APP cognate receptor most likely to be responsible for transducing mutSOD1 astrocyte-induced death in MNs. We confirmed these predictions experimentally by showing that MN toxicity depends on the expression of APP in astrocytes and DR6 in MNs, in both mouse mutSOD1 and human sALS astrocytes. In vivo, RNA interference (RNAi)-mediated knockdown of DR6 in MNs-attenuated neurodegeneration.

In light of these data and of our previous studies^8,9^, we propose a pathogenic model of neurodegeneration in ALS in which astrocyte-specific release of a soluble fragment of APP, and possibly of other ligands prioritized by our analysis, activates DR6 at the surface of MNs, thus triggering a death signal that culminates in the demise of spinal MNs via NF-κB1-dependent pathway. In addition, these data support the use of SEARCHIN as a pipeline to systematically prioritize candidate ligand–receptor interactions that may mediate a variety of pathophysiological processes, across diverse cellular compartments.

## Results

### Mouse mutSOD1 astrocytes mediate MN death by a toxic proteinaceous factor(s)

Based on our previous data, we have observed significant reduction in MN number after exposure to either mouse mutSOD1 and human sALS astrocytes or their corresponding astrocyte-conditioned media (ACMs)^8,9,20^, resulting in a ~50% reduction of healthy MNs by day 7 in vitro (DIV 7). To determine whether the deleterious effect of ALS astrocytes is mediated by either a progressive loss-of-supportive function or a buildup-of-toxic factor(s), three sets of experiments were performed.

**Fig. 1:**
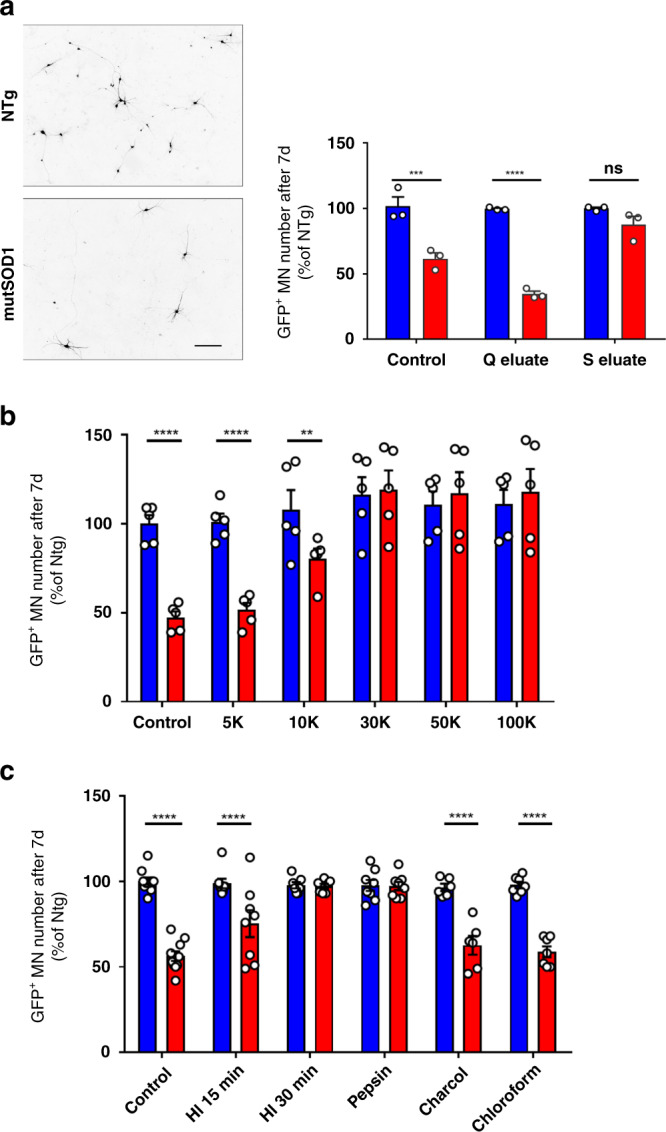
Characterization of the deleterious effects of ACM from Tg mutSOD1 mice. **a** Representative images of MN cultures exposed to NTg (blue) and mutSOD1 (red) ACM stained with the neuronal marker SMI-32. Scale bar: 50 μm. ACM from NTg (*n* = 3) or mutSOD1 (*n* = 3) astrocytes were passed through anion exchange Q column or cation exchange S column. Elutes were collected and then applied to mouse primary MNs. GFP+MNs were counted manually using epifluorescent microscope. Data are means ± SEM of independent experiments (*n*) analyzed by two-way ANOVA (Interaction F_(2,12)_ = 17.61; *P* = 0.0003) followed by Sidak post hoc test: ***P = 0.0001 Control NTg vs mutSOD1 (CI 22.61–57.39%; *d* = 4.73); ****P ≤ 0.0001 Q eluate NTg vs mutSOD1 (CI: 47.61–82.39%; *d* = 28.47). **b** ACM from NTg (blue; *n* = 5) or mutSOD1 (red; *n* = 5) were passed through different molecular weight cutoff filters. The retentates were resuspended in fresh media and then applied to mouse primary MN culture and counted manually. Data are means ± SEM of independent experiments (*n*) analyzed by two-way ANOVA (Interaction F_(5,24)_ = 19.14; *P* < 0.0001) followed by Sidak post hoc test: ****P ≤ 0.0001 Control NTg vs mutSOD1 (CI: 34.26–70.94%; *d* = 6.35) and 5 K NTg vs mutSOD1 (CI: 31.06–67.74%; *d* = 5.76); **P = 0.0015 10 K NTg vs mutSOD1 (CI: 9.06–45.74%; *d* = 1.55). **c** To examine molecular property of the deleterious effect in MNs, ACM from NTg (blue) or mutSOD1 (red) mice was either untreated (*n* = 10), heat-inactivated using water bath (*n* = 8 HI 15 min and *n* = 7 HI 30 min), treated with pepsin (*n* = 8), charcoal (*n* = 6) or chloroform (*n* = 7) and then applied to mouse primary MNs and counted manually. Data are mean ± SEM of independent experiments (*n*) analyzed by two-way ANOVA (Interaction F_(5,40)_ = 21.22; *P* < 0.0001) followed by Sidak post hoc test: ****P ≤ 0.0001 Control NTg vs mutSOD1 (CI: 33.61–53.99%; *d* = 5.86), HI 15 min NTg vs mutSOD1 (CI: 11.98–34.77%; *d* = 1.63), charcoal NTg vs mutSOD1 (CI: 20.68–46.99%; *d* = 3.24), and chloroform NTg vs mutSOD1 (CI: 26.82–51.18%; *d* = 6.10). In Fig. 1, all primary spinal MN cultures were from Tg HB9::EGFP mice. See also Supplementary Fig. 1. Source data provided as source data file.

First, both mouse mutSOD1 and non-transgenic (NTg) control ACMs were subjected to ion-exchange liquid chromatography. We found that the Q-column (i.e., positively charged quaternary amines-coated column) eluates from mutSOD1 ACMs were associated with greater MN loss at 7 DIV, compared with unfiltered mutSOD1 ACMs after 5 days incubation (Fig. 1a). Meanwhile, Q column eluates from NTg ACMs were not associated with any significant reduction of MN numbers, compared with their unfiltered counterparts (Fig. 1a). In contrast, there was no significant difference in MN numbers at 7 DIV between cultures incubated for 5 days with the S column (i.e., negatively charged sulfate derivatives-coated column) eluates from mutSOD1 or NTg ACMs (Fig. 1a). These results suggest the presence of a negatively charged toxic factor in mouse mutSOD1 ACM that can be enriched using Q column ion-exchange liquid chromatography.

Second, mouse mutSOD1 and NTg ACMs were applied to Amicon® centrifugal filter units with five different nominal molecular weight cut offs ranging from 5 to 100-kDa. After centrifugation, retentates from both the mutSOD1 and NTg ACMs were resuspended in fresh medium to restore their original volumes and then applied to WT spinal MN cultures^20^. Similar to the untreated control ACMs, we observed significantly fewer MNs at 7 DIV after a 5-day exposure to resuspended retentates from the 5- and 10-kDa cutoff filtrations of mutSOD1 ACMs vs. NTg ACMs (Fig. 1b). In contrast, there was no significant difference in MN numbers at 7 DIV after a 5-day exposure to resuspended retentates from the 30-, 50-, and 100-kDa cutoff filtrations of mutSOD1 ACMs vs. NTg ACMs (Fig. 1b); similar results were obtained with molecular weight cutoff dialysis cassettes.

Third, we submitted the mouse ACMs to a series of treatments such as denaturation, proteases, charcoal, or chloroform extraction (Fig. 1c). These different treatments revealed that the effect of mutSOD1 ACMs on MN survival was thermolabile, protease sensitive and not affected by the charcoal or chloroform extraction (Fig. 1c). Altogether, these findings indicate that, rather than failing to exert beneficial effects on MNs, mouse mutSOD1 ACMs exert a toxic activity mediated by protein(s) or fragment(s) of ≤30-kDa.

SOD1 protein, with a molecular mass of ~16 kDa is an obvious potential target and was tested using two different approaches. First, we sought to immunodeplete SOD1 from ACMs of both genotypes with an anti-SOD1 antibody, but this did not abrogate mutSOD1 ACM-induced MN toxicity (Supplementary Fig. 1a). In fact, a close inspection of the data, revealed that SOD1 immunodepletion in mutSOD1 ACM seemed to be associated with an enhanced MN toxicity, suggesting that SOD1 in mouse ACM might mitigate the deleterious effects of ALS astrocytes rather than causing it. We then supplemented NTg ACM with SOD1 recombinants (Supplementary Fig. 1b) and this failed to cause MN death, despite the presence of copious amount of chromogranin, which upon interacting with mutSOD1 was reported to cause MN death^23^. Thus, these results allowed us to discount SOD1 protein as the likely toxic factor in our in vitro mouse mutSOD1 model of ALS.

### Proteomic profile of ACM from mutSOD1 does not reflect an A1 phenotype

To identify potential toxic factors released by mouse mutSOD1 astrocytes, we started by performing differential liquid chromatography with tandem mass spectrometry (LC-MS/MS)-based proteomics on Q-column-eluted ACMs of NTg vs. mutSOD1 astrocytes, as previously described^24^, except that protein identification was performed with MaxQuant using UniProt database. Analysis of three biological replicates revealed a list of 86 proteins in the Q-column-eluted ACMs (Supplementary Table 1). Of these 86 proteins, 10 showed ≥1.5-fold increase in mutSOD1 ACM compared to NTg ACM and 55 were only present in the mutSOD1 fraction; the remaining 21 were either unchanged or only present/increased in NTg ACM (Supplementary Table 1).

Next, we asked whether mouse mutSOD1 ACM reflects a profile of reactive astrocytes and more specifically A1, a particular astrocyte neurotoxic phenotype induced by lipopolysaccharide (LPS) treatment^25,26^. Among the 13 pan-reactive astrocyte genes listed in Liddelow, et al.^26^, products for only two (LCN2 and GFAP) were found in the ACMs and GFAP was only found in the mutSOD1 ACM. Furthermore, none of the products of the neurotoxic A1 genes listed in Liddelow, et al.^26^ were differentially expressed between mutSOD1 and NTg ACMs. These results are consistent with our previous conclusions that the cultured mouse mutSOD1 and NTg astrocytes used herein are reactive but not differentially so^8,9,20^ and that likely mutSOD1 astrocytes and ACM do not exert toxicity via an LPS-induced A1 mechanism.

### Integrative systems biology approach to infer toxic ligand–receptor interactions

**Fig. 2:**
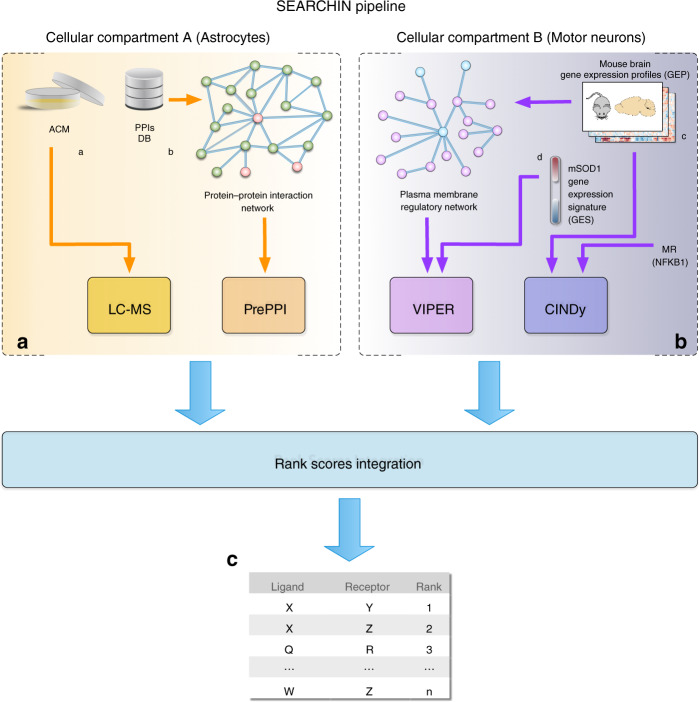
Conceptual workflow of the SEARCHIN pipeline. **a** Cellular compartment A (ACM from astrocytes) was analyzed using inputs: (I) list of candidate ligands enriched in the Astrocyte Condition Media (ACM) and (II) Protein-protein interactions (PPIs) interactome, both derived as explained in the Methods Section. **b** Cellular compartment B (MNs) was compiled using inputs: (III) set of gene expression profiles (GEP) from tissue type relative to cellular compartment B, for both, generation of a transcriptional regulatory interactome and modulator analysis and (IV) gene expression signature (GES) generated as differential between presence and absence of ligand signals. **c** The SEARCHIN pipeline produces a list of inferred ligand–receptor interactions prioritized based on the evidences assessed in **a**, **b**. Please see Methods section for detailed description. See also Table 1 and Supplementary Tables 1–4. Source data provided as source data file.

To identify potential toxic factors released by mouse mutSOD1 astrocytes, we developed an unbiased workflow (SEARCHIN) for the de novo prioritization of candidate ligand–receptor interactions that may mediate a given cell-to-cell communication process, for follow-up validation. Specifically, SEARCHIN integrates four analyses, providing independent evidence supporting phenotypically relevant ligand–receptor interactions (Fig. 2).

First, candidate ligands released by one cellular compartment (i.e., astrocytes, in this case) are identified by LC-MS/MS. Second, the predicting protein-protein interaction (Pre-PPI) algorithm^4^ is used to assign a probability to all of their potential ligand–receptor interactions. PrePPI uses protein/peptide structure information, from both established experimental and homology-based models, as well as context-specific gene expression data, to predict candidate ligand-protein interactions. Third, the VIPER algorithm^2^ is used to assess differential activity of cell-surface and nuclear receptor proteins in the target cellular compartment (i.e., MNs, in this case), based on differential gene expression signatures in the presence and absence of the ligand. VIPER uses the differential gene expression of the most direct regulatory targets affected by a protein to measure its differential activity, akin to a multiplexed, cell-context-specific gene reporter assay. Fourth, after the MR proteins controlling the phenotype of interest in the MN compartment are identified by VIPER, the CINDy algorithm^3^ is used to prioritize cell-surface and nuclear receptors that may modulate their activity through signal transduction pathways. In this case, the VIPER analysis had already been performed, leading to identification of NF-κB1 as the key MR protein^8^. Finally, the individual probabilities from steps 2 to 4 are integrated to achieve a final prioritization of ligand–receptor interactions (Fig. 2).

With the information about the Q-column-eluted mutSOD1 ACM proteome in hand, we sought to identify the receptor(s) that most likely transduce the MN death signal. For this subsequent step, we used the PrePPI database^4^ to build a protein–protein interaction (PPI) network involving the candidate ligands, thus obtaining a probability-ranked list of putative ligand–receptor interactions (Supplementary Table 2). Next, we reasoned that if the toxic activity was mediated by one or more surface receptors, we should be able to assess their differential VIPER-inferred activity, in the presence of mutSOD1 vs. NTg ACM, thus allowing further prioritization of candidate ligand–receptor interactions for experimental validation.

VIPER analysis requires a regulatory model, representing the context-specific (in this case MN-specific) interactions between each regulator protein and its downstream transcriptional targets (i.e., its regulon). To assemble such a model for all proteins annotated as surface receptors (i.e., the receptors’ interactome), we relied on a mouse brain-specific regulatory model inferred by the ARACNe algorithm (see Methods). We then used the VIPER algorithm to compute the enrichment of activated and repressed targets of each receptor in genes that were differentially expressed in previously published gene expression signatures from purified ES-derived MNs, exposed to either mutSOD1 or NTg ACM^8^. The analysis identified several receptor proteins, with known signal transduction properties, that were significantly activated in response to mutSOD1 ACM exposure (Supplementary Table 3). Moreover, we posited that if the receptor’s differential activity was responsible for inducing the MN death phenotype, then the receptor should trigger a signaling cascade leading to activation of the previously identified MRs of mSOD1-mediated MN toxicity, and, in particular of NF-κB1^8^. The focus on NF-κB1 was driven by the fact that it was previously validated as a key driver of mutSOD1 astrocyte-induced MN degeneration^8^, whereas other inferred MRs were less effective in modulating the MN death phenotype or were shown to be direct NF-κB1 targets^8^. Thus, MR analysis was pivotal in identifying candidate protein receptors significantly by focusing on NF-κB1. As a result, experimental validation of candidate MRs is a valuable step to select MR proteins for SEARCHIN analysis, even though the analysis can also be performed with multiple candidate MRs. The CINDy algorithm was designed to identify upstream candidate modulators of transcription factor activity, thus providing a methodology to measure the potential ability of any receptor to modulate NF-κB1 activity (Supplementary Table 4). Of note, although NF-κB1 was identified as a key MR in MN death in our in vitro models of ALS^8^, as we used purified MN cultures, we cannot exclude that NF-κB1 is also activated in other neurons and even other SC cells that express DR6.

Finally, we elected to use the evidence integration Robust Rank Aggregation method^27,28^ to integrate the ranked lists produced by each evidence source (i.e., PrePPI, VIPER, and CINDy), based on order statistics (Fig. 2). This is especially important given that use of different null models in different algorithms may significantly bias *p* value estimates, making them non-comparable. For instance, CINDy *p* values tend to be much smaller than VIPER *p* values and would thus dominate the output of any *p* value-based evidence integration method. Although more sophisticated methodologies may further improve results, we strived to implement the simplest possible solution for this “proof-of-concept” implementation, leaving further refinements to future studies.

Results of our integrative analysis for proteins that were enriched or only present in the mouse mutSOD1 ACM revealed three putative ligand–receptor interactions with a *p* value ≤0.01 (Table 1). Next, we mined the scientific literature and primary PPI databases^29–31^ and found that of these three putative protein–protein interaction, only the APP–DR6 interaction was validated and confirmed by functional assays, albeit not in an ALS-relevant context^32–34^ and was thus selected for further investigations. Therefore, a value of the SEARCHIN pipeline is the ability to reduce a potential list of tens of thousands of potential interactions between all over-abundant ligands produced by one compartment and all receptors in the other compartment to a ranked list, from which candidates for experimental validation can be effectively selected, based either on additional knowledge (e.g., prior validation of the ligand–receptor interaction) or simply starting from the most statistically significant one.

**Table 1:** SEARCHIN-based prioritization of candidate ligand–receptor interactions.

No.	Ligand–repector predicted interaction	Score
1	Nme1-Ptprn	0.005189
2	Cdh2-Ryk	0.006011
3	App-Tnfrsf21	0.006891
4	Lgals1-Spn	0.016729
5	Crk-Tgfbr2	0.016729
6	App-Adgrl1	0.016729
7	App-Glrb	0.018234
8	Ccn2-Itgb5	0.018798
9	Cnbp-Rap1a	0.021792
10	B2m-Osmr	0.021792
11	Cxcl5-Cx3cl1	0.023618
12	Ccl3-Cx3cl1	0.025303
13	Igf1-Itsn1	0.027218
14	Hmox1-Itgb2	0.030239
15	Hp-Itgb2	0.031028
16	Mapre1-Rab35	0.033395
17	B2m-Tgfbr2	0.033395
18	App-Ryk	0.033395
19	Crk-Rras	0.036937
20	Cdh2-Glrb	0.038753
21	Cst3-Hbegf	0.042941
22	Crk-Ephb3	0.043552
23	Cdh2-Lingo1	0.047665
24	Crk-Arhgap17	0.049999
25	Sod1-Tgfbr2	0.049999
26	Crk-Ryk	0.049999
27	Cdh2-Itgb1	0.050914
28	Crk-Itsn1	0.050914
29	App-Tgfbr2	0.054627
30	App-Lingo1	0.057122
31	Cdh2-Grik2	0.066541
32	Mapre1-Ryk	0.066541
33	Ccn2-Tgfbr2	0.066541
34	Cdh2-Itpr3	0.068034
35	Igfbp5-Rras	0.077874
36	App-Homer3	0.083021
37	Ltbp2-Tgfbr2	0.083021
38	Cfl1-Mark2	0.0839
39	Lgals3-P2ry12	0.087964
40	Ccn2-Itgb1	0.090229
41	Igf1-Il31ra	0.09107
42	B2m-Bcl10	0.09186
43	B2m-Cdh13	0.09186
44	B2m-Cd48	0.094226
45	Sod1-Sri	0.099439
46	Ccn2-Hbegf	0.100684
47	App-Mast1	0.103987
48	Hmox1-Rab3d	0.107428
49	Cst3-Furin	0.111111
50	Pdia3-Atp6ap2	0.115796
51	Gaa-P2rx4	0.121222
52	App-Rgs14	0.122663
53	B2m-Itgb1	0.130793
54	App-Homer2	0.132091
55	B2m-Treml1	0.132091
56	App-Gria1	0.132091
57	Crk-Pdgfra	0.135862
58	Lgals1-Cx3cl1	0.135862
59	Lgals1-Rhoc	0.139274
60	App-Akt1	0.148324
61	App-Grik3	0.148324
62	App-Gabrb1	0.148324
63	Crk-Kitl	0.152671
64	Cfl1-Kitl	0.154985
65	Igf1-Kitl	0.157321
66	Cfl1-Rras	0.159681
67	Crk-Rap1a	0.164471
68	App-Diras1	0.164496
69	App-Il31ra	0.164496
70	Hnrnpk-Itgb1	0.169356
71	B2m-Il31ra	0.180607
72	Crk-Diras1	0.180607
73	Cdh2-Itgb2	0.181989
74	Lgals1-Atp6ap2	0.184178
75	B2m-Hbegf	0.187214
76	App-Arhgap17	0.195236
77	Cfl1-Iqgap1	0.196657
78	App-Cap2	0.196657
79	Crk-Pdgfrb	0.197253
80	Igf1-Itk	0.200709
81	Igf1-Rhoh	0.200709
82	Crk-Cyth3	0.200709
83	Hnrnpk-Itk	0.203484
84	B2m-Tnfsf13b	0.206185
85	B2m-Tnfsf12	0.20911
86	Cdh2-Itsn1	0.20911
87	App-Itgb1	0.212645
88	App-Gria3	0.228573
89	Crk-Itgb1	0.228573
90	Pdia3-Plxna1	0.24444
91	Cst3-Itgb1	0.24444
92	Crk-Rasl10a	0.24845
93	B2m-Glrb	0.253351
94	B2m-Cx3cl1	0.254645
95	Crk-Ghr	0.258292
96	Crk-Dok3	0.260246
97	Crk-Mast1	0.260246
98	App-Itgb5	0.263273
99	App-Rab3d	0.271175
100	Lgals1-Ptprc	0.275992
101	Crk-Elmo1	0.278453
102	Igfbp5-Ager	0.283591
103	B2m-Ghr	0.284903
104	Cst3-Ghr	0.288406
105	Nme2-Rab5a	0.291677
106	App-Dgkz	0.291677
107	Crk-Csf1	0.295497
108	App-Mas1	0.299237
109	Arcn1-Rab18	0.304528
110	B2m-Cd79b	0.306351
111	Myl12b-Sri	0.307302
112	Crk-Dgkz	0.307302
113	Crk-Gab2	0.309858
114	App-Rhoq	0.315225
115	B2m-Rhoq	0.320629
116	App-Itsn1	0.320629
117	App-Csf1	0.322867
118	App-Rab39b	0.322867
119	Crk-Rhoq	0.32607
120	Cfl1-G3bp1	0.332704
121	Crk-Rhoc	0.332704
122	Crk-G3bp1	0.336587
123	App-Dlg4	0.338371
124	B2m-Csf1	0.338371
125	Crk-Rab39b	0.338371
126	Crk-Rgs20	0.340501
127	Igfbp5-Prkca	0.342614
128	Crk-Rhod	0.342614
129	B2m-Itgb2	0.348201
130	Crk-Rab3d	0.348419
131	Cdh2-Sipa1l1	0.352424
132	Crk-Ptk2b	0.353816
133	Igfbp5-Pdgfra	0.353824
134	Cfl1-Sipa1l1	0.356459
135	Igfbp5-Pdgfrb	0.365178
136	Mapre1-Rock2	0.3692
137	App-Itpr3	0.3692
138	App-Anxa1	0.376672
139	Arcn1-Dnm2	0.38132
140	Crk-Dnm2	0.385573
141	Igf1-Pdgfra	0.388305
142	App-Rras	0.388305
143	App-Dnm2	0.389857
144	Crk-Gem	0.389857
145	Cst3-Dner	0.394173
146	App-G3bp1	0.39852
147	Cst3-Il6ra	0.399791
148	App-Cx3cl1	0.399791
149	App-Apbb1ip	0.399791
150	Cdh2-Celsr2	0.400075
151	Ccn2-Sri	0.411754
152	Cst3-Itgb3	0.414997
153	Pdia3-Sri	0.416229
154	Hp-Sri	0.420737
155	B2m-Cd3e	0.424019
156	Hmox1-Sri	0.425278
157	Sod2-Sri	0.42985
158	Cfl1-Sdcbp	0.42985
159	App-Nod1	0.430143
160	Arcn1-Sdcbp	0.434456
161	Crk-Rab40b	0.434456
162	Crk-Sdcbp	0.439094
163	App-Rab40b	0.439094
164	App-Ephb3	0.442324
165	Pdia3-Sdcbp	0.443765
166	Cdh2-L1cam	0.44523
167	Igf1-Nod1	0.44523
168	Crk-Rasgrp1	0.453207
169	App-Gabra4	0.453207
170	Crk-Fcgr2b	0.462781
171	B2m-Fcgr2b	0.467619
172	B2m-Ifnar2	0.47249
173	App-Aplp2	0.47249
174	Nme2-Tbxa2r	0.475227
175	Cst3-Pdgfra	0.479796
176	B2m-Cd274	0.482333
177	Hp-Itgam	0.482333
178	Cfl1-Rab11a	0.486151
179	Crk-Fgfr1	0.490137
180	Igf1-Pdgfrb	0.497353
181	Cfl1-Rab10	0.504989
182	App-Adrb1	0.504989
183	Lgals3-Lpar1	0.511872
184	Mapre1-Mib1	0.519781
185	B2m-Arhgap10	0.533604
186	App-Slc7a1	0.534515
187	Crk-Arhgap10	0.538922
188	App-Arrb1	0.544276
189	Cdh2-Ddr1	0.549191
190	App-Hbegf	0.549191
191	Arcn1-Rab11a	0.551339
192	Hmox1-Ptprc	0.558017
193	App-Mark2	0.563808
194	Cdh2-Ptprc	0.564723
195	B2m-Inpp5d	0.564723
196	App-Gpr12	0.566044
197	Cst3-Inpp5d	0.571457
198	B2m-Ptprc	0.571457
199	Crk-Mark2	0.578367
200	Crk-Rras2	0.582746
201	App-Itga6	0.598666
202	Hnrnpk-Homer2	0.599774
203	App-Rhoj	0.60731
204	App-Kcnk2	0.60731
205	Crk-Grasp	0.61243
206	Crk-Rhoj	0.621695
207	Crk-Mras	0.622988
208	Crk-Rab14	0.6263
209	App-Il1rl1	0.628884
210	Arcn1-Rab14	0.633273
211	B2m-Il1rl1	0.634817
212	Hnrnpf-Rab14	0.640272
213	Igf1-Il1rl1	0.640787
214	Nme1-Rrad	0.650291
215	App-Adam9	0.654345
216	B2m-Gabra4	0.658921
217	B2m-Sdcbp	0.665041
218	Crk-Rab11a	0.668517
219	Crk-Rasa4	0.671199
220	App-Grm5	0.67564
221	App-Gabra2	0.678658
222	App-Cdh13	0.689957
223	App-Bcl10	0.689957
224	B2m-Gabra2	0.692755
225	Cfl1-Traf4	0.69621
226	Nme1-Traf4	0.702558
227	Cdh2-Epha4	0.702558
228	App-Rgs17	0.706795
229	Park7-Traf4	0.708945
230	Hnrnpk-Sdcbp	0.708945
231	Gaa-Traf4	0.715371
232	Cdh2-Iqgap1	0.718873
233	Crk-Rgs17	0.720778
234	App-Rab18	0.720778
235	Hnrnpk-Rab18	0.734704
236	Mtpn-Pdpk1	0.741461
237	App-Itpr1	0.741461
238	Crk-Pdpk1	0.748081
239	Arcn1-Rab5a	0.748573
240	Crk-Rab18	0.748573
241	App-Tulp3	0.748573
242	Mapre1-Pdpk1	0.75474
243	Crk-Iqgap1	0.75474
244	App-Pdpk1	0.761439
245	Cst3-Pdpk1	0.768177
246	App-Furin	0.770339
247	B2m-Rab29	0.789841
248	App-Adcy9	0.789841
249	Crk-Rab29	0.795528
250	App-Chrna4	0.803483
251	B2m-Gabre	0.807737
252	Hnrnpk-Magi3	0.807737
253	B2m-Fgfr1	0.809444
254	B2m-Cd14	0.809444
255	Crk-Rgs14	0.815277
256	Cdh2-Fgfr1	0.816462
257	B2m-Ager	0.81707
258	App-Adcy2	0.81707
259	Cfl1-Fgfr1	0.823521
260	App-Ager	0.830601
261	App-Slc9a3r1	0.830601
262	Cst3-Fgfr1	0.83062
263	Ccn2-Fgfr1	0.83776
264	App-Gabrb3	0.857494
265	App-Rhoc	0.860922
266	App-Adap2	0.866729
267	Cdh2-Prkca	0.868594
268	B2m-Rhoc	0.868594
269	B2m-Gabrb3	0.870856
270	App-Kitl	0.870856
271	Cfl1-Rala	0.883994
272	Crk-Sipa1l1	0.888889
273	Crk-Rala	0.89172
274	Hnrnpk-Rala	0.896359
275	Hmox1-Rala	0.899463
276	App-Rab3b	0.899463
277	Mapre1-Rala	0.90387
278	App-Cav1	0.907223
279	App-Dner	0.910611
280	Crk-Rab9	0.922795
281	B2m-Gp6	0.923752
282	Crk-Traf4	0.926655
283	Nme1-Inpp5d	0.930605
284	App-Tnfrsf19	0.936838
285	Crk-Ralgps2	0.942057
286	B2m-Ptk2b	0.949821
287	B2m-Tnfrsf19	0.949868
288	App-Ptk2b	0.957627
289	B2m-Gabra3	0.957627
290	App-Pdgfra	0.962844
291	Ccl3-Ptk2b	0.965477
292	App-Epha4	0.965477
293	Cfl1-Ptk2b	0.973369
294	B2m-Gabrr2	0.981304
295	App-Tnfsf13b	0.997303
296	Crk-Ifnar2	0.997303
297	B2m-Nfam1	1
298	Ccn2-Ror2	1
299	Igfbp5-Itgb3	1
300	Cfl1-Rab35	1
301	Lgals1-Rac1	1
302	B2m-Sla2	1
303	Mapre1-Ube2b	1
304	Igf1-Sla2	1
305	Nme1-Rac1	1
306	B2m-Il6ra	1
307	Hmox1-Il6ra	1
308	Cst3-Grin2c	1
309	Igf1-P2ry6	1
310	Prdx6-Rhoa	1
311	Prdx6b-Rhoa	1
312	Ccn2-Il6ra	1
313	Lgals1-Rab10	1
314	Pdia3-Rab5a	1
315	Crk-Rab5a	1
316	Igf1-Skap1	1
317	Igf1-Avpr1a	1
318	Cdh2-Itgb3	1
319	Cfl1-Rhoa	1
320	Cfl1-Rock2	1
321	Crk-Rhoa	1
322	Crk-Rock2	1
323	Cnbp-Rab5a	1
324	Park7-Rac1	1
325	Crk-Rab35	1
326	Arcn1-Rab10	1
327	Crk-Rab10	1
328	App-Gnaz	1
329	Mapre1-Rac1	1
330	Cfl1-Gpr65	1
331	B2m-Fgd2	1
332	Hmox1-Sla2	1
333	Crk-Arhgap33	1
334	Nme2-Tgfbr1	1
335	Park7-Rhoa	1
336	Cdh2-Ptprt	1
337	Crk-Rac1	1
338	Pdia3-Rab11a	1
339	Crk-Rit1	1
340	Igf1-Itgb3	1
341	Cdh2-Dlg4	1
342	Igf1-Inpp5d	1
343	Crk-Skap1	1
344	Cdh2-Grin2b	1
345	Igf1-Rhoa	1
346	App-Rab10	1
347	Igf1-Rac1	1
348	Mapre1-Rab11a	1
349	Igf1-Prkca	1
350	B2m-Skap1	1
351	Hmox1-Inpp5d	1
352	Crk-Inpp5d	1
353	Prdx5-Ube2b	1
354	Sec22b-Ube2b	1
355	Cnbp-Rab11a	1
356	B2m-Adgra3	1
357	B2m-Ube2b	1
358	Cst3-Rac1	1
359	Arcn1-Rac1	1
360	Cfl1-Rab14	1
361	App-Skap1	1
362	Hnrnpk-Rhoa	1
363	B2m-Gab2	1
364	Prdx6-Rac1	1
365	Prdx6b-Rac1	1
366	Nme2-Inpp5d	1
367	App-Rab14	1
368	B2m-Ly6e	1
369	App-Rab35	1
370	Mapre1-Tgfbr1	1
371	Arcn1-Rhoa	1
372	Cfl1-Rac1	1
373	Cst3-Rhoa	1
374	Gfap-Prkca	1
375	Crk-Sla2	1
376	B2m-Spn	1
377	App-Rasl10b	1
378	Ccn2-Tgfbr1	1
379	Lgals3-Tgfbr1	1
380	Ltbp2-Tgfbr1	1
381	Crk-Rgs19	1
382	B2m-Adgra2	1
383	Cdh2-Rab5a	1
384	Hnrnpk-Rab5a	1
385	Hnrnpk-Rab14	1
386	Ccn2-Prkca	1
387	Arcn1-Tgfbr1	1
388	Crk-Rasl10b	1
389	Crk-Plek2	1
390	Cnbp-Ube2b	1
391	Sod1-Tgfbr1	1
392	App-Prkca	1
393	Crk-Usp8	1
394	Lgals1-Rhoa	1
395	Hnrnpk-Rab11a	1
396	App-Lancl2	1
397	B2m-Rab9	1
398	Cst3-Tgfbr1	1
399	Sod2-Tgfbr1	1
400	Mtpn-Rhoa	1
401	B2m-Rit1	1
402	Crk-Fgd2	1
403	Igf1-Tgfbr1	1
404	Cst3-Prkca	1
405	B2m-Rasgrf2	1
406	Crk-Rasgrf2	1
407	Crk-Gpr65	1
408	Cdh2-Inpp5d	1
409	App-Grin2b	1
410	Hnrnpk-Tgfbr1	1
411	Crk-Ube2b	1
412	Igfbp2-Prkca	1
413	Crk-L1cam	1
414	App-Drd1	1
415	Igfbp5-Tgfbr1	1
416	B2m-Gabrr1	1
417	Crk-Tgfbr1	1
418	Cfl1-Inpp5d	1
419	Cdh2-Rhoa	1
420	Igfbp2-Tgfbr1	1
421	Crk-Rrad	1
422	Crk-Grin2b	1
423	Mtpn-Tgfbr1	1
424	Crk-Rgs9	1
425	App-Rab11a	1
426	App-Arap3	1
427	Crk-Lancl2	1
428	Arcn1-Prkca	1
429	App-Sipa1l1	1
430	B2m-C3ar1	1
431	Crk-Rasd2	1
432	App-Gabra3	1
433	Sec22b-Tgfbr1	1
434	B2m-Celsr2	1
435	Crk-Il6ra	1
436	Crk-Lpar1	1
437	App-Chrnb1	1
438	App-Fgfr1	1
439	App-Rhoa	1
440	App-Grik2	1
441	App-L1cam	1
442	Crk-Prkca	1
443	B2m-Rab10	1
444	Pea15a-Tgfbr1	1
445	Crk-Rab9b	1
446	App-Celsr2	1
447	Crk-Atp6ap2	1
448	Lgals1-Tgfbr1	1
449	App-Rasgrf2	1
450	App-Ghr	1
451	Crk-Gna11	1
452	Crk-Rgs6	1
453	Lxn-Tgfbr1	1
454	App-Grik4	1
455	App-Tgfbr1	1
456	App-Unc5b	1
457	App-Hrh3	1
458	Crk-Magi3	1
459	App-Fgd2	1
460	Crk-Ptprc	1
461	App-Bmpr2	1
462	App-Avpr1a	1
463	App-Rab5a	1
464	App-Unc5a	1
465	App-Arhgap10	1
466	B2m-Rab5a	1
467	Cfl1-Tgfbr1	1
468	App-Bdkrb2	1
469	B2m-Rac1	1
470	B2m-Tgfbr1	1
471	B2m-Prkca	1
472	App-Traf4	1
473	App-C3ar1	1
474	App-Fzd5	1
475	App-Ptprc	1
476	App-Sdcbp	1
477	App-Mib1	1
478	App-Lpar1	1
479	App-Rala	1
480	App-Rgs6	1
481	App-Mras	1
482	App-Trpv4	1
483	App-Elmo1	1
484	App-Gabrr2	1
485	App-Chrng	1
486	App-Rock2	1
487	App-Inpp5d	1
488	App-Pdgfrb	1
489	App-Nmur1	1
490	App-Usp8	1
491	App-Sla2	1
492	App-Rasd2	1
493	App-Jag2	1
494	App-Rgs19	1
495	App-Rgs20	1
496	App-Dok3	1
497	App-Adcy4	1
498	App-Ly6e	1
499	App-Acvrl1	1
500	App-Ptprt	1
501	App-Il6ra	1
502	App-Npffr2	1
503	App-P2ry4	1
504	App-Itk	1
505	App-Gna11	1
506	App-Ddr1	1
507	App-Chrna6	1
508	App-Adcy3	1
509	App-Epha1	1
510	App-Plxna1	1
511	App-Ube2b	1
512	App-Csf1r	1
513	App-Magi3	1
514	App-Sri	1
515	App-Cd79b	1
516	App-Itgam	1
517	App-Ror2	1
518	App-Unc13a	1
519	App-Cd274	1
520	App-Lpar6	1
521	App-Olfr187	1
522	App-C5ar2	1
523	App-Rgs9	1
524	App-Grin2c	1
525	App-Tnfsf12	1
526	App-Iqgap1	1
527	App-Grm1	1
528	App-Cd3e	1
529	App-Olfr1444	1
530	App-Rit1	1
531	App-Rac1	1
532	App-Gabre	1
533	App-Rab9	1
534	App-Oprl1	1
535	App-Olfr1052	1
536	App-Arhgap33	1
537	App-Rras2	1

### APP is a putative toxic factor

**Fig. 3:**
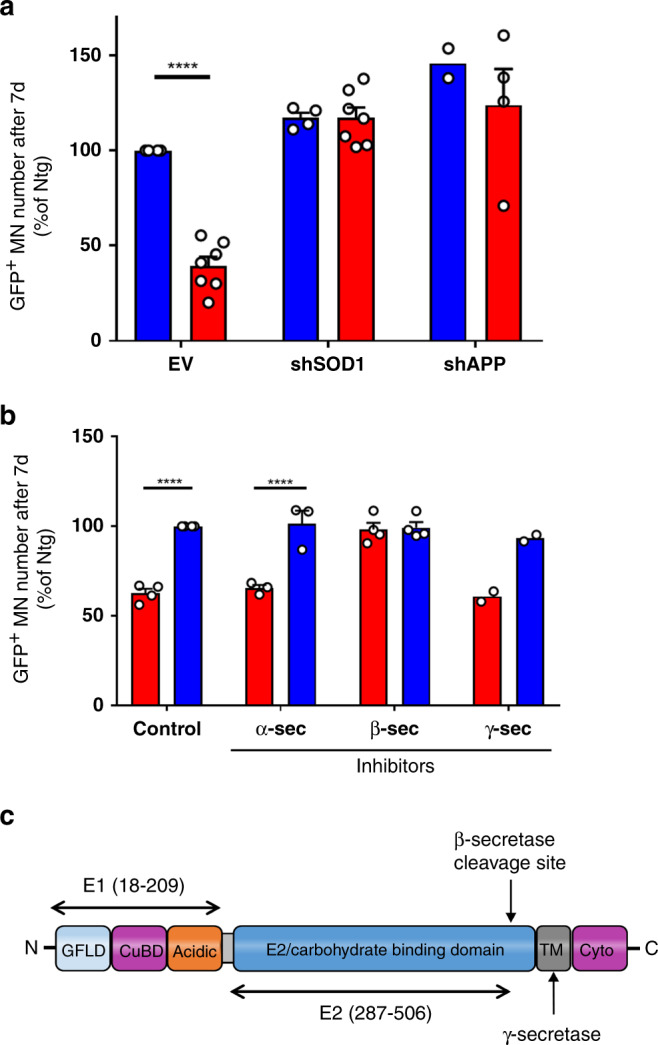
mutSOD1 astrocytes release β-secretase sensitive N-APP fragments that cause toxicity in ALS in vitro models. **a** NTg (blue) or mutSOD1 (red) astrocytes infected with empty vector (EV) (*n* = 4 NTg and *n* = 7 mutSOD1), sh-SOD1 (*n* = 4 NTg and *n* = 7 mutSOD1) or sh-APP (*n* = 2 NTg and *n* = 4 mutSOD1) selected with puromycin for 4.5 days, replaced with regular astrocyte media and then were co-cultured with mouse ES-MNs expressing EGFP under the control of the MN-specific Hb9 promoter. Viability was measured at 7 DIV. MNs were counted using Metamorph software. Data for EV and sh-SOD1 are means ± SEM of independent experiments (*n*) and were analyzed by a two-way ANOVA (Interaction F_(2,22)_ = 7.824; *P* = 0.0027) followed by a Sidak post hoc test: ****P ≤ 0.0001 EV NTg vs mutSOD1 (CI: 32.34–88.86%; *d* = 7.29). Data for sh-APP in NTg (*n* = 2) are means only and were not analyzed by statistics. **b** Inhibitors of α-secretase (*n* = 3), β-secretase (*n* = 4), γ-secretase (*n* = 2), or vehicle control (*n* =4) were applied twice (1 DIV and 4 DIV) at 5 μM, 250 nM, and 500 nM, respectively to primary MN and GFP+ MNs were counted on DIV 7. Data for control, α-secretase and β-secretase are means ± SEM of independent experiments (*n*) and were analyzed by a two-way ANOVA (Interaction F_(3,18)_ = 13.97; *P* < 0.0001) followed by a Sidak post hoc test: ****P ≤ 0.0001 Control NTg vs mutSOD1 (CI: 24.9–49.69%; *d* = 12.27) and α-secretase NTg vs mutSOD1 (CI: 21.67–50.29%; *d* = 4.83). Data for γ-secretase in NTg (*n* = 2) are mean only and were not analyzed by statistics. **c** Schematic figure of protein domains of APP and E1 and E2 segments. See also Supplementary Figs. 2 and 3. Source data provided as source data file.

To validate the contribution of APP in MN degeneration in our in vitro models of ALS, we assessed its expression in astrocytes and found that the *APP* mRNA levels were 128 ± 12% (mean ± SEM of *n* = 3 independent experiments; 95% confident interval [CI]: 105–152%) higher in mutSOD1 than in NTg astrocytes. Next, we targeted APP expression in these cultured astrocytes. We found that silencing APP expression in astrocytes using lentiviral vector *APP* shRNAs (efficiency in two independent experiments: 77% and 61% knockdown in NTg astrocytes and 74% and 57% in mutSOD1 astrocytes) abrogated MN toxicity of mutSOD1 astrocyte monolayer (AML) (Fig. 3a). In light of the above and our previous demonstration that silencing SOD1 in mutSOD1 astrocytes also protected against the degeneration of cultured MNs^9^, we wondered whether SOD1 can modulate APP expression. However, despite a robust knockdown of SOD1 (mean ± SEM: 74.8 ± 6.5% of *n* = 3 independent experiments; CI: 62.1–87.5%), we found no change in APP expression (mean ± SEM: 109.5 ± 11.8% of *n* = 3 independent experiments; CI: 86.4–133.0%) in mutSOD1 astrocytes.

Since ACM can reproduce the toxicity of mutSOD1 astrocytes, we next reasoned that the contribution of APP to MN toxicity might be related to a soluble fragment of the protein shed from its native membrane location to the culture medium. Accordingly, we decided to assess the role of α, β, and γ secretases, which are the three main enzymatic families known to cleave full-length APP and generate soluble fragments in the amyloidogenic or non-amyloidogenic APP metabolism pathways^35^. We found that β-secretase inhibition prevented MN death (Fig. 3b), a protective effect that was not explained by an alteration of mutSOD1 expression since a knockdown of β-secretase by ~80% (*n* = 2 independent experiments) did not alter SOD1 expression in astrocytes. In contrast, neither inhibition of α-secretase or γ-secretase protected against MN death (Fig. 3b). These results indicate that the β-soluble, N-terminal fragment of APP (sAPP-β) might be responsible for the demise of MNs (Fig. 3b). However, we know that sAPP-β has a molecular mass of ~100-kDa while the astrocyte toxic factor, based on centrifugal filtration, is most likely ≤30-kDa (Fig. 1b). This suggests that a smaller fragment, rather than full-length of sAPP-β, mediates MN death (Fig. 3c), an idea we thought to test in our in vitro system. To do so and given the different sequence permutations of ~30-kDa within sAPP-β, we elected to examine the effects of two highly conserved domains of sAPP-β—the E1-domain (Leu18-Ala190) and the E2-domain (Ser295-Asp500)^36^ in cultured ES-MNs. Over a period of 5 days, we found that the numbers of mouse ES-MNs cultures incubated from 2 to 7 DIV with 3 μM E1 or E2 recombinant declined significantly (Supplementary Fig. 2); a similar trend of decline was observed with 1 μM, but not with concentrations ≤0.5 μM of recombinants. These results provide impetus to using this bioassay for future studies aimed at refining our search for the sequence of the putative smaller fragments of sAPP-β.

At last, since β-secretase can cleave APP and closely related proteins APLP1 and APLP2^37,38^, we also assessed the effect of APLP1 and APLP2 silencing in mutSOD1 astrocytes on MN death. These experiments demonstrated that silencing APLP1 in astrocytes phenocopied the protective effect of APP silencing. However, silencing APLP2 in astrocytes had no effect (Supplementary Fig. 3). Thus, these findings suggest that both sAPP-β and sAPLP1-β may both contribute to astrocyte-mediated MN degeneration.

### DR6 triggers astrocyte-mediated death signal in MNs

**Fig. 4:**
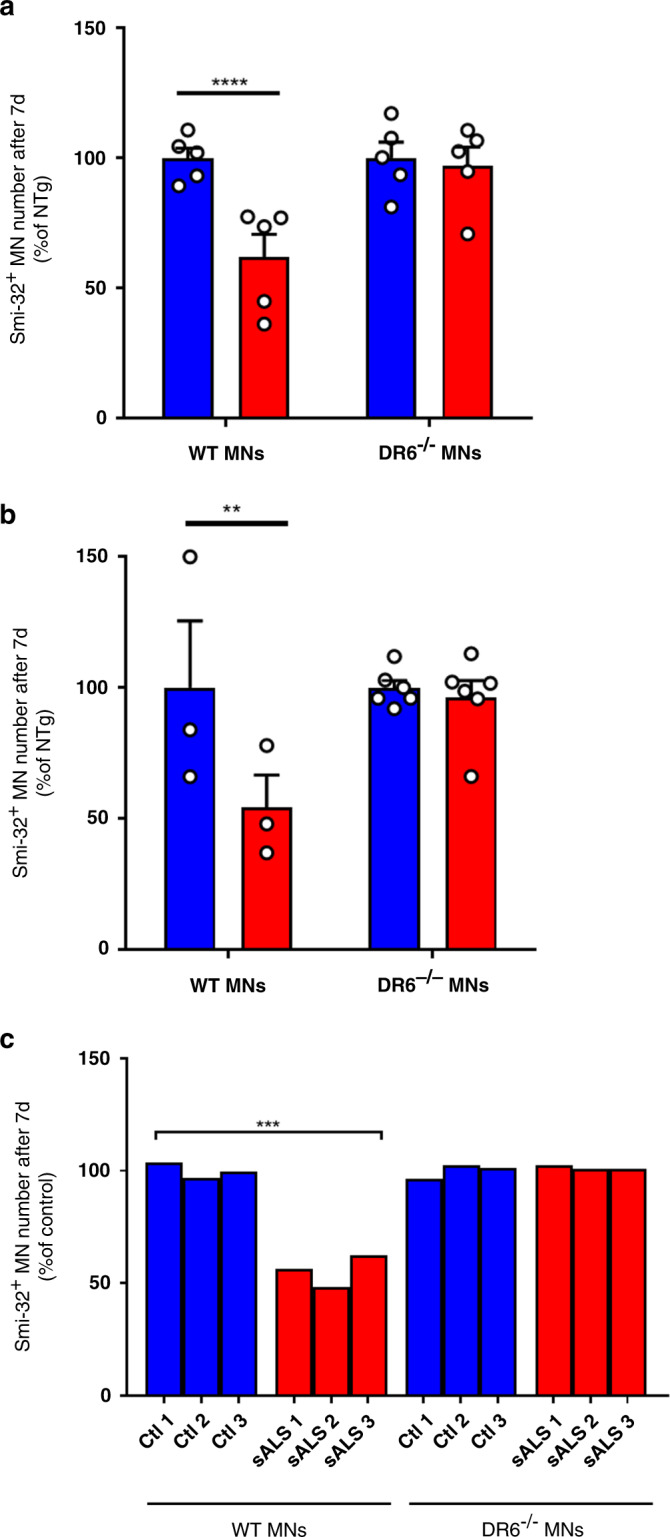
mutSOD1 and sALS astrocytes mediate MN death via a DR6-dependent mechanism. **a** Co-culture of mouse WT or DR6^−/−^ MNs with astrocytes from NTg (blue, *n* = 5) or mutSOD1 (red, *n* = 5) mice. Data are means ± SEM of independent experiments (*n*) analyzed by two-way ANOVA (Interaction F_(1,16)_ = 6.903; *P* = 0.0183) followed by Sidak post hoc test: **P = 0.0020 WT neurons/NTg AML vs WT neurons/mutSOD1 AML (CI: 14.69–61.54%; *d* = 4.94). **b** WT (*n* = 3) or DR6^−/−^ MNs (*n* = 6) were cultured in the presence of ACM from astrocytes from NTg (blue) or Tg mutSOD1 (red) mice. Data are means ± SEM of independent experiments (*n*) analyzed by repeated measures two-way ANOVA (Interaction F_(1,7)_ = 12.68; *P* = 0.0092) followed by Sidak post hoc test: **P = 0.0042 WT neurons with NTg ACM vs mutSOD1 ACM (CI: 18.35–72.99%; *d* = 1.61). **c** Astrocytes from three independent human controls or three sALS patients were co-cultured with either WT or DR6^−/−^ MNs. The cells were then fixed and imaged using the neuronal marker SMI-32 **a**–**c** and counted. Individual data points are plotted and data were analyzed using two-tailed Student’s *t* test: WT Control vs sALS astrocytes (*t*_(10)_ = 5.943, ***P = 0.0001; CI: −61.87% to −28.13%; *d* = 3.19). See also Supplementary Figs. 4 and 5. Source data provided as source data file.

Next, we sought to assess the role of DR6 in the death of MNs in response to mutSOD1 astrocytes. We cultured spinal primary MNs from both mutant mice deficient in DR6^39^ and their WT littermates. DR6^−/−^ mice had a normal lifespan, bred and gained weight similarly to their WT littermates. Furthermore, DR6^−/−^ spinal MNs survived similarly as their WT MN counterparts on NTg AML as assessed at 7 DIV (Fig. 4a and Supplementary Fig. 4). As previously shown, we found that the numbers of WT MNs exposed to either mutSOD1 AML or ACM were reduced to ~50% compared with those exposed to NTg AML or ACM at 7 DIV (Fig. 4a, b). In contrast, the numbers of DR6^−/−^ MNs exposed to either mutSOD1 AML or ACM were not significantly different to those exposed to NTg AML or ACM at 7 DIV (Fig. 4a, b). Likewise, the numbers of WT mouse MNs exposed to adult human astrocytes from sALS patients were significantly lower than those exposed to non-diseased control astrocytes, whereas there was no significant difference in the numbers of DR6^−/−^ mouse MNs exposed to either sALS or non-diseased astrocytes (Fig. 4c). At last, since DR6 can heterodimerize with TNFRSF16/P75^NTR^^40^, we also assessed the contribution of this other receptor in MN death. P75^NTR^ neutralizing antibodies^18,41^ had no effect on mutSOD1 astrocyte-induced MN toxicity (Supplementary Fig. 5a) and, based on a single experiment, mouse MNs deficient in this receptor appeared as susceptible to mutSOD1 ACM as their WT MN counterparts (Supplementary Fig. 5b), hence excluding a role for P75^NTR^ herein. Thus, our findings indicate that DR6, but not P75^NTR^, play a critical role in the demise of MNs in our in vitro models of ALS.

### Knockdown of DR6 expression in mutSOD1 mice attenuates MN death

**Fig. 5:**
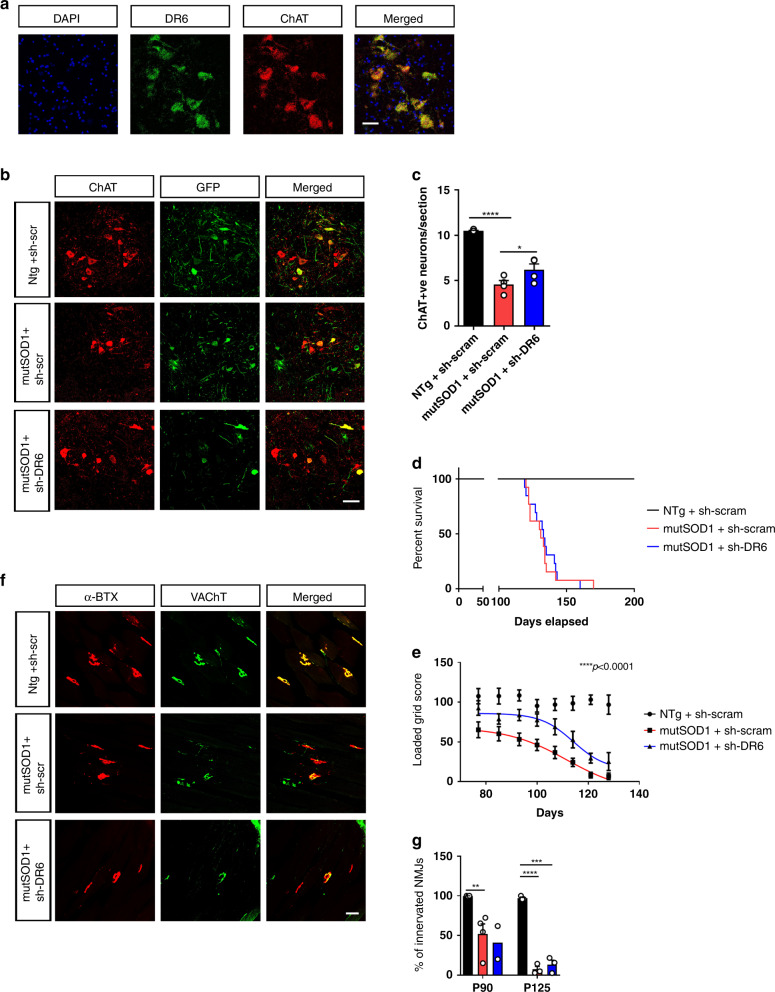
ICV injection of shDR6 in SOD1^G93A^ mice leads to partial recovery of ALS model. **a** Unfixed 20 μm-thick L4/L5 section of SC from P50 WT mice were labeled with mRNA-based fluorescent probes for DR6 and ChAT using RNAscope technique. Representative images of three experiments repeated independently with similar results. Scale bar: 50 μm. **b** L4–L5 of spinal cord of the P125 mice were frozen and cut into 15 µm-thick sections with a cryostat, visualized by immunohistochemistry using ChAT antibody. Scale bar: 100 μm. The sections were imaged on Leica confocal microscope. Representative images are shown. **c** ChAT stained neurons in each ventral horn hemi-section were manually counted and quantified. Data are means ± SEM of *n* = 4 independent experiments and were analyzed by one-way ANOVA (F_(2,9)_ = 43.85; *P* < 0.0001) followed by a Neuman–Keuls post hoc test: ****P < 0.0001 NTg+sh-scram vs mutSOD1+sh-scram (*d* = 10.43) and NTg+sh-scram vs mutSOD1+shDR6 (*d* = 5.34); *P = 0.034 mutSOD1+sh-scram vs mutSOD1+shDR6 (*d* = 1.63). **d** Survival of NTg mice (*n* = 10) and Tg mutSOD1 mice injected with sh-scram (*n* = 13) or shDR6 (*n* = 13) over time. Data were analyzed by Kaplan–Meier estimator with Log rank test which revealed statistical significant difference in survival between NTg vs Tg mutSOD1 mice injected with sh-scram or shDR6 (*P* < 0.0001, df = 2). However, log rank test revealed no statistical significant difference in survival between Tg mutSOD1 mice injected with sh-scram compared to Tg mutSOD1 mice injected with shDR6. **e** Loaded grid assay was performed in NTg (*n* = 10 mice) and Tg mutSOD1 mice injected with sh-scram (*n* = 19 mice) or shDR6 (*n* = 16 mice) over the course of the disease. Data were analyzed by non-linear regression curve fit using least sum-of-squares method and revealed that shDR6-injected Tg mutSOD1 mice motor performance is significantly different than sh-scram injected Tg mutSOD1 mice (F_(4,234)_ = 11.41; *P* < 0.0001). **f** Tibialis anterior muscle from NTg mice (black) and Tg mutSOD1 mice injected with sh-scram (red) or shDR6 (blue) was processed and incubated with α-bungarotoxin-594, anti-neurofilament and anti-synaptophysin antibodies. Representative images are shown. Scale bar: 50 μm. **g** Quantitative assessment of muscle innervation was done by counting at least 50 neuromuscular junctions (NMJs) by first imaging α-bungarotoxin-594 and then confirming innervation using anti-neurofilament. Studies were done at P90 in NTg mice injected with sh-scram (*n* = 3) and Tg mutSOD1 mice injected with sh-scram (*n* = 4) or shDR6 (*n* = 2) and at P125, in NTg mice injected with sh-scram (*n* = 3) and Tg mutSOD1 mice injected with sh-scram (*n* = 3) or shDR6 (*n* = 3). Data are means ± SEM of independent mice (*n*) and were analyzed by two-way ANOVA (genotype main factor F_(2, 12)_ = 37.26; *P* < 0.0001 with unbalanced design followed by Sidak post hoc test: **P = 0.0064 P90 NTg sh-scram vs Tg mutSOD1 sh-scram (CI: 13.79–81.81%; *d* = 3.07); ****P ≤ 0.0001 P125 NTg sh-scrambled vs Tg mutSOD1 sh-scrambled (CI: 53.25–126%; *d* = 23.40); ***P = 0.0001 P125 NTg sh-scram vs Tg mutSOD1 shDR6 (CI: 47.23–119.9%; *d* = 14.84). See also Supplementary Figs. 6 and 7. Source data provided as source data file.

Given our demonstration that DR6 likely drives the death of MNs in vitro in response to both cultured mouse mutSOD1 and human sALS astrocytes (Fig. 4a–c), we asked whether targeting this receptor would alter the disease phenotype in Tg mutSOD1 mice. As a prerequisite, we assessed the expression of DR6 in spinal MNs. Using multiplex RNAscope technology, we found that 86.0 ± 6.1% (mean ± SEM; CI: 74–98%) of MNs expressing choline acetyltransferase (ChAT), in SC sections from three mice, also expressed DR6 (Fig. 5a), indicating that most spinal MNs express this death receptor. In view of these results and since we sought to interrogate the specific role played by DR6 on spinal MNs, rather than using the constitutive DR6^−/−^ mice, we decided to conditionally silence DR6 in MNs by gene therapy. As done before^42^, we performed an intracerebroventricular (ICV) injection of an adeno-associated serotype 9 (AAV9-U6-shRNA-CMV-GFP) viral vector expressing a DR6-shRNA or scrambled shRNA into mice at postnatal day 1. Consistent with previous data with ICV injection of AAV9 viral vector^42,43^, we found that ∼60% of spinal MNs were successfully transduced as evidenced by the co-localization of GFP and ChAT (Fig. 5b and Supplementary Fig. 6A). Furthermore, we confirmed the silencing of DR6 by quantitative reverse transcription PCR (qRT-PCR) and observed approximately twofold reduction of DR6 mRNA levels in SC extracts of adult mice injected with the shRNA-DR6 viral vector (Supplementary Fig. 6B). However, since MNs represent only a fraction of the DR6-expressing cells in SC and that ~60% of the MNs were transduced, the actual silencing of DR6 in MNs is likely more substantial.

Despite the partial transduction of SC MN population, Tg mutSOD1 mice injected with DR6-shRNA showed significantly more surviving lumbar MNs compared with age-matched Tg mutSOD1 mice injected with shRNA scramble (Fig. 5b, c). Yet, no significant difference was observed in the age of animals either when they reached 10% body weight loss or at end-stage between the two experimental groups of Tg mutSOD1 mice (Fig. 5d and Supplementary Fig. 7A). Since, in ALS mice, body weight may be used as a proxy of global muscle innervation^44^ and lifespan as a proxy of global wellbeing, we sought to investigate the status of select motor units. To do so, we performed a loaded grid assay, which specifically reflects limb muscle strength, and found that the motor performance in DR6-shRNA injected mice was significantly better preserved than in scrambled-shRNA injected mice (Fig. 5e). Intriguingly, the extent of neuromuscular junction (NMJ) denervation in the tibialis anterior, an affected muscle in Tg mutSOD1 mice^45^, was not significantly different between the two groups of mice (Fig. 5f, g). Since limb muscle strength relies not only on tibialis anterior, it is possible that the observed behavioral benefit results from the knockdown of DR6, attenuating the denervation of other limb muscles such as gastrocnemius.

### DR6 expression parallels that of MMP9 in spinal MNs

**Fig. 6:**
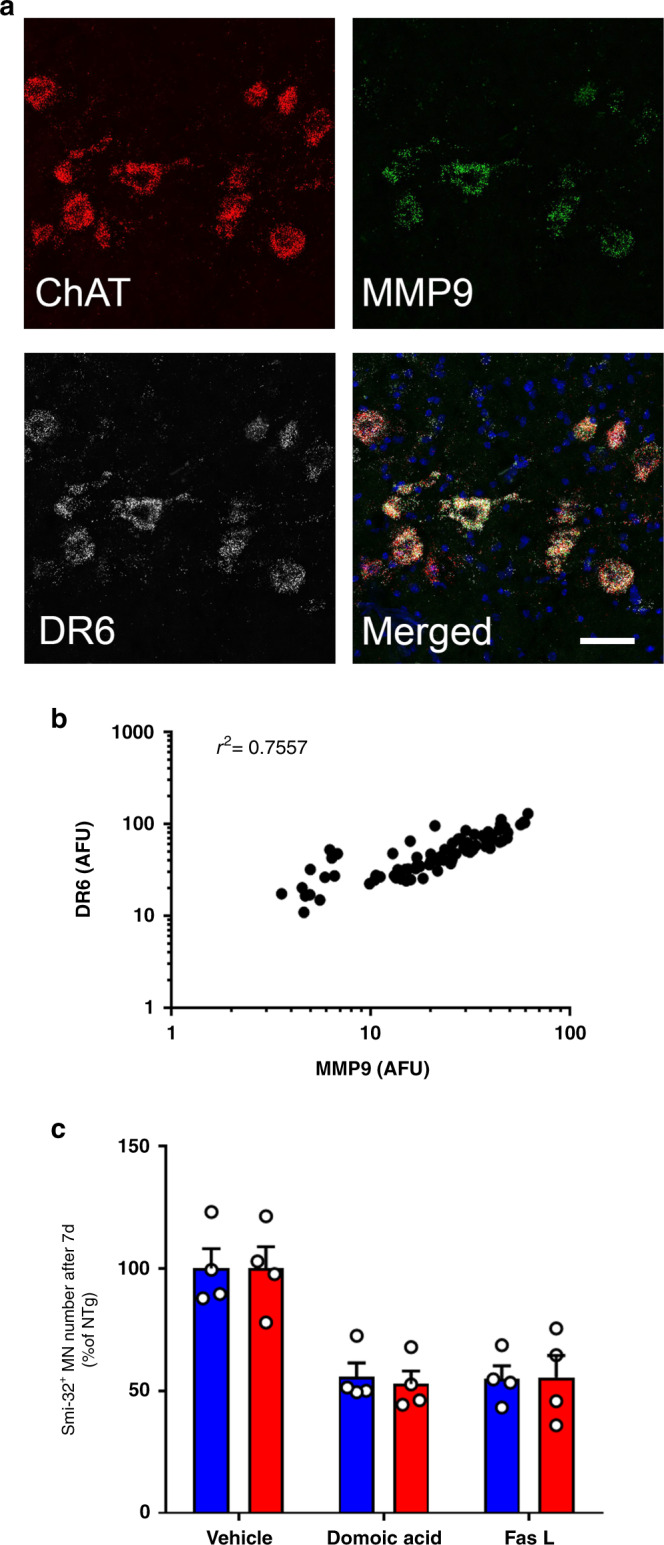
MN toxicity susceptibility marker MMP9 positively correlates with DR6. **a** 20 μm thick unfixed L4/L5 sections of spinal cord from WT mice were labeled with fluorescent probes for DR6, MMP9, and ChAT using RNAscope technique. Representative images are shown. Scale bar: 50 μm. **b** Pearson’s linear correlation analysis was performed between DR6 and MMP9 in ChAT-positive MNs (r_(75)_ = 0.8693, CI 0.8013–0.9151, two-tailed *P* < 0.0001). Similar *r* values were obtained for two additional independent experiments. Representative correlation plot of three experiments repeated independently with similar results. **c** Mouse WT (blue) or DR6^−/−^ (red) MNs were treated with vehicle, Domoic acid or activating anti-Fas antibody for 7 days. Data are means ± SEM of *n* = 4 independent experiments and were analyzed by two-way ANOVA (interaction F_(2,18)_ = 0.036, *P* = 0.96), which revealed no statistical differences for genotypes x treatments. Source data provided as source data file.

At last, since not all spinal MNs degenerate in either ALS or its mouse models^45^, we wondered if DR6 expression levels may contribute to the differential vulnerability of MNs to the disease process. Notably, we found that the mean coefficient of variability of DR6 fluorescent signal in spinal MNs from three mice (mean ± SEM: 51.4 ± 3.5%; CI: 44.5–58.3%) was significantly greater (two-tailed Student’s *t* test: *t*_[4]_ = 5.72; *p* = 0.0046; Cohen’s *d* effect size [*d*] = 4.77) than that of ChAT (mean ± SEM: 29.8 ± 1.2%; CI: 27.4–32.1%), supporting our hypothesis that MNs do indeed express variable levels of DR6. To examine this question further, we sought to relate the transcript levels of DR6 to those of the matrix metallopeptidase 9 (MMP9), a previously reported marker of MN differential susceptibility^46^. Strikingly, three independent experiments revealed a significant positive correlation between DR6 and MMP9 mRNA fluorescent signals in spinal MNs (Fig. 6a, b). Thus, these results indicate that most spinal MNs express DR6, albeit to different levels, and that MNs with the highest expression of DR6 happen to also have the highest expression of MMP9.

In light of the above findings, we asked whether, akin to MMP9 ref. ^46^, deletion of DR6 might confer a generalized resistance of MNs to death-inducing agents. Accordingly, we set out to examine MN susceptibility to domoic acid and Fas activation^47^, two compounds known to cause MN degeneration by mechanisms distinct from those involved in astrocyte-induced MN death^20^. For this experiment, we went back to our primary MN culture system and, by following the protocol of Raoul and collaborators^47^, we demonstrated that both domoic acid and agonistic anti-Fas antibody killed WT and DR6^−/−^ MNs to a comparable degree (Fig. 6c), indicating that DR6 deficient MNs are not resistant to any insults. Thus, our findings support the notion that DR6—expressed at the surface of MNs—not only contribute to the demise of these cells in response to ALS astrocytes, but may also modulate MN vulnerability to this astrocyte-derived insult.

## Discussion

Molecular interactions between distinct cellular niches have emerged as a key determinant in an increasing number of pathophysiologic phenotypes. Yet, their systematic elucidation is lagging and still represents an unmet challenge in molecular biology. We have introduced SEARCHIN, as an analytic pipeline supporting the integration of diverse regulatory and proteomic evidence sources to prioritize the ligand–receptor interactions that most likely mediate these pathophysiologic phenotypes. Critically, our bioinformatics approach depends on the availability of regulatory networks, which can be inferred from gene expression data, on gene expression signatures that recapitulate the effect of the interaction, and on the availability of proteomics data identifying candidate ligands. As a result, SEARCHIN should be generalizable to a variety of additional contexts. Furthermore, recent results from the application of network-based approaches, such as metaVIPER to single cells, suggest that SEARCHIN may be effectively applied to elucidate mediators of cell-to-cell interactions at the single cell level^48^. However, analytical results are limited by the existing repertoire of available PPIs, in this case based on the PrePPI algorithm, which, albeit constantly expanding, is still not fully comprehensive.

Herein, we sought to apply our proposed integrative approaches to a cell-to-cell interaction setting provided by a MN death phenotype induced, non-cell autonomously, by neighboring human sALS and mouse mutSOD1 astrocytes^8–19^. As a preamble to our computational investigations, we performed a series of experiments aimed at better defining the biological framework of our selected cell-to-cell disease model. We began by clarifying whether MN degeneration is due to a lack of beneficial property or to a gain of rather toxic properties by ALS astrocytes. Relevant to this question is the work of Tyzack, et al.^49^ showing that the neuroprotective EphB1-ephrin-B1 cross-talk between MNs and astrocytes is disrupted in human ES-derived astrocytes and mutSOD1 mouse models of ALS. Although this study suggests that part of the non-cell autonomous arm of ALS pathogenesis is related to a loss of function, our present data argue that mouse mutSOD1 and human sALS astrocytes kill MNs by a gain of toxic function. Indeed, through a combination of biochemical methods, we provide evidence that mouse mutSOD1 astrocytes release a soluble toxic protein with a molecular mass between 5–30 kDa (Fig. 1b). This fact allowed us to rule out small molecules (≤1kDa) including oxygen and nitrogen reactive species and neurotoxic amino acids such as glutamate, which have often been proposed as causal in the ALS-related MN degeneration^50^ as well as lipids and prostanoids, since chloroform extraction fails to alter mutSOD1 astrocyte-induced MN death (Fig. 1c). In addition, using immunodepletion and supplementation experiments, we also excluded SOD1 itself, which has been reported as causing MN degeneration in vitro^13,23^.

Given the proteinaceous nature of the putative toxic factor, we then performed an unbiased proteomics analysis of the ACMs, and, first, examined these data for particular astrocyte inflammatory phenotypes such as A1 and A2 reactive signature^26^. Through a meta-analysis, we found marginal overlap between our proteomics and the genomic data of Liddelow, et al.^26^, allowing us to infer that mutSOD1 astrocytes are unlikely to kill MNs via an A1 astrocyte mechanism^26^. The corollary of this conclusion is that the A1 neurotoxic concept may be more context-specific than initially anticipated and raises the possibility that astrocytes contribute to neuron death by mechanisms that call for greater molecular nuances than the proposed A1/A2 dichotomy^26^.

All of the aforementioned investigations, not only confirmed the usual poor yield of candidate-based strategies aimed at elucidating the nature of the cell-to-cell communication, but also provided a solid rationale to try using an unbiased approach that combined ACM proteomics with MN functional genomics in a multistep bioinformatics process to address this challenging question. As such, we thought to utilize SEARCHIN as a proof-of-principle to show that meaningful biology can be identified via an integrative approach, rather than provide a comprehensive benchmark of the methodologies that include testing a variety of parameters, algorithms or assessing the impact of alternative evidence integration methodologies. Incidentally, in developing SEARCHIN, we realized that effective gold-standard databases for astrocyte-MN-specific interactions are lacking and, to our knowledge, there are no alternative methodologies to prioritize the cell-to-cell interactions that mediate a specific phenotype. As a result, any effort to compare the rankings generated by different methodologies would have had to rely on direct experimental validation, which is overly complex and time-consuming in this context. Within this framework, we found that SEARCHIN was able to distill a list of 19,370 potential interactions between 65 over-abundant proteins in the mutSOD1 ACM and 298 candidate surface and nuclear receptors in MNs to only three statistically significant ligand–receptor pairs (Nme1-Ptprn; Cdh2-Ryk; and App-Dr6), which represents a major implosion in the number of hypotheses that may need to be experimentally validated. Thus, although additional efforts to further validate the pipeline are still ongoing, it should be clear that SEARCHIN addresses an important, yet poorly addressed problem in molecular biology.

Prior to discussing our experiment validation, potential users must be aware that for this study, we have used well-validated algorithms like VIPER, CINDy, and PrePPI to test a methodology to generate reasonable and testable hypotheses for experimental validation. Yet, we do not assert that our proposed approach is the sole or even the optimal strategy to gain insights into the nature of non-cell autonomous pathogenic mechanisms, but merely a demonstration that will encourage investigators to develop and benchmark suitable variants—or even different implementations—as well as to generate appropriate gold-standard sets for systematic validation. It is also important to emphasize that for each individual step, we used the default parameters and thresholds, based on extensive benchmarks performed in the original manuscripts in which the various algorithms were introduced^2,3,51,52^. For instance, since PrePPI uses a likelihood rather than *p* value-based model, *p* = 0.5 represents the natural threshold at which an interaction is more likely to be true than false. In the corresponding manuscript^51^, we showed that >80% of the predictions made by PrePPI using this analysis were confirmed experimentally. In future studies, it will thus be interesting to determine whether different choice of parameters might produce even deeper biological insights into the cell-to-cell communication in models of human diseases, a possibility that cannot be assessed at this stage, owing to experimental complexity, lack of alternative methodologies, and lack of effective gold-standard datasets. At last, it should be remembered that we used an evidence integration approach, as our ultimate goal was to achieve a global score that represents the balanced integration of all the available evidences, rather than being dominated by a specific evidence source (e.g., PrePPI or VIPER). For this same reason, we favored a rank-based integration method rather than a *p* value-based one, which may bias some of the algorithms. As a result, in our study—as also typically done when using evidence integration approaches based on multiple weak clues—it is appropriate to use more relaxed statistical threshold for each individual evidence source contributing to the ultimate outcome.

As mentioned above, of the three significant ligand–receptor pairs identified by SEARCHIN, we began our experimental validation by studying the role of APP and DR6 in our ALS models, as APP–DR6 interaction has been previously validated^32–34^ and proposed in a few neurobiology-relevant phenotypes. For instance, APP-DR6 emerged as instrumental to axonal pruning during development^32,34^ and both DR6 and APP are critical for axonal plasticity in adult mice^53,54^. In addition, it was reported that APP-DR6 has a role in the pathophysiology of Alzheimer’s disease^32^, a view that could not be confirmed^33^. In contrast, for the two other statistically significant ligand–receptor pairs identified by SEARCHIN, we found no experimentally proven interaction between Nme1 and Ptprn while the interaction between Cdh2 and Ryk has been confirmed only by affinity capture-mass spectometry^55^. Yet, all of these four proteins have neurobiological activities relevant to brain development, axon guidance, synapse adhesion as well as pathological situations such as brain tumor, traumatic brain injury, and stroke^56–59^, which deserve further investigations in the context of neurodegeneration.

In support of the role of APP-DR6 in ALS, we found that DR6 is highly expressed in MNs (Fig. 5a) and that its ablation in these neurons was protective against the toxicity of both mouse mutSOD1 and human sALS astrocytes (Fig. 4), confirming that the identified non-cell autonomous toxic mechanism is not specific to mutSOD1 or mice. Furthermore, the importance of DR6 in MN degeneration was not restricted to our in vitro models as its reduction by viral gene silencing attenuated MN loss in Tg mutSOD1 mice (Fig. 5b, c). We also provide evidence that DR6 expression in MNs parallels that of MMP9 (Fig. 6a, b). As MMP9 is expressed in selectively vulnerable MNs^46^, our findings led us to posit that MNs with the highest levels of DR6 may be more prone to degeneration. However, more investigations are needed to elucidate the mechanism and pathogenic significance of the DR6/MMP9 correlation.

As for our validations of APP, our results suggest that MN toxicity involves an astrocyte-derived protein whose sequence is within the ectodomain of APP upstream to the β-secretase major cleavage site^60^. Relevant to this interpretation are the demonstrations that both ablation of APP^61^ and antibody inhibition of β-secretase^62^ mitigate the ALS-like phenotype in Tg mutSOD1 mice. Of note, unlike Rabinovich-Toidman, et al.^62^, we did not find, as reported in the results section, that reduction of β-secretase function using viral gene therapy altered SOD1 expression nor did we find, that silencing mutSOD1 altered APP expression.

Our idea that astrocyte toxicity is mediated by a soluble fragment of APP is also consistent with previous crystallography studies^63^ and cell-based binding assays^34,63^ showing that a fragment of sAPP-β can bind to DR6. Aside from APP, our results also raise the possibility for a role played by APLP1 in mutSOD1 astrocyte-induced toxicity. Indeed, we found that silencing APLP1, but not APLP2, in astrocytes consistently phenocopied the knockdown of APP (Supplementary Fig. 3). This finding suggests that both APP and APLP1 might function in the same molecular pathway that contribute to the death of MNs in response to mutSOD1 astrocytes.

In light of our results, we propose that APP-DR6 contribute to neurodegeneration via a β-secretase-dependent non-cell autonomous scenario, which is in contrast with the β-secretase-independent cell autonomous scenario put forward in the axonal homeostatic situations^32,34,53,54^. Given the role of DR6 in axonal pruning during development, surprisingly but importantly, DR6 silencing in our in vivo model of ALS protected against the loss of MN cell bodies but was less effective in mitigating motor axon damage (Fig. 5). Thus, our findings are critical since they argue for a distinct role of APP–DR6 in pathologic vs. normal neurobiological situations. Of note, dissociation between cell body and axon protection in Tg mutSOD1 mice is reminiscent of that reported for *Bax* deletion in these animals^64^, raising the possibility that APP/DR6 engage a molecular pathway of MN death that involves Bax, which would be consistent with our in vitro data^9,20^. In summary, the β-secretase-mediated role played by the APP/DR6 interaction in ALS is both novel and based on a solid foundation of prior results. Moreover, our demonstration that targeting DR6 attenuates MN death opens a unique opportunity to test therapeutic strategies aimed at blocking/slowing nerve terminal degeneration, a combination of effects that is required to ultimately extend muscle function and thus greatly improve the quality of life for patients with ALS.

In conclusion, our presented combination of computational multi-modal analysis followed by experimental validation provides an effective proof-of-principle for a generalizable methodology directed at elucidating cell-to-cell communication mechanisms. Indeed—based on its ability to reduce the number of candidate hypotheses to a manageable number—use of proteomics data to prioritize candidates from one compartment may be effectively replaced by their gene expression, wherever possible. Although this will increase the number of candidate hypotheses, it will also allow direct application to a number of studies for which data are already available.

## Methods

### Humans and animals

All of the studies with human postmortem tissues were approved by Columbia IRB Committee protocol AAAA8153. Procedures related to in vitro experimentation with cells produced or derived from mice B6SJL were approved by Columbia IACUC protocols AAAD8107, AAAL2502, and AAAN2050. All animal related procedures and euthanasia were approved by Columbia University’s Institutional Animal Care and Usage Committee. The entire mouse study was performed using B6.Cg-Tg(SOD1*G93A)1Gur/J mice (Cat# 004435; Jackson Laboratory) and NTg controls B6SJLF1/J mice (Cat# 100012; Jackson Laboratory).

### Primary astrocyte cultures

AMLs from mice were prepared and genotyped as described^20^. In brief, postnatal day 1 pups from WT or mutSOD1 with B6SJL background were killed using approved IACUC protocol. The cortices were dissected out from the brain after carefully removing the meninges and deeper cortical structures. The cortex from each animal was dissociated by passing through 18 G needle (305196, BD PrecisionGlide) and plated on 75-cm^2^ flasks containing Dulbecco’s Modified Eagle Medium (DMEM, 12430-054, Gibco), 10% fetal bovine serum (FBS, A31604-02, Gibco) and 1% Pennicillin/Streptomycin (15140-122, Gibco). After 2 weeks, the cells were agitated (200 rpm, 6 h) to remove residual microgila. The cells were trypsinized and plated on 75-cm^2^ flask in astrocyte media described at a density of 100,000 cells/mL and incubated for 7 days. The ACMs were prepared from astrocyte cultures of different genotypes, collected, frozen in several aliquots, and then supplemented the day of the experiments^20^.

Frozen aliquots from three individual human non-neurological disease control and three sALS astrocytes obtained from Re, et al.^9^ were used for these investigations. In brief, fresh autopsied tissues were received from Columbia Medical Center Morgue or National Disease Research Interchange (NDRI) in (1:1) media containing DMEM (12430, Gibco) and F10 (11550, Gibco) and 1% gentamicin (G1272, Sigma-Aldrich). After removal of blood vessels and meninges, the tissue was cut into smaller sections. This crude suspension was incubated in Hanks' Balanced Salt Solution (14025, Thermofisher Scientific) containing 0.25% typsin (2500056, Thermofisher Scientific), 0.2 mg/mL ethylenediaminetetraacetic acid (EDTA) (ED2SS, Sigma-Aldrich), 1 mg/mL glucose (G8769, Sigma-Aldrich) and 0.1 mg/mL bovine pancreatic DNaseI (LK003172, Worthington) for 20 min at 37 °C. The cell suspension was then triturated with DMEM/F10 media containing 10% FBS (SH30070.02, Fisher Scientific) and 1% penicilliln/streptomycin (15140-163, Thermofisher Scientific). These cultures were passed through 0.22 μm filter and then plated on 75-cm^2^ flasks for 2 h at 37 °C to allow monocytes and macrophages to adhere. The supernatant from these cultures are transferred to another 75-cm^2^ flask coated with Poly-l-Lysine (15 μg/mL, Sigma-Aldrich) and media was changed after 48 h. The cells were allowed to grow until confluence and then dissociated and plated for experiments or frozed for additional experiments. As described previously^9^ these human cell cultures expressed the astrocytic markers GFAP, vimentin and CD44 while they were immunonegative for microglial protein ionized calcium binding adaptor protein-1, oligodendritic marker 2’,3’-Cyclic nucleotide 3’-phosphodiesterase, and neuronal marker microtubule-associated protein 2.

### Primary MN cultures

Primary SC neuronal cultures were prepared following the protocol described in ref. ^20^ using embryos from Tg mice expressing GFP driven by the mouse HB9 promoter^65^, and mice homozygote for a null mutation in DR6 (Stock # 2994, B6.129X.tm1Sjk/J from the Jackson Labs., MI, USA) kindly gifted by Genentech or for a null muation in p75^NTR^ (Stock #2213, B6.129S4-Ngfrtm1Jae/J from the Jackson Labs. MI, USA). In brief, SCs dissected from embryos were incubated using 0.025% trypsin (15090-046, Gibco) and 1 mg/mL DNase (LK003172, Worthington) for 10 mins at 37 °C. The cells were then triturated and gently placed on 4% bovine serum albumin (BSA, A9418, Gibco) cushion and centrifuged for 300 × *g* for 5 min. The pellet was finally resuspended in Neurobasal medium (ME120079L2; Gibco), 2% B27 supplement (17504-044; Gibco), 2% HS (26050070; Gibco), 0.5 mM l-glutamine (25030-081; Gibco), 25 μM β-mercaptoethanol (ES-007-E; Millipore), and 1% P/S (15140-122; Gibco), supplemented with 1 ng/ml brain-derived neurotrophic factor (BDNF) (450-02-10UG; PeproTech), 0.5 ng/ml glial cell line-derived neurotrophic factor (GDNF) (450-10-10UG; PeproTech), 10 ng/ml ciliary neurotrophic factor (CNTF) (450-13-20UG; PeproTech), counted and plated at desired cell density as described below.

### Mouse ES-derived neuron cultures

ES cells, derived from Tg mice expressing GFP driven by the mouse HB9 promoter^65^ were differentiated into MNs using well-established differentiation protocol^20^. In short, mouse ES cell line Hb9::eGFP (a gift from Dr. Hynek Wicheterle) were plated on gelatinized 25 cm^2^ flask in DMEM media (SLM220B, Millipore Sigma) containing 15% ES-FBS (ES-009-B, Millipore Sigma), 1% penicillin/streptomycin (15140-122, Gibco), 1% glutamine (25030-081, Gibco), 1% non-essential amino acids (TMS-001-C, Millipore Sigma), 1% nucleosides (ES-008-D, Millipore Sigma), 1% β-mercaptoethanol (ES-007-E, Millipore Sigma), 1% Sodium pyruvate (S8636, Sigma-Aldrich) and 0.1% LIF (ESG1107, Millipore Sigma). After 48 h, the cells were trypsinized and plated at 1–2 million cells in 10 cm^2^ culture dish (353003, BD Falcon) containing αDFNK media containing 1:1 ratio of Advanced DMEM (12634, Gibco) and Neurobasal A (12349015, Thermofisher Scientific), 10% Knock Out Serum Replacement (ES-007-E, Millipore Sigma), 1% penicillin/streptomycin (15140-122, Gibco), 1% glutamine (25030-081, Gibco) and 1% β-mercaptoethanol (ES-007-E, Millipore Sigma for allowing non ES cells to adhere. The supernatant containing embryoid bodies was removed and transferred to Ultra low attachment dish (326, Corning). Next day, differentiating factors 1 µM retinoic acid (R2625, Sigma-Aldrich) and 0.25 µM Smoothened Agonist (566660, Calbiochem) were added for 72 h. Differentiated EBs were then transferred to fresh media for 24 h and dissociated using 0.5% trypsin EDTA (15400-054, Gibco) for 5 min at 37 °C. After halting the trypsinization with Horse serum (26050070, Thermofisher Scientific) the EBs were gently triturated using 1 mL tip and transferred to 4% BSA cushion, spun at 300 × *g* for 5 mins. The pellet was resuspended in ES media (similar to Primary Neuron media recipe described above except 10 ng/mL BDNF and 10 ng/mL GDNF). The cells were plated at density of 3000 GFP+ve cells/well in a 96-well plate.

### Gene silencing in astrocytes and MNs

#### Primary astrocytes

All shRNAs and EVs used in this study were from Sigma MISSION. For astrocytes, a suspension of 100,000 cells/mL was treated with 8 μg/mL of hexadimethrine bromide (107689; Sigma-Aldrich, St. Louis, MO), exposed to lentiviral particles at an MOI of 15 or 50, centrifuged (800 × *g*, 30 min, room temperature), and plated (20,000 cells/well on a 96-well plate or 80,000 cells/well in 24-well plates). After 48 h, cells were selected by adding 1 μg/ml puromycin (A11138); Invitrogen, Carlsbad, CA) for 4.5 days and placed in normal medium for 7 days before being co-cultured with MNs. For primary and ES-derived MN transduction, hexadimethrine bromide and puromycin were omitted.

#### Primary neuronal cultures and co-cultures

All shRNAs and EVs used in this study were constructed in pLKO.1-puro plasmids (Sigma MISSION®). This plasmid contains a puromycin resistance cassette inserted behind a human phosphoglycerate kinase eukaryotic promoter (http://www.sigmaaldrich.com/life-science/functional-genomics-and-rnai/shrna/library-information/vector-map.html#pLKO). For human SOD1 silencing, the clones TRCN0000009869, TRCN0000018344, TRCN0000039808, and TRCN0000039812 were used. For APP silencing, the clone TRCN0000054877 was used. For APLP1 silencing, clone TRCN0000106646 and for APLP2 silencing clone TRCN0000080052 were used. As controls, SHC001V (empty vector, EV) was used in all of the experiments, and SHC002H, a pLKO.1-puro non-mammalian shRNA control or SHC012V, a pLKO.1-puro-CMV-TagRFP™ positive control was used back to back with EV in several of these. For co-culture experiments, astrocytes were processed as described above and neurons were added on top of the astrocytes (added cell density: 12,000 in 96-well plates and 35,000 mouse primary MNs for 24-well plates). In each experiment, some of the wells did not receive MNs so as to serve as control wells to verify the efficiency of silencing by mRNA or protein extraction.

### Quantitative real-time PCR

RNA samples were obtained from 24- or 96-well cell extraction (AM1931, RNAqueous Micro-Kit, Invitrogen, Carlsbad, CA). RNA (50–100 ng) was first reverse transcribed to cDNA (500 ng/μL) using Superscript II three-step reverse transcription PCR (11904-018, Invitrogen, Carlsbad, CA). A two-step qRT-PCR was carried out with Realplex 4 Mastercycler PCR System (Eppendorf, NY) using FAM-conjugated gene specific TaqMan® primers (Applied Biosystems, Invitrogen, Carlsbad, CA) in triplicate for each sample. Primers for GAPDH (Applied Biosystems, Invitrogen, Carlsbad, CA) were used as housekeeping control gene to normalize all target gene qRT-PCR results. qRT-PCR results were quantified by the Ct method following published recommendations (Schmittgen & Livak, 2008). Target genes for which Ct ≥35 were considered as unamplified and were thus referred to as non-detected.

### Pharmacological and recombinant treatments

Treatment with SOD1 and E1/E2 recombinants, secretase inhibitors, domoic acid and agonistic anti-Fas antibody was done similar to our previously published article^9^. SOD1 recombinants (gift from J.-P. Julien) were added at the indicated concentarions and  E1 and E2 recombinant protein were added to ES-derived MNs at 3 µm on DIV2 monitored daily until DIV 7. β-secretase inhibitor AZ29 (gift from Genentech), α-secretase inhibitor TAPI (579051, Millipore Sigma), γ-secretase inhibitor (565771, Millipore Sigma), domoic acid (0269, Tocris), agonistic anti-Fas antibody (AB16982, Millipore Sigma), and anti-P75NTR neutrilizing antibody (Millipore clone ME20.4, cat number 05-446) were added at DIV 1 and DIV4. Cell survival and death were evaluated at the end of the DIV 7 by counting GFP-positive MNs.

### Immunodepletion experiments

For immunodepletion experiments, anti-SOD1 antibody (Abcam 13498) was first incubated with magnetic agarose beads for 15 min at room temperature on rotation. After removing all of the unbound supernatant, beads were resupsended in antibody binding and washing buffer. Next, the beads were incubated with ACM from NTg and mutSOD1 astrocytes for 15 min at room temperature while rotating. After incubation with ACM, the antibody-bound beads were then separated using magnets to pull down the bound antibody complexes. The cleared CM was then applied to WT primary MNs and GFP+MNs remaining after DIV 7 were then counted as described above.

### Immunocytochemistry

Cells were processed for immunocytochemistry as previously described^20^. Primary antibodies used here were: rabbit polyclonal anti-GFP (1:1000; A11122; Molecular Probes, Eugene, OR), rabbit polyclonal anti-GFP (1:1000; A11120, Molecular Probes, Eugene, OR), chicken polyclonal anti-MAP-2 (1:5000; ab5392; Abcam, Cambridge, England), mouse anti-SMI-32 for non-phosphorylated neurofilament heavy chain (1:500; SMI-32R; Covance, Princeton, NJ), mouse monoclonal anti-vimentin (1:500; V2258 Sigma-Aldrich, St. Louis, MO), and rabbit monoclonal anti-GFAP (1:500; a gift from Dr. James Goldman at Columbia University) antibodies. After overnight incubation (4 °C), coverslips were washed twice with Dulbecco’s phosphate-buffered saline (DPBS) (14190; Invitrogen, Carlsbad, CA) and incubated (1 h, room temperature) with the appropriate fluorescent-conjugated secondary antibodies (1:400; Molecular Probes, Eugene, OR). Coverslips were then washed and incubated with DAPI (D9564, Sigma-Aldrich, St. Louis, MO) at 0.1 μg/mL in phosphate-buffered saline (PBS) at room temperature for 5 min. After two DPBS washes, coverslips were mounted in Dako Cytomation Fluorescent mounting medium (S302380-2; Dako, Glostrup, Denmark).

### Neuromuscular junction analysis

Mice at P90 and P125 were killed as per our IACUC approved protocol. The mice were perfused with 4% paraformaldehyde and the tibialis anterior muscle was dissected out. The muscle was stored in PBS overnight and then transferred to 30% sucrose for 24 h. After washing the muscle with PBS, the muscle was frozen on dry ice using clear OCT compound (Fisher Healthcare #4585). The blocks of muscle were then cryo-sectioned longitudinally into 20 µm slide-mounted sections. For staining, the slides were washed with PBS for 5 min and then blocked with 4% BSA+1% Triton-X 100 in PBS for 1 h at room temperature. The slides were then treated with Tetramethylrhodamine α-bungarotoxin (1:200; ThermoFisher Scientific T1175) and rabbit anti-vesicular acetylcholine transporter antibody (1:16,000; Covance CV1721) in 0.4% BSA+0.1% Triton-X 100 overnight at 4 °C in a humid chamber. The slides were washed and treated with donkey anti-rabbit Alexa 488 (1:400) and DAPI for 1.5 h at room temperature in a humid chamber. Finally, the slides were washed three times with PBS containing 0.2% Triton-X 100 and mounted with coverslips then and imaged on a Leica confocal microscope. At least 50 NMJs were counted for each muscle.

### Immunohistochemistry

Mice were perfused using 4% paraformaldehyde. SC from P125 mice was dissected out post-fixed in 4% PFA overnight. SC was then transferred to 10% sucrose for at least 48 h at 4 °C. The SC was embedded in 7.5% gelatin and 10% sucrose at 37 °C and frozen on dry ice. 15 µm sections were cut on a cryostat. The sections were blocked with 10% Donkey serum and 0.2% Triton-X 100 in PBS. Primary antibody donkey anti-ChAT (1:125; Millipore AB144P) was added to the sections overnight in a humid chamber. The sections were washed with 0.2% Triton-X 100 in PBS followed by secondary antibody anti-goat Alexa 594 (1:250; Life Technologies A-11058) and 4′,6-diamidino-2-phenylindole (DAPI) for 3 h at room temperature. The slides were cover-slipped then mounted and imaged on a Leica Confocal microscope to count surviving MN numbers. ChAT-positive neuron on each hemi-section were counted.

### RNAscope assay

L4/L5 section of SC tissue from WT mice was dissected out and frozen on dry ice in aluminum foil with minimum delay. The frozen tissue was embedded in OCT right away and stored in −80 °C for 24 h. Then, 20 μm thick sections from L4/L5 were cut with a cryostat and mounted on Frost-Free glass slides. The sections were stored at −20 °C for at least 24 h before processing them for RNAscope assay. RNAscope Fluorescent Multiplex Reagent kit (320513 for fresh frozen tissue and 320293 for Multiplex Reagent kit, Advanced Cell Diagnostics) was used for detecting ChAT, MMP9 and DR6 mRNA. The assay was performed exactly as per manufacturer’s recommended protocol. DAPI was used to label cell nuclei. The slides were allowed to dry overnight and imaged using Leica Confocal microscope.

### Behavior tests

Loaded grid test: The loaded grid test described by Barneoud et al.^66^ was used to assess limb muscle strength. One week before starting the test, the mice were pre-trained to hold the grid. Mice were weighed every time the assay was performed. Starting at P70, grids of increasing weight (10, 20, 30, and 40 g in ascending order) were given to the mice to hold for 30 s. Three trials were given for each weight with 15 s rest between each similar weight. Before moving to a different weight, 30 s rest period was given. The mice were suspended by their tails and given the weighted grid between their forelimbs and hindlimbs for 30 s. The best of three trials was used for statistics. Following equation was used to compute final score for each mice.

$$Loaded\,grid\,score = \frac{{10\left( {t1} \right) + 20\left( {t2} \right) + 30\left( {t3} \right) + 40(t4)}}{{weight\,of\,mouse}}$$

where *t*_1_, *t*_2_, *t*_3_, and *t*_4_ represent the seconds the mouse held on to weights 10, 20, 30, and 40 g, respectively.

### Bioinformatics analysis

The SEARCHIN pipeline implements a systematic and unbiased genome-wide approach to the identification of cross-compartment ligand–receptor pairs responsible for transducing signals from one cellular compartment to activate or inhibit MR proteins in the other. Specifically, the proof-of-concept SEARCHIN implementation described in this manuscript is aimed at identifying the ligand–receptor interactions that mediate transduction of death signals that determine MN demise when exposed to culture media from mutSOD1 astrocytes. The full source code used to run the experiment discussed in the manuscript, and a comprehensive tutorial on how to use the methodology and how the datasets were built. The source code is available under the GNU GPLv3 license at the following URL: http://www.github.com/Califano-lab/SEARCHIN. The tutorial is also available at the same link. The pipeline is conceptually summarized as follows.

#### Ligands binding partner predictions

To prioritize ligand–receptor binding interactions, we leveraged a large collection of PPIs inferred by the PrePPI algorithm^4,51^. PrePPI integrates 3D protein structural information as well as other sources of information, such as Gene Ontology and experimentally determined interactions. The current version of the PrePPI database contains 1,545,710 pairs of interacting proteins that are based on the human proteome, considered as high confidence predictions (probability > 0.5). To generate a null model, we used the full collection of potential PPI pairs that PrePPI may infer (i.e., 203,931,823 based on 20,259 proteins). Specifically, we generated a data driven empirical null model for each candidate interaction, by integrating the PrePPI scores from (a) binding partners of a ligand identified by MS, and (b) proteins predicted to interact with a differentially active receptor inferred by VIPER. In brief, for each possible interaction between two proteins P_1_ and P_2_, we built an empirical null model using the collection of all the PrePPI scores between P_1_ and all its putative binding partners present in PrePPI database (i.e., 20259 protein coding genes); the same was done for P_2_. The *p* value was generated with the empirical *p* value method that calculates *p* values from a set of observed test statistics (see ref. ^67^). This approach enabled helped us correct any bias owing to the number and specificity of individual PPIs, thus providing a more robust biological rationale for the empirical null model. We used an open source implementation of a graph database (i.e., the Neo4J Open Source Community Edition 3.2.5, http://neo4j.org/) to build a PPIs interactome from PrePPI inferences and the Cypher Query Language to access it programmatically through Python 3.6 scripts. An empirical *p* value was computed using the package *q* value-2.8.0 in the R environment version 3.4.1. This step of the SEARCHIN pipeline generated a list of 1478 candidate PPIs (Supplementary Table 2).

To combine MS data (i.e., proteins from rat ACM) with PrePPI (i.e., human proteome), we used the programmatically accessible web services provided through BioMart^68^ for biological identifier conversion (e.g., to translate orthologous genes between organisms or gene identifiers and their protein products).

#### Membrane receptors inferred activity

We used the ARACNe algorithm^1^ to reverse-engineer a membrane protein-specific regulatory network from 437 mouse brain expression profiles (GSE10415). As regulators with fewer than 25 targets were shown to reduce inference accuracy, as comprehensively benchmarked in ref. ^2^, only plasma membrane proteins, annotated in the Gene Ontology^69^ database as “signal transduction activity” (GO:0005886 and GO:0007165), with ≥25 target genes (among 16,589 possible ones) were included in the network, for a total of 873 murine receptor and 376,672 transcriptional interactions. The plasma membrane interactome is available through FigShare at the following permanent URL: 10.6084/m9.figshare.6387671. The VIPER algorithm^2^ was used to infer changes in plasma membrane signaling protein activity based on the mutSOD1 gene expression signature and mouse brain plasma membrane regulatory model. Because the inferred activity is based on the transcriptional programs induced by the treatment, the algorithm will identify the specific receptors as well as other regulatory proteins associated with similar transcriptional programs. We used the list of differentially active receptors, based on the rank-ordered *p* values (from the most to least significant) as another source of evidence to further prioritize the candidate paracrine interactions. The list of candidate receptors is shown in Supplementary Table 3.

#### NF-κB1 modulators inference

The CINDy algorithm^3^ was used to predict the most likely modulators of NF-κB1 activity among the membrane receptor proteins. We used gene expression profiles from 437 mouse brain samples (GSE10415) as input to CINDy. The null model was generated using the number of triplets (i.e., receptor-TF-target) identified by the algorithm for NF-κB1. Specifically, only the triplets that had a Conditional Mutual Information (CMI) greater than or equal to the average CMI of the data set were considered. The list of candidate modulators is shown in Supplementary Table 4.

#### Scores integration

To combine the scores from the different sources of evidence, we used the Robust Rank Aggregation method for meta-analysis^27^. From the R package RobustRankAggreg version 1.1, we used the function “aggregateRanks” with method “RRA”. The final list of ranked ligand–receptor pairs is shown in Table 1. For validation, we selected the first pair, i.e., APP/TNFRSF21, with the highest “experimental score” as reported in PrePPI DB, whose receptor was also shown to be differentially activated.

#### SEARCHIN code and implementation

Although the present manuscript provides the outline of the algorithm, its full source code is available at the following URL: https://github.com/califano-lab/SEARCHIN.

#### Treatment of ACM

ACM were passed through Q or S columns (Pierce Strong Anion Exchange Spin column, Thermo Scientific #90011; Cation Exchange Spin column, Thermo Scientific #90009). Sample flow-through from the column after passing the sample was discarded. Molecules bound to the column were eluted by increasing concentrations of NaCl (0.5, 1, and 2 M respectively). The eluate was desalted using spin columns (Amicon Ultra-15 Centrifugal Filter Unit with Ultracel-3 membrane, Millipore, Cat. # UFC900396). The elute was brought up to original volume and supplemented with HS, B27, P/S, l-glutamine, and β-mercaptoethanol and then applied to primary SC MN cultures.

In another set of experiments, ACM media from NTg and mutSOD1 were passed through cutoff filters of 5, 10, 30, 50, and 100 kDa to separate potential toxic moieties based on molecular weight. The components of the ACM retained on the filters were then resuspended in fresh MN culture media before being applied to primary SC MN cultures to examine their effect on viability.

For heat inactivation, ACM from NTg and mutSOD1 astrocytes were incubated in a water bath at 95 °C for 15 or 30 min, cooled down to 37 °C and then resupplemented with horse serum (2%) and trophic factors as described in ref. ^20^ before being applied on mouse primary SC MN cultures. To eliminate proteins, ACM from NTg and mutSOD1 were first adjusted to a pH 2.5 with 1 N HCl and then incubated with pepsin at 37 °C for 2 hours. At the end of the incubation the pH was readjusted to 7.4 with 1 N NaOH and ACM were re-supplemented as described above. Control incubations identical to those above were performed except that pH was not adjusted and ACM were kept at room temperature to prevent pepsin activity. For charcoal treatment, ACM were incubated for 5 min at RT with 50 mg/mL charcoal (Norit: activated and neutralized; Sigma 97876), then centrifuged at 11,500 × *g* for 3 min. The supernatants were then filtered and re-supplemented as described above before treatment. For lipid depletion, ACM were mixed with chloroform (v/v), vortexed for 30 s and centrifuged for 5 min at 750 × *g*. The chloroform phase was then removed and this extraction was repeated twice. The aqueous phase was then poured in a petri dish and placed in an incubator at 45 °C for 30 min to remove all traces of chloroform. Finally, the volumes were readjusted to the original volume of the aqueous phase with sterile water, filtered, and re-supplemented as described in ref. ^20^.

### Mass spectrometry

ACM derived from rat astrocytes was analyzed by Gel LC-MS/MS as described previously^24^. In brief, ACM were resolved on NuPAGE 10% Bis-Tris gels (Invitrogen, Carlsbad, CA). Proteins were visualized by Colloidal blue (Invitrogen, Carlsbad, CA). Uniform (2 mm) slices were cut into 1 × 1 mm cubes followed by in-gel digestion with trypsin. Tryptic digests were analyzed on hybrid LTQ-Orbitrap mass spectrometer (Thermofisher Scientific, San Jose, CA) coupled with a NanoLC pump (Eksigent Technologies, Livermore, CA). Peptide and protein identification and quantification were performed using MaxQuant. The raw intensities of peptides in MS1 were transformed to log2 values, which were then used to calculate the difference in relative levels of proteins between mutSOD1 and NTg.

### Cell counting and statistics

Results correspond to independent experiments and each experiment is the average of 3–6 coverslips or 96-wells per time point and per condition. For mouse ES-MN number with co-culture, MN number was assessed by automatic quantification using the Metamorph Imaging System software (Universal Imaging Corporation, Downingtown, PA). For primary cultures, MNs were counted manually using the Nikon Epifluorescent microscope. The investigator counting the MN numbers was always blind to the treatment conditions. Differences between means were analyzed by a two-tailed Student’s *t* test. Photoshop v19 was used for preparing figures. NIH Fiji ImageJ was used for measuring intensity for RNAscope analysis. Differences among means were analyzed by one- or two-way analysis of variance (ANOVA) with the different types of astrocytes, MNs or treatments as independent factors. When the ANOVA showed significant differences, pair-wise comparisons between means were tested using Sidak post hoc testing, unless stated otherwise. In all analyses, the null hypothesis was rejected at the 0.05 level. All statistical analyses were performed using SigmaPlot for Windows (version 12.0; Jandel Corp., San Rafael, CA) and software GraphPad Prism version 7 (San Diego, CA). Loaded grid test data were fitted by a non-linear iterative least squares method provided by the software GraphPad Prism. Then, the two different curves were compared by the extra sum-of-squares *F* test^70^. Survival statistics was performed by Kaplan–Meyer analysis also with GraphPad Prism. Whenever suitable, statistical results include 95% confidence interval and the Cohen’s *d* effect size.

### Reporting summary

Further information on research design is available in the Nature Research Reporting Summary linked to this article.

## Supplementary information

Supplementary Information

Reporting Summary

## Data Availability

The authors declare that all the main data supporting the findings of this study are available within the article and its Supplementary Information files. All the data sets that were used as input for the study and the code to reproduce the manuscript findings are available at the following citable link: 10.5281/zenodo.4037259^71^. The mass spectrometry proteomics data are available via ProteomeXchange at https://www.ebi.ac.uk/pride/ with the data set identifiers PXD021773.

## Source data

Source Data

## Code availability

The authors declare that the SEARCHIN source code and tutorial are available under the GNU GPLv3 license at the following URL: http://www.github.com/Califano-lab/SEARCHIN^71^.
