# Supplementary material for: Systematic elucidation of neuron-astrocyte interaction in models of amyotrophic lateral sclerosis using multi-modal integrated bioinformatics workflow
Source: Nat Commun. 2020 Nov 4;11:5579. doi: 10.1038/s41467-020-19177-y (PMC7642391; doi:10.1038/s41467-020-19177-y)
Supplement: Supplementary file 1 — Supplementary Information [file 41467_2020_19177_MOESM1_ESM.pdf]

Supplementary Information for:

Systematic elucidation of neuron-astrocyte interaction in models of amyotrophic lateral sclerosis using multi-modal integrated bioinformatics workflow

Mishra et al., Nature Communications

a

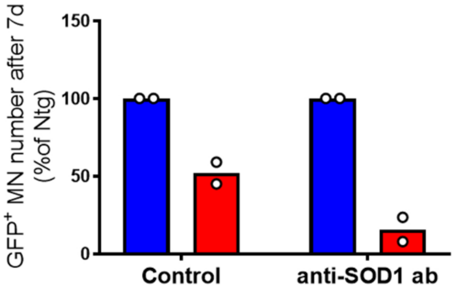

b

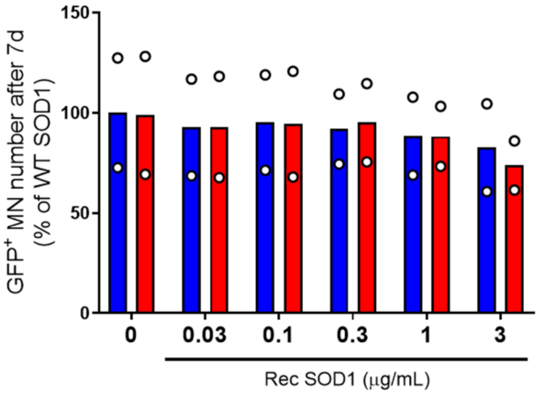

Supplementary Figure 1. Soluble SOD1 does not mediate MN death. **a** NTg ACM (blue) or mutSOD1 ACM (red) was incubated with no antibody or with an anti-SOD1 antibody (anti-SOD1 ab) for 15 min at room temperature. Immunodepletion of SOD1 from ACM was confirmed by immunoblot to be ~85% of non-immunodepleted ACM control. Immunodepleted ACM was then applied to ES-MNs expressing EGFP under the control of the MN specific Hb9 promoter. GFP+ neurons were counted at 7 DIV using Metamorph software after 5 days incubation. **b** Various concentrations of WT SOD1 (blue) or mutSOD1 (red) recombinant (Rec SOD1) were added to ES-MNs. GFP+ MNs were counted at 7 DIV using Metamorph after 5 days incubation. Data are means of two independent experiments. Source data provided as source data file.

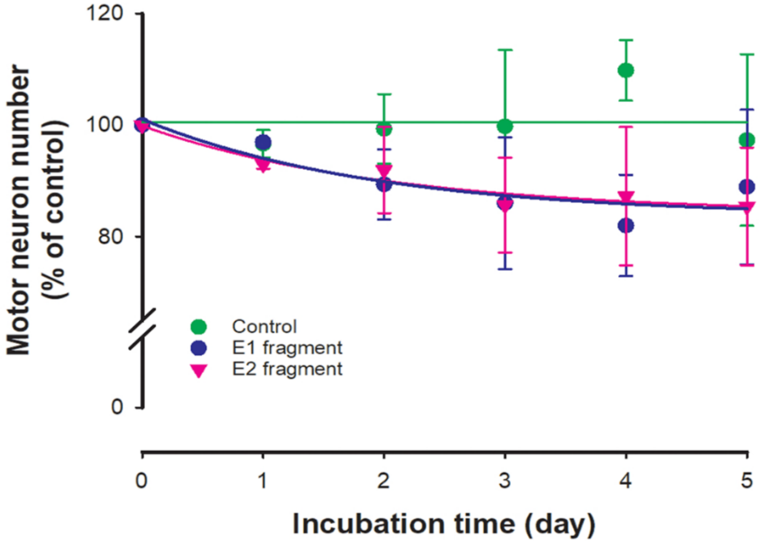

Supplementary Figure 2. E1 and E2 treatment leads to loss of ES-derived MNs. ES-MNs expressing EGFP under the control of the MN specific Hb9 promoter were either untreated or treated at 2 DIV with 3 μM of bovine serum albumin (BSA) (n=3), E1 recombinant (n=4) or E2 recombinant (n=3) for 5 days. The cells were imaged every 24 h and GFP+ neurons were quantified using Metamorph software. Curves were fitted with a 3 parameter exponential decay equation. Extra-sum-of-squares test conclude that E1 ($F_{(3,6)}$ = 103; p = 1.5 x10^{-5}) and (E2 $F_{(3,6)}$ = 61.8 ; p = 6.7 x 10^{-5}) are both significantly different from control. Data are means ± SEM of n independent experiments. Source data provided as source data file.

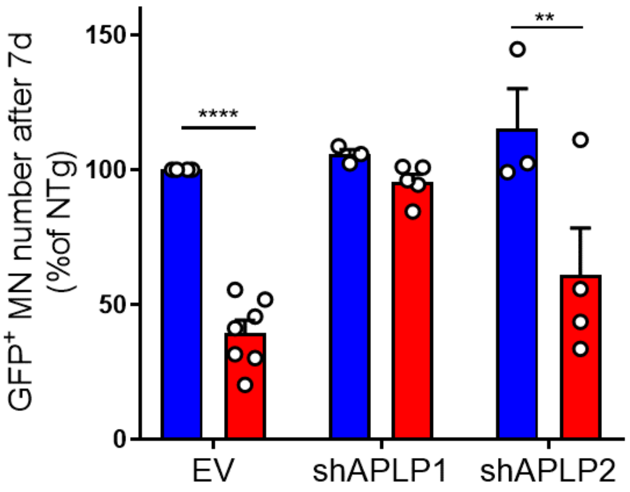

Supplementary Figure 3. APLP-1 and APLP-2 affect toxicity of astrocytes to a variable extent. NTg (blue) or mutSOD1 (red) astrocytes were infected with empty vector (EV) (NTg n=4, mutSOD1 n=7), with sh-APLP-1 (NTg n=3, mutSOD1 n=5) or with sh-APLP-2 (NTg n=3, mutSOD1 n=4) and were selected with puromycin for 4.5 days. Then, selection media were replaced with regular astrocyte media before transduced astrocytes were co-cultured with mouse ES-MNs expressing EGFP under the control of the MN specific Hb9 promoter. Viability was quantified 7 days after MN plating. ES-MNs were counted using Metamorph software. Data are means ± SEM of independent experiments (n) and were analyzed by two-way ANOVA (Interaction $F_{(2, 20)}$=4.997, P=0.0174) followed by Sidak's post-hoc test: ****$P \leq 0.0001$ EV NTg vs EV mutSOD1 AML (CI: 32.19% to 89.01%; d=7.29); **P=0.0017 shAPLP2 NTg vs shAPLP2 mutSOD1 (CI: 19.75% to 88.98%; d=2.11). Source data provided as source data file.

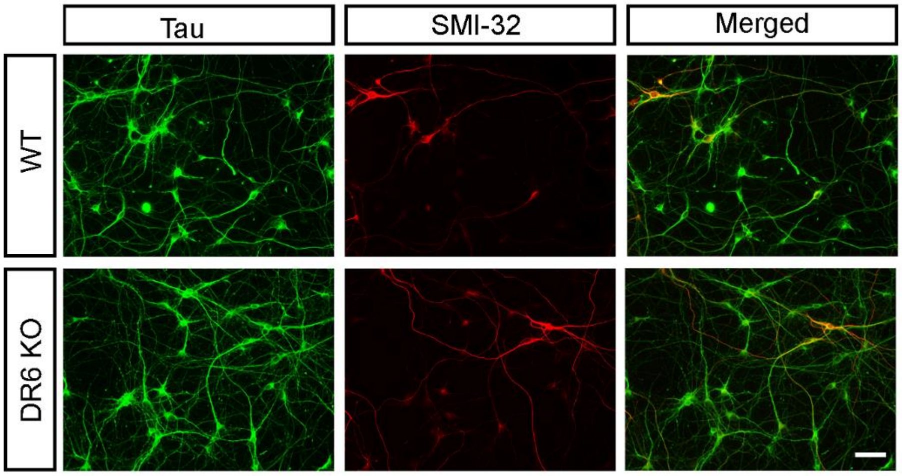

Supplementary Figure S4. DR6$^{-/-}$ neurons are morphologically similar to WT neurons. Representative image of WT or DR6$^{-/-}$ neurons of a single independent experiment immunostained with an anti-pan-neuronal marker Tau (green) and the anti-non-phosphorylated heavy chain neurofilament SMI-32 (red) antibodies. *Scale bar*= 50 µm.

a

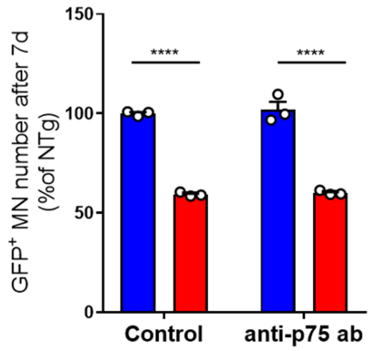

b

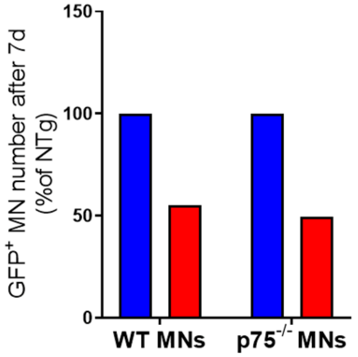

Supplementary Figure 5. The MN receptor p75NTR does not participates in mediating the mutSOD1 astrocyte-derived MN death signal. a NTg ACM (blue) or mutSOD1 (red) ACM was incubated with no antibody or neutralizing anti-p75NTR antibody (anti-p75 ab) for 15 min. ACM treated with the neutralizing antibody was then applied to mouse ES-MNs expressing EGFP under the control of the MN specific Hb9 promoter. GFP+ neurons were counted using Metamorph software after 7-day incubation. Data are means ± SEM of 3 independent experiments and were analyzed by two-way ANOVA revealing that anti-p75 ab did not mitigate the effect of mutSOD1 ACM on MNs (genotype main factor $F_{(1, 8)}$ = 414, P<0.0001) followed by Sidak's post-hoc test: ****P≤0.0001 Control NTg vs mutSOD1 (CI: 32.75% to 48.52%; d=66.07); ****P ≤ 0.0001 anti-p75 NTg vs anti-p75 mutSOD1 (CI: 34.04% to 49.78%; d=10.63). **b** NTg or mutSOD1 ACM was applied to primary MNs from WT or p75NTR knockout ($p75^{-/-}$) mice for 7 days. Data are means of a single independent experiment. Source data provided as source data file.

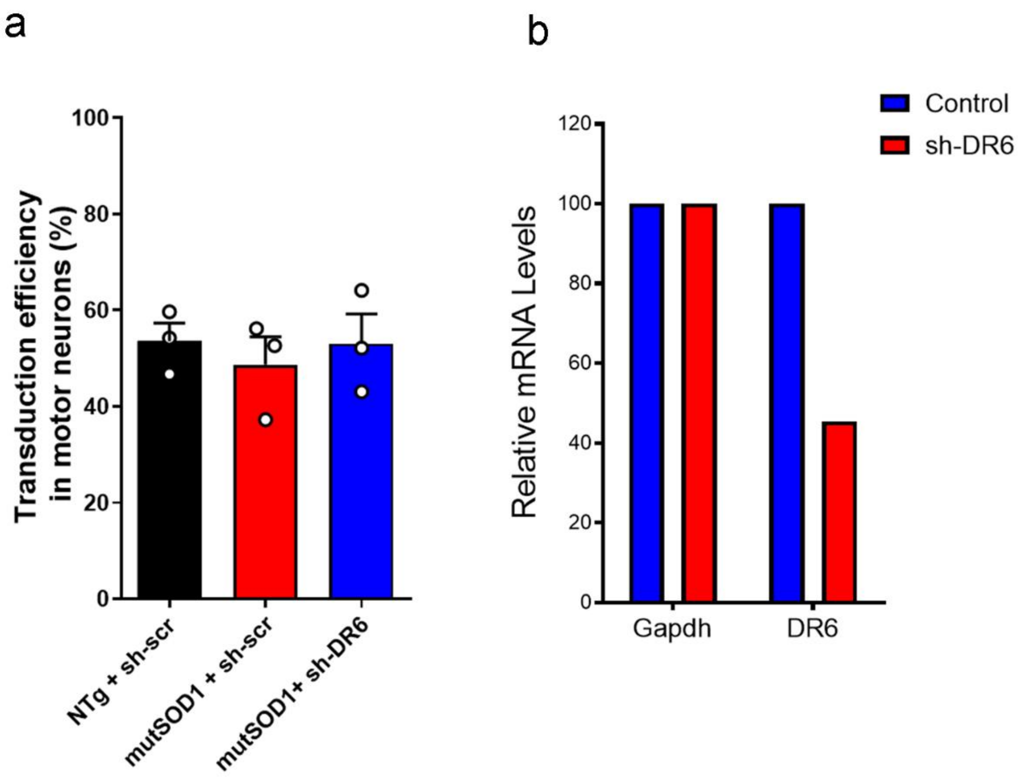

Supplementary Figure 6. Validation of transduction efficiency for AAV9-sh-DR6 in mouse model. a Transduction efficiency was assessed by counting the number of mouse ES-MNs expressing EGFP under the control of the MN specific Hb9 promoter that were also ChAT (MN marker) immunopositive. Lumbar spinal cord was perfused-fixed with 4% paraformaldehyde, frozen on dry-ice and then sectioned (15 μm-thick) with a cryostat. The sections were then immunostained with anti-GFP and anti-ChAT antibodies, imaged on Leica confocal microscope and manually counted. Data are means ± SEM of 3 independent experiments. **b** DR6 mRNA level was quantified by quantitative RT-PCR in the whole spinal cord from a mouse injected with or without sh-DR6. Data are means of a single independent experiment. Source data provided as source data file.

A.

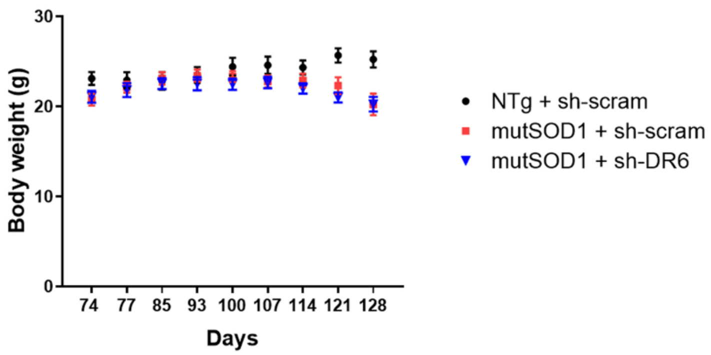

Supplementary Figure 7. Body weight assessment in Tg mutSOD1 mice. a Body weight of NTg mice injected with AAV9-sh-scrambled (sh-scram; n=11) or Tg mutSOD1 mice injected with either AAV9-sh-scram (n=19) or AAV9-sh-DR6 (n=17) was measured over time as a proxy of onset of the paralytic phenotype. Data are means±SEM of independent experiments (n) and were analyzed by two-way ANOVA (Treatment main factor $F_{(2, 391)}$=20.88, P<0.0001) followed by Sidak post-hoc test: For day 121 and 128 timepoint, mutSOD1 + sh-scram was significantly different from NTg + sh-scram and mutSOD1 + sh-scram, but there was no significant difference between mutSOD1 + sh-scram and mutSOD1 + sh-DR6.

Supplementary Table 1. Mass spectrometric analysis of mutSOD1 and NTg astrocyte conditioned media by LC-MS/MS. Fold change represent mutSOD1 – NTg. No change means difference below 1.5 fold.

No.	Uniprot ID	Name	Fold change	Gene Symbol
1	P27605	Hypoxanthine-guanine phosphoribosyltransferase	minus 2.6	Hprt
2	P30121	Metalloproteinase inhibitor 2	minus 6.5	Timp2
3	P25113	Phosphoglycerate mutase 1	minus 2.6	Pgam1
4	P11980	Pyruvate kinase isozymes M1/M2	minus 3	Pkm
5	Q9QZQ5	Protein NOV homolog	minus 4.6	Nov
6	P04937	Fibronectin	minus 4.8	Fn1
7	Q63690	Apoptosis regulator BAX, membrane isoform alpha	No change	Bax
8	P35704	Peroxiredoxin-2	No change	Prdx2
9	P68035	Actin, alpha cardiac muscle 1	No change	Actc1
10	Q4QQW8	Putative phospholipase B-like 2	No change	Plbd2
11	P14844	C-C motif chemokine 2	nTg only	Ccl2
12	Q6P7Q4	Lactoylglutathione lyase	nTg only	Glo1
13	P31044	Phosphatidylethanolamine-binding protein 1	nTg only	Pebp1
14	P30152	Neutrophil gelatinase-associated lipocalin	nTg only	Lcn2
15	P24368	Peptidyl-prolyl cis-trans isomerase B	nTg only	Ppib
16	O35509	Ras-related protein Rab-11B	nTg only	Rab11b
17	P62994	Growth factor receptor-bound protein 2	nTg only	Grb2
18	P48500	Triosephosphate isomerase	nTg only	Tpi1
19	Q9Z0V6	Thioredoxin-dependent peroxide reductase, mitochondrial	nTg only	Prdx3
20	Q9WUQ1	ADAMTS-1	nTg only	Adamts1
21	P30427	Plectin-1	nTg only	Plec
22	P31232	Transgelin	plus 1.9	Tagln
23	Q566C7	Diphosphoinositol polyphosphate phosphohydrolase 1	plus 2.3	Nudt3
24	P14841	Cystatin-C	plus 2.3	Cst3
25	P45592	Cofilin-1	plus 2.5	Cfl1
26	P10960	Sulfated glycoprotein 1	plus 21	Psap
27	P24594	Insulin-like growth factor-binding protein 5	plus 3	Igfbp5
28	P08699	Galectin-3	plus 3	Lgals3
29	P08494	Matrix Gla protein	plus 5	Mgp
30	P12843	Insulin-like growth factor-binding protein 2	plus 6	Igfbp2
31	Q9Z1Y3	Cadherin-2	plus 8.6	Cdh2
32	P05964	Protein S100-A6	Tg only	S100a6
33	P14095	Growth-regulated alpha protein	Tg only	Cxcl1

34	P50229	C-C motif chemokine 3	Tg only	Ccl3
35	P30904	Macrophage migration inhibitory factor	Tg only	Mif
36	P62775	Myotrophin	Tg only	Mtpn
37	P80254	D-dopachrome decarboxylase	Tg only	Ddt
38	P07151	Beta-2-microglobulin	Tg only	B2m
39	P97885	C-X-C motif chemokine 5	Tg only	Cxcl5
40	P11762	Galectin-1	Tg only	Lgals1
41	Q5U318	Astrocytic phosphoprotein PEA-15	Tg only	Pea15a
42	P07632	Superoxide dismutase [Cu-Zn]	Tg only	Sod1
43	P02696	Retinol-binding protein I	Tg only	Rbp1
44	Q05982	Nucleoside diphosphate kinase A	Tg only	Nme1
45	P19804	Nucleoside diphosphate kinase B	Tg only	Nme2
46	O55004	Ribonuclease 4 precursor	Tg only	Rnase4
47	P00697	Lysozyme C type 1	Tg only	Lyz1
48	P08025	Insulin-like growth factor I	Tg only	Igf1
49	Q9EQX9	Ubiquitin-conjugating enzyme E2 N	Tg only	Ube2n
50	P10111	Peptidyl-prolyl cis-trans isomerase A	Tg only	Ppia
51	P41498	Low molecular weight phosphotyrosine protein phosphatase	Tg only	Acp1
52	Q498E0	Thioredoxin domain-containing protein 12	Tg only	Txndc12
53	P62634	Cellular nucleic acid-binding protein	Tg only	Cnbp
54	P18666	Myosin regulatory light chain 12B	Tg only	Myl12b
55	O88767	Protein DJ-1	Tg only	Park7
56	P0C5H9	Mesencephalic astrocyte-derived neurotrophic factor	Tg only	Manf
57	Q9R063	Peroxiredoxin-5, mitochondrial	Tg only	Prdx5
58	Q5FVH0	Complement C1q tumor necrosis factor-related protein 5	Tg only	C1qtnf5
59	Q00981	Ubiquitin carboxyl-terminal hydrolase isozyme L1	Tg only	Uchl1
60	O35244	Peroxiredoxin-6	Tg only	Prdx6
61	Q3T1J9	MOB kinase activator 1A	Tg only	Mob1a
62	P07895	Superoxide dismutase [Mn], mitochondrial	Tg only	Sod2
63	Q4KM74	Vesicle-trafficking protein SEC22b	Tg only	Sec22b
64	Q64361	Latexin	Tg only	Lxn
65	P14942	Glutathione S-transferase alpha-4	Tg only	Gsta4
66	P08010	Glutathione S-transferase Mu 2	Tg only	Gstm2
67	Q5U1X1	Oligoribonuclease, mitochondrial	Tg only	Rexo2
68	Q08420	Extracellular superoxide dismutase [Cu-Zn]	Tg only	Sod3
69	Q66HR2	Microtubule-associated protein RP/EB family member 1	Tg only	Mapre1
70	Q6AYE5	Out at first protein homolog	Tg only	Oaf

71	P06762	Heme oxygenase 1	Tg only	Hmox1
72	P58775	Tropomyosin beta chain	Tg only	Tpm2
73	Q63768	Proto-oncogene C-crk	Tg only	Crk
74	P30919	N(4)-(Beta-N-acetylglucosaminyl)-L-asparaginase precursor	Tg only	Aga
75	Q9R1E9	Connective tissue growth factor	Tg only	Ctgf
76	P06866	Haptoglobin	Tg only	Hp
77	Q794E4	Heterogeneous nuclear ribonucleoprotein F	Tg only	Hnrnpf
78	P47819	Glial fibrillary acidic protein	Tg only	Gfap
79	P61980	Heterogeneous nuclear ribonucleoprotein K	Tg only	Hnrnpk
80	P11598	Protein disulfide-isomerase A3	Tg only	Pdia3
81	Q66H80	Coatomer subunit delta	Tg only	Arcn1
82	P08592	Amyloid beta A4 protein	Tg only	App
83	Q6P7A9	Lysosomal alpha-glucosidase	Tg only	Gaa
84	O35806	Latent-transforming growth factor beta-binding protein 2	Tg only	Ltbp2
85	Q00918	Latent-transforming growth factor beta-binding protein 1	Tg only	Ltbp1
86	P55051	Fatty acid-binding protein, brain	Tg only	Fabp7

Supplementary Table 2. List of putative receptors based on PrePPI database involving candidate ligands. The reported p-values are computed as one-tailed test from a null model based on the empirical distribution of PrePPI scores as described in the Methods section. The function empPvals from the package qvalue-2.8.0 in the R environment version 3.4.1. was used. Since these p-values are not yet integrated in a final list, no multiple hypothesis testing correction was performed.

No.	Ligand Gene Name	Ligand Protein ID	Receptor Gene Name	Receptor Protein ID	p-value
1	CCL3	P10147	CCR1	P32246	2.46804E-05
2	CCL3	P10147	CCR5	P51681	7.40412E-05
3	APP	P05067	APBB1	O00213	7.40412E-05
4	S100A6	P06703	S100A6	P06703	0.000123402
5	CCL3	P10147	CCR4	P51679	0.000222124
6	CDH2	P19022	CTNND1	O60716	0.000246804
7	B2M	P61769	CD8A	P01732	0.000296165
8	LGALS1	P09382	SPN	P16150	0.000296165
9	MAPRE1	Q15691	RAB35	Q15286	0.000320845
10	CRK	P46108	ARHGAP17	Q68EM7	0.000320845
11	HNRNPK	P61978	LYN	P07948	0.000320845
12	APP	P05067	PSEN1	P49768	0.000320845
13	CDH2	P19022	GRIK2	Q13002	0.000370206
14	SOD1	P00441	SRI	P30626	0.000370206
15	CTGF	P29279	ITGA5	P08648	0.000370206
16	APP	P05067	HOMER3	Q9NSC5	0.000370206
17	APP	P05067	PTK2	Q05397	0.000419567
18	CCL3	P10147	CD28	P10747	0.000444247
19	CTGF	P29279	FGFR3	P22607	0.000444247
20	PDIA3	P30101	ATP6AP2	O75787	0.000444247
21	APP	P05067	HOMER2	Q9NSB8	0.000468927
22	MAPRE1	Q15691	SPTBN1	Q01082	0.000518288
23	CDH2	P19022	GRIN1	Q05586	0.000542969
24	HP	P00738	ITGB2	P05107	0.000567649
25	APP	P05067	AKT1	P31749	0.000567649
26	CXCL6	P80162	CXCR1	P25024	0.000592329
27	CDH2	P19022	GNA12	Q03113	0.00061701
28	CCL3	P10147	CX3CL1	P78423	0.00061701
29	CRK	P46108	ZAP70	P43403	0.00061701
30	APP	P05067	NCSTN	Q92542	0.00061701
31	B2M	P61769	KLRK1	P26718	0.00064169
32	B2M	P61769	KLRK1	P26718	0.00064169
33	PEA15	Q15121	PLD2	O14939	0.000666371

34	NUDT3	O95989	RAB17	Q9H0T7	0.000691051
35	CTGF	P29279	VEGFA	P15692	0.000691051
36	CFL1	P23528	IQGAP1	P46940	0.000715731
37	CDH2	P19022	ARHGAP32	A7KAX9	0.000715731
38	B2M	P61769	IL7R	P16871	0.000740412
39	APP	P05067	TNFRSF21	O75509	0.000740412
40	CDH2	P19022	RYK	P34925	0.000765092
41	B2M	P61769	FCER1A	P12319	0.000765092
42	CRK	P46108	EPS15	P42566	0.000789772
43	CFL1	P23528	PLD2	O14939	0.000839133
44	B2M	P61769	IL6ST	P40189	0.000839133
45	CRK	P46108	DOK3	Q7L591	0.000863814
46	CRK	P46108	RAB2B	Q8WUD1	0.000863814
47	PDIA3	P30101	PLXNA1	Q9UIW2	0.000863814
48	CDH2	P19022	GNA13	Q14344	0.000888494
49	CRK	P46108	FRS2	Q8WU20	0.000937855
50	APP	P05067	BTK	Q06187	0.000937855
51	B2M	P61769	CXCR4	P61073	0.000962535
52	CRK	P46108	ERBB2	P04626	0.000962535
53	CTGF	P29279	LRP1	Q07954	0.000962535
54	B2M	P61769	CD74	P04233	0.001011896
55	CFL1	P23528	PSEN1	P49768	0.001061257
56	IGF1	P05019	INSR	P06213	0.001061257
57	PARK7	Q99497	PRKN	O60260	0.001061257
58	LGALS1	P09382	PTPRC	P08575	0.001085937
59	HP	P00738	SORT1	Q99523	0.001159978
60	B2M	P61769	CD80	P33681	0.001184659
61	IGF1	P05019	SORBS1	Q9BX66	0.001184659
62	NME2	P22392	RAB5A	P20339	0.001209339
63	MYL12B	O14950	SRI	P30626	0.001209339
64	APP	P05067	CHRNA7	P36544	0.001209339
65	CXCL6	P80162	CX3CL1	P78423	0.001234019
66	ARCN1	P48444	ITGA4	P13612	0.0012587
67	LGALS1	P09382	NCAM1	P13591	0.00128338
68	CRK	P46108	PTK2	Q05397	0.00128338
69	FABP7	O15540	TNS2	Q63HR2	0.001308061
70	MTPN	P58546	PPP1R16B	Q96T49	0.001332741
71	B2M	P61769	CD28	P10747	0.001332741
72	B2M	P61769	RAB27A	P51159	0.001357421
73	CRK	P46108	PTK2B	Q14289	0.001357421
74	APP	P05067	DLG4	P78352	0.001357421
75	LGALS1	P09382	KDR	P35968	0.001382102
76	APP	P05067	NTRK3	Q16288	0.001382102

77	CCL3	P10147	CD4	P01730	0.001431463
78	MAPRE1	Q15691	ROCK2	O75116	0.001431463
79	CST3	P01034	ENG	P17813	0.001480823
80	HNRNPK	P61978	ITK	Q08881	0.001480823
81	APP	P05067	CDK5R1	Q15078	0.001480823
82	CST3	P01034	IL6R	P08887	0.001505504
83	SOD1	P00441	SPTBN1	Q01082	0.001505504
84	APP	P05067	NGFR	P08138	0.001505504
85	LTBP2	Q14767	SMURF2	Q9HAU4	0.001505504
86	CST3	P01034	ITGB3	P05106	0.001530184
87	LGALS3	P17931	NCSTN	Q92542	0.001530184
88	ARCN1	P48444	EPS15	P42566	0.001579545
89	APP	P05067	PSEN2	P49810	0.001579545
90	APP	P05067	DNM2	P50570	0.001579545
91	CDH2	P19022	L1CAM	P32004	0.001604225
92	AGA	P20933	NCSTN	Q92542	0.001604225
93	NME2	P22392	PRKN	O60260	0.001653586
94	CST3	P01034	HBEGF	Q99075	0.001727627
95	CRK	P46108	ERBB3	P21860	0.001727627
96	NME2	P22392	TBXA2R	P21731	0.001752308
97	APP	P05067	PRKN	O60260	0.001776988
98	B2M	P61769	NCR1	O76036	0.001801668
99	CRK	P46108	KIT	P10721	0.001801668
100	CST3	P01034	NCSTN	Q92542	0.001826349
101	CCL3	P10147	CD83	Q01151	0.001826349
102	SEC22B	O75396	VAPB	O95292	0.00187571
103	B2M	P61769	LAG3	P18627	0.00192507
104	CRK	P46108	FGFR1	P11362	0.00192507
105	CDH2	P19022	GJA1	P17302	0.001949751
106	CFL1	P23528	RAB10	P61026	0.001974431
107	CDH2	P19022	SPTBN1	Q01082	0.001974431
108	APP	P05067	BAIAP2	Q9UQB8	0.001999112
109	LGALS1	P09382	ATP6AP2	O75787	0.002023792
110	MAPRE1	Q15691	MIB1	Q86YT6	0.002023792
111	CRK	P46108	ARHGAP32	A7KAX9	0.002073153
112	CDH2	P19022	DDR1	Q08345	0.002097833
113	CDH2	P19022	RAB8B	Q92930	0.002097833
114	B2M	P61769	IL4R	P24394	0.002097833
115	CRK	P46108	FASLG	P48023	0.002097833
116	B2M	P61769	CD3E	P07766	0.002122513
117	CRK	P46108	GAB2	Q9UQC2	0.002147194
118	CRK	P46108	NTRK1	P04629	0.002171874
119	CRK	P46108	FYN	P06241	0.002171874

120	GFAP	P14136	PSEN2	P49810	0.002171874
121	S100A6	P06703	NGFR	P08138	0.002221235
122	CST3	P01034	ILK	Q13418	0.002245915
123	B2M	P61769	CD4	P01730	0.002245915
124	ARCN1	P48444	RAB31	Q13636	0.002245915
125	B2M	P61769	TNFSF13B	Q9Y275	0.002270596
126	APP	P05067	GLRB	P48167	0.002270596
127	B2M	P61769	CD47	Q08722	0.002319957
128	GFAP	P14136	PSEN1	P49768	0.002319957
129	APP	P05067	LRP1	Q07954	0.002344637
130	LGALS3	P17931	LPAR1	Q92633	0.002393998
131	APP	P05067	GPR12	P47775	0.002393998
132	NME1	P15531	RRAD	P55042	0.002418678
133	SOD2	P04179	TLR2	O60603	0.002443359
134	CRK	P46108	INSR	P06213	0.002443359
135	ARCN1	P48444	PRKCI	P41743	0.002443359
136	APP	P05067	TGFBR2	P37173	0.002468039
137	PARK7	Q99497	RAB3A	P20336	0.002492719
138	CFL1	P23528	NTRK1	P04629	0.0025174
139	LGALS3	P17931	SLC12A2	P55011	0.0025174
140	CXCL6	P80162	SELE	P16581	0.00254208
141	CRK	P46108	ELMO1	Q92556	0.00254208
142	B2M	P61769	CD274	Q9NZQ7	0.002616121
143	B2M	P61769	ERBB3	P21860	0.002640802
144	LGALS1	P09382	FLT1	P17948	0.002665482
145	CST3	P01034	FURIN	P09958	0.002690162
146	CST3	P01034	INPP5D	Q92835	0.002714843
147	CRK	P46108	FLT1	P17948	0.002739523
148	B2M	P61769	BSG	P35613	0.002788884
149	HNRNPF	P52597	RAB14	P61106	0.002813564
150	ARCN1	P48444	PSEN1	P49768	0.002813564
151	APP	P05067	NAE1	Q13564	0.002813564
152	ARCN1	P48444	RAB5A	P20339	0.002838245
153	IGF1	P05019	ITSN1	Q15811	0.002862925
154	HNRNPK	P61978	NEDD4	P46934	0.002862925
155	B2M	P61769	CD83	Q01151	0.002936966
156	HP	P00738	ITGAM	P11215	0.002936966
157	CST3	P01034	VEGFA	P15692	0.002961647
158	SOD2	P04179	VAPA	Q9P0L0	0.002986327
159	HNRNPF	P52597	ILK	Q13418	0.002986327
160	SOD2	P04179	SRI	P30626	0.003011007
161	NME2	P22392	RHOB	P62745	0.003035688
162	CRK	P46108	EPHB3	P54753	0.003035688

163	IGFBP5	P24593	NOS3	P29474	0.003085049
164	HNRNPF	P52597	NCSTN	Q92542	0.003085049
165	HNRNPK	P61978	RASD1	Q9Y272	0.003085049
166	LGALS3	P17931	P2RY12	Q9H244	0.003109729
167	B2M	P61769	TNFSF12	O43508	0.003109729
168	IGF1	P05019	IL31RA	Q8NI17	0.00315909
169	B2M	P61769	CD48	P09326	0.00318377
170	CRK	P46108	PDGFRA	P16234	0.003208451
171	CTGF	P29279	HBEGF	Q99075	0.003208451
172	IGF1	P05019	IL6ST	P40189	0.003257811
173	B2M	P61769	CD40	P25942	0.003282492
174	CRK	P46108	SH2D2A	Q9NP31	0.003307172
175	APP	P05067	MAST1	Q9Y2H9	0.003331853
176	CTGF	P29279	F2R	P25116	0.003356533
177	CRK	P46108	EPHA3	P29320	0.003381213
178	CTGF	P29279	ITGB5	P18084	0.003381213
179	CST3	P01034	LRP1	Q07954	0.003405894
180	B2M	P61769	PTPRC	P08575	0.003405894
181	IGF1	P05019	JAK2	O60674	0.003405894
182	CRK	P46108	RRAS	P10301	0.003430574
183	IGFBP5	P24593	IL6ST	P40189	0.003455254
184	CDH2	P19022	IQGAP1	P46940	0.003455254
185	IGF1	P05019	LYN	P07948	0.003479935
186	PEA15	Q15121	SORT1	Q99523	0.003553976
187	HMOX1	P09601	RALB	P11234	0.003578656
188	GAA	P10253	P2RX4	Q99571	0.003578656
189	CST3	P01034	NOS3	P29474	0.003603337
190	IGF1	P05019	TEK	Q02763	0.003603337
191	CDH2	P19022	NTRK1	P04629	0.003628017
192	IGF1	P05019	NGEF	Q8N5V2	0.003628017
193	APP	P05067	EPHB4	P54760	0.003652698
194	CNBP	P62633	RAP1B	P61224	0.003677378
195	HNRNPK	P61978	LGR4	Q9BXB1	0.003677378
196	CDH2	P19022	PTPRC	P08575	0.003702058
197	HMOX1	P09601	SRI	P30626	0.003726739
198	HMOX1	P09601	PTPRE	P23469	0.003726739
199	B2M	P61769	IL2RA	P01589	0.003751419
200	CNBP	P62633	ITGA4	P13612	0.0037761
201	B2M	P61769	FAS	P25445	0.00380078
202	B2M	P61769	NFAM1	Q8NET5	0.00382546
203	SOD2	P04179	TLR1	Q15399	0.00382546
204	LGALS1	P09382	CX3CL1	P78423	0.003850141
205	ARCN1	P48444	CRK	P46108	0.003874821

206	CDH2	P19022	CELSR1	Q9NYQ6	0.003924182
207	CTGF	P29279	ROR2	Q01974	0.003973543
208	HNRNPK	P61978	ITGA4	P13612	0.004022903
209	CDH2	P19022	CELSR3	Q9NYQ7	0.004047584
210	LGALS1	P09382	S100A6	P06703	0.004047584
211	GAA	P10253	TRAF4	Q9BUZ4	0.004047584
212	CRK	P46108	EPHB2	P29323	0.004072264
213	IGFBP5	P24593	ILK	Q13418	0.004096945
214	CRK	P46108	PDGFRB	P09619	0.004096945
215	IGFBP5	P24593	ITGB3	P05106	0.004121625
216	CFL1	P23528	RAB35	Q15286	0.004170986
217	CDH2	P19022	ITGA4	P13612	0.004170986
218	B2M	P61769	FCGR2B	P31994	0.004170986
219	HMOX1	P09601	LAT2	Q9GZY6	0.004170986
220	CDH2	P19022	CDH2	P19022	0.004195666
221	CCL3	P10147	TNF	P01375	0.004245027
222	APP	P05067	CD74	P04233	0.004269707
223	CDH2	P19022	ITGA5	P08648	0.004343748
224	APP	P05067	RAB3A	P20336	0.004343748
225	B2M	P61769	PLD2	O14939	0.004368429
226	B2M	P61769	SLA2	Q9H6Q3	0.004368429
227	LGALS1	P09382	RAC1	P63000	0.004368429
228	CTGF	P29279	FGFR1	P11362	0.004368429
229	IGFBP5	P24593	FLT1	P17948	0.00441779
230	B2M	P61769	CD79A	P11912	0.00441779
231	CFL1	P23528	CRK	P46108	0.00444247
232	B2M	P61769	THY1	P04216	0.00444247
233	NME1	P15531	PTPRN	Q16849	0.00446715
234	HMOX1	P09601	IL4R	P24394	0.004491831
235	CST3	P01034	ITGA4	P13612	0.004516511
236	LGALS1	P09382	PTPRU	Q92729	0.004516511
237	MAPRE1	Q15691	UBE2B	P63146	0.004541192
238	CRK	P46108	KDR	P35968	0.004541192
239	CST3	P01034	P2RX7	Q99572	0.004590552
240	B2M	P61769	CD2	P06729	0.004590552
241	LGALS1	P09382	CD164	Q04900	0.004615233
242	CNBP	P62633	RAP1A	P62834	0.004615233
243	LGALS1	P09382	NF2	P35240	0.004664594
244	CRK	P46108	GHR	P10912	0.004738635
245	CFL1	P23528	MARK2	Q7KZI7	0.004812676
246	APP	P05067	RAB5C	P51148	0.004837356
247	B2M	P61769	INPP5D	Q92835	0.004862037
248	ARCN1	P48444	RAB14	P61106	0.004862037

249	B2M	P61769	TNF	P01375	0.004886717
250	IGF1	P05019	PLPP1	O14494	0.004886717
251	HP	P00738	KDR	P35968	0.004911397
252	APP	P05067	TEK	Q02763	0.004911397
253	APP	P05067	RAB27A	P51159	0.004960758
254	IGF1	P05019	SLA2	Q9H6Q3	0.005034799
255	LGALS3	P17931	FLT4	P35916	0.00505948
256	ARCN1	P48444	RAB5C	P51148	0.00505948
257	B2M	P61769	BTLA	Q7Z6A9	0.00508416
258	B2M	P61769	IFNAR2	P48551	0.005108841
259	LGALS1	P09382	PTGER3	P43115	0.005158201
260	HP	P00738	SRI	P30626	0.005158201
261	CFL1	P23528	NF2	P35240	0.005182882
262	APP	P05067	APLP2	Q06481	0.005232242
263	CDH2	P19022	CELSR2	Q9HCU4	0.005256923
264	CRK	P46108	IRS4	O14654	0.005281603
265	NME1	P15531	RAC1	P63000	0.005330964
266	IGF1	P05019	IL1RL1	Q01638	0.005355644
267	CCL3	P10147	CD8A	P01732	0.005380325
268	LGALS1	P09382	THY1	P04216	0.005380325
269	CDH2	P19022	CRK	P46108	0.005405005
270	CRK	P46108	RGS20	O76081	0.005429686
271	APP	P05067	CAV1	Q03135	0.005479046
272	CST3	P01034	CD74	P04233	0.005503727
273	CFL1	P23528	RALB	P11234	0.005503727
274	IGFBP5	P24593	TNS2	Q63HR2	0.005503727
275	CDH2	P19022	ALCAM	Q13740	0.005503727
276	B2M	P61769	IL6R	P08887	0.005528407
277	CST3	P01034	PDPK1	O15530	0.005553088
278	AGA	P20933	PSEN1	P49768	0.005651809
279	HMOX1	P09601	IL7R	P16871	0.005824572
280	HNRNPF	P52597	PRKN	O60260	0.005824572
281	IGF1	P05019	PLXND1	Q9Y4D7	0.005849252
282	B2M	P61769	VEGFA	P15692	0.005898613
283	CFL1	P23528	SCRIB	Q14160	0.005923293
284	IGF1	P05019	S100A6	P06703	0.005923293
285	PARK7	Q99497	RAB4A	P20338	0.005947974
286	MAPRE1	Q15691	NTRK1	P04629	0.005947974
287	HP	P00738	F2R	P25116	0.006022015
288	ARCN1	P48444	RAB8A	P61006	0.006022015
289	HMOX1	P09601	TLR2	O60603	0.006046695
290	CFL1	P23528	ILK	Q13418	0.006071376
291	LGALS1	P09382	EDA2R	Q9HAV5	0.006071376

292	PARK7	Q99497	TRAF4	Q9BUZ4	0.006071376
293	LXN	Q9BS40	NGFR	P08138	0.006071376
294	IGF1	P05019	ITGA4	P13612	0.006096056
295	CRK	P46108	DNM2	P50570	0.006096056
296	PDIA3	P30101	SRI	P30626	0.006120736
297	HMOX1	P09601	RAB3C	Q96E17	0.006145417
298	HMOX1	P09601	ITGB2	P05107	0.006219458
299	B2M	P61769	IL12RB2	Q99665	0.006268819
300	CTGF	P29279	IKBKB	O14920	0.006268819
301	B2M	P61769	IL2RG	P31785	0.00634286
302	IGFBP5	P24593	AGER	Q15109	0.006392221
303	CDH2	P19022	THY1	P04216	0.006392221
304	HMOX1	P09601	IL6R	P08887	0.006392221
305	CRK	P46108	PAG1	Q9NWQ8	0.006392221
306	CDH2	P19022	ITGB1	P05556	0.006441582
307	B2M	P61769	OSMR	Q99650	0.006466262
308	B2M	P61769	JAG1	P78504	0.006466262
309	HMOX1	P09601	RAB26	Q9ULW5	0.006466262
310	HP	P00738	TMPRSS6	Q8IU80	0.006515623
311	CST3	P01034	GRIN2C	Q14957	0.006564983
312	ARCN1	P48444	GOLPH3	Q9H4A6	0.006564983
313	CCL3	P10147	VAPB	O95292	0.006589664
314	CRK	P46108	RHOU	Q7L0Q8	0.006639025
315	IGF1	P05019	NGFR	P08138	0.006663705
316	IGFBP2	P18065	ITGA2	P17301	0.006688385
317	ARCN1	P48444	RAB18	Q9NP72	0.006688385
318	CDH2	P19022	INSR	P06213	0.006713066
319	CDH2	P19022	LRP6	O75581	0.006762427
320	HMOX1	P09601	LRP1	Q07954	0.006787107
321	B2M	P61769	CX3CL1	P78423	0.006861148
322	PDIA3	P30101	RAB5C	P51148	0.006910509
323	GAA	P10253	RAB3A	P20336	0.006935189
324	LTBP2	Q14767	TGFBR2	P37173	0.006935189
325	IGF1	P05019	P2RY6	Q15077	0.00695987
326	PRDX6	P30041	RHOA	P61586	0.00695987
327	PRDX6	P30041	RHOA	P61586	0.00695987
328	ARCN1	P48444	RAB27A	P51159	0.00695987
329	MTPN	P58546	SMO	Q99835	0.00698455
330	CRK	P46108	RAB14	P61106	0.00698455
331	CST3	P01034	ITGA2	P17301	0.007033911
332	APP	P05067	PRKCI	P41743	0.007033911
333	IGFBP5	P24593	PRKCA	P17252	0.007058591
334	CFL1	P23528	RAB11A	P62491	0.007107952

335	LGALS3	P17931	RAB11B	Q15907	0.007107952
336	HMOX1	P09601	RAB27A	P51159	0.007107952
337	B2M	P61769	TNFSF14	O43557	0.007132632
338	IGFBP5	P24593	PDGFRA	P16234	0.007157313
339	CST3	P01034	GHR	P10912	0.007181993
340	IGFBP5	P24593	PDGFRB	P09619	0.007206674
341	MTPN	P58546	TACR3	P29371	0.007231354
342	CRK	P46108	NISCH	Q9Y2I1	0.007231354
343	CTGF	P29279	IL6R	P08887	0.007231354
344	CRK	P46108	GNA13	Q14344	0.007280715
345	B2M	P61769	SH2B2	O14492	0.007305395
346	HNRNPF	P52597	PLP2	Q04941	0.007305395
347	LGALS1	P09382	RAB10	P61026	0.007330076
348	HNRNPK	P61978	PRKN	O60260	0.007330076
349	CDH2	P19022	CD164	Q04900	0.007354756
350	IGF1	P05019	TNS2	Q63HR2	0.007354756
351	PDIA3	P30101	RAB5A	P20339	0.007379436
352	B2M	P61769	TLR2	O60603	0.007404117
353	CST3	P01034	DNER	Q8NFT8	0.007428797
354	IGF1	P05019	PDGFRA	P16234	0.007428797
355	TPM2	P07951	FLT1	P17948	0.007453477
356	APP	P05067	GNA12	Q03113	0.007453477
357	CRK	P46108	RAB5A	P20339	0.007502838
358	CST3	P01034	GRIN2A	Q12879	0.007527519
359	LGALS1	P09382	PLD2	O14939	0.007527519
360	CFL1	P23528	PRKCI	P41743	0.00760156
361	IGF1	P05019	SKAP1	Q86WV1	0.007700281
362	CTGF	P29279	SRI	P30626	0.007724962
363	APP	P05067	CRHR2	Q13324	0.007724962
364	LTBP2	Q14767	CD40	P25942	0.007724962
365	CRK	P46108	RHOG	P84095	0.007823683
366	B2M	P61769	FCGR1A	P12314	0.007873044
367	CDH2	P19022	GLRB	P48167	0.007897724
368	ARCN1	P48444	P2RX7	Q99572	0.007922405
369	CFL1	P23528	PARD3	Q8TEW0	0.007971766
370	B2M	P61769	GABRE	P78334	0.008045807
371	CRK	P46108	GABBR1	Q9UBS5	0.008070487
372	CDH2	P19022	ITGAL	P20701	0.008095168
373	IGF1	P05019	AVPR1A	P37288	0.008119848
374	CRK	P46108	RALB	P11234	0.008119848
375	CDH2	P19022	NGEF	Q8N5V2	0.008169209
376	B2M	P61769	BCL10	O95999	0.008169209
377	NME1	P15531	TRAF4	Q9BUZ4	0.008169209

378	CTGF	P29279	GRIN2A	Q12879	0.008193889
379	CDH2	P19022	ITGB3	P05106	0.00824325
380	APP	P05067	RGS14	O43566	0.00824325
381	CST3	P01034	F2R	P25116	0.00826793
382	HMOX1	P09601	PLD2	O14939	0.00826793
383	GAA	P10253	SORBS1	Q9BX66	0.008292611
384	CRK	P46108	ARHGAP10	A1A4S6	0.008317291
385	CDH2	P19022	PTPRF	P10586	0.008366652
386	CDH2	P19022	ILK	Q13418	0.008416013
387	CDH2	P19022	LINGO1	Q96FE5	0.008416013
388	HMOX1	P09601	PTPRC	P08575	0.008416013
389	CST3	P01034	PDGFRA	P16234	0.008440693
390	B2M	P61769	INSR	P06213	0.008440693
391	MAPRE1	Q15691	RALB	P11234	0.008440693
392	ARCN1	P48444	MAP4K2	Q12851	0.008440693
393	CTGF	P29279	PKD2	Q13563	0.008490054
394	CFL1	P23528	RHOA	P61586	0.008514734
395	CRK	P46108	ITSN1	Q15811	0.008514734
396	B2M	P61769	CDH13	P55290	0.008539415
397	HMOX1	P09601	RHOB	P62745	0.008539415
398	CST3	P01034	TNF	P01375	0.008613456
399	CDH2	P19022	SELE	P16581	0.008613456
400	IGF1	P05019	KIT	P10721	0.008613456
401	CFL1	P23528	ROCK2	O75116	0.008638136
402	HNRNPK	P61978	PIK3C2A	O00443	0.008662817
403	CDH2	P19022	ROBO1	Q9Y6N7	0.008712177
404	LGALS1	P09382	PTPRF	P10586	0.008736858
405	CFL1	P23528	IKBKB	O14920	0.008810899
406	B2M	P61769	ERBB2	P04626	0.008810899
407	APP	P05067	ITGA6	P23229	0.008810899
408	CDH2	P19022	EPHB2	P29323	0.008835579
409	IGF1	P05019	ILK	Q13418	0.008835579
410	CDH2	P19022	NCAM1	P13591	0.00886026
411	IGF1	P05019	KITLG	P21583	0.00886026
412	CFL1	P23528	PSD4	Q8NDX1	0.00888494
413	CRK	P46108	SORBS1	Q9BX66	0.008934301
414	CST3	P01034	FASLG	P48023	0.008958981
415	ARCN1	P48444	DNM2	P50570	0.008983662
416	CRK	P46108	GNA12	Q03113	0.009008342
417	CDH2	P19022	EPHA7	Q15375	0.009082383
418	HNRNPK	P61978	WWP1	Q9H0M0	0.009082383
419	CDH2	P19022	ITPR3	Q14573	0.009181105
420	IGF1	P05019	NOD1	Q9Y239	0.009181105

421	CRK	P46108	RHOA	P61586	0.009181105
422	CFL1	P23528	KITLG	P21583	0.009205785
423	ARCN1	P48444	RAB11A	P62491	0.009205785
424	B2M	P61769	ZAP70	P43403	0.009255146
425	B2M	P61769	LCK	P06239	0.009255146
426	IGF1	P05019	PSEN2	P49810	0.009329187
427	HMOX1	P09601	RAB3D	O95716	0.009329187
428	CCL3	P10147	FASLG	P48023	0.009378548
429	CNBP	P62633	RAB5A	P20339	0.009378548
430	CRK	P46108	ROCK2	O75116	0.009378548
431	CTGF	P29279	ILK	Q13418	0.009378548
432	CST3	P01034	FGFR1	P11362	0.009403228
433	CXCL6	P80162	IL6ST	P40189	0.009403228
434	LGALS1	P09382	TNF	P01375	0.00950195
435	PARK7	Q99497	RAC1	P63000	0.00952663
436	IGF1	P05019	ITK	Q08881	0.009551311
437	IGF1	P05019	SMO	Q99835	0.009551311
438	APP	P05067	RAB43	Q86YS6	0.009551311
439	B2M	P61769	SHH	Q15465	0.009575991
440	HMOX1	P09601	IL6ST	P40189	0.009575991
441	APP	P05067	RAB2B	Q8WUD1	0.009625352
442	CRK	P46108	LYN	P07948	0.009699393
443	CRK	P46108	RAB35	Q15286	0.009798114
444	APP	P05067	PDPK1	O15530	0.009798114
445	CST3	P01034	HOMER1	Q86YM7	0.009822795
446	IGF1	P05019	LCK	P06239	0.009822795
447	CRK	P46108	RAP1B	P61224	0.009822795
448	MTPN	P58546	PRKN	O60260	0.009872156
449	ARCN1	P48444	RAB10	P61026	0.009896836
450	B2M	P61769	IL31RA	Q8NI17	0.009970877
451	IGFBP5	P24593	RRAS	P10301	0.009995558
452	HNRNPF	P52597	NTRK1	P04629	0.009995558
453	CFL1	P23528	EPHA2	P29317	0.010020238
454	HNRNPK	P61978	VAPB	O95292	0.010020238
455	B2M	P61769	EPHA3	P29320	0.010044918
456	CDH2	P19022	KCNH6	Q9H252	0.010069599
457	CFL1	P23528	PTK2B	Q14289	0.010094279
458	HMOX1	P09601	NEDD4	P46934	0.01014364
459	HMOX1	P09601	TNFRSF1A	P19438	0.01016832
460	HMOX1	P09601	RALA	P11233	0.010242361
461	CDH2	P19022	CD8A	P01732	0.010267042
462	CRK	P46108	RAB9A	P51151	0.010316403
463	CFL1	P23528	CSK	P41240	0.010341083

464	HMOX1	P09601	TLR7	Q9NYK1	0.010341083
465	LGALS1	P09382	NGFR	P08138	0.010415124
466	PEA15	Q15121	SORBS1	Q9BX66	0.010415124
467	APP	P05067	EPHA2	P29317	0.010415124
468	MAPRE1	Q15691	PDPK1	O15530	0.010464485
469	IGFBP5	P24593	ITGA2	P17301	0.010489165
470	CRK	P46108	RAB8B	Q92930	0.010489165
471	CRK	P46108	RAB22A	Q9UL26	0.010489165
472	IGFBP5	P24593	THY1	P04216	0.010513846
473	HNRNPK	P61978	RASA2	Q15283	0.010513846
474	CST3	P01034	SORT1	Q99523	0.010538526
475	CRK	P46108	RASA2	Q15283	0.010587887
476	CRK	P46108	RAB11A	P62491	0.010612567
477	CRK	P46108	RAB5C	P51148	0.010612567
478	B2M	P61769	CD79B	P40259	0.010686608
479	CRK	P46108	RAB10	P61026	0.010735969
480	CTGF	P29279	THY1	P04216	0.010735969
481	CRK	P46108	PRKCI	P41743	0.010834691
482	HNRNPK	P61978	HOMER1	Q86YM7	0.010908732
483	CTGF	P29279	ITGB1	P05556	0.010958093
484	ARCN1	P48444	ITGAV	P06756	0.011032134
485	APP	P05067	GNAZ	P19086	0.011032134
486	LGALS1	P09382	TNFRSF1A	P19438	0.011081495
487	LGALS1	P09382	RHOC	P08134	0.011106175
488	CRK	P46108	G3BP1	Q13283	0.011106175
489	CDH2	P19022	ERBB2	P04626	0.011130855
490	B2M	P61769	CD19	P15391	0.011155536
491	IGF1	P05019	RHOH	Q15669	0.011155536
492	IGF1	P05019	KDR	P35968	0.011155536
493	CFL1	P23528	LRP6	O75581	0.011180216
494	CRK	P46108	RAB8A	P61006	0.011180216
495	CST3	P01034	ERBB3	P21860	0.011204897
496	MAPRE1	Q15691	RYK	P34925	0.011204897
497	CRK	P46108	MCF2L	O15068	0.011229577
498	CRK	P46108	RALA	P11233	0.011254257
499	IGFBP5	P24593	VEGFA	P15692	0.011278938
500	MAPRE1	Q15691	RAC1	P63000	0.011303618
501	HNRNPK	P61978	MAGI3	Q5TCQ9	0.011328299
502	PDIA3	P30101	SDCBP	O00560	0.011328299
503	CRK	P46108	SDCBP	O00560	0.011352979
504	B2M	P61769	NCSTN	Q92542	0.01142702
505	ARCN1	P48444	SDCBP	O00560	0.01142702
506	B2M	P61769	FASLG	P48023	0.0114517

507	CST3	P01034	ITGA5	P08648	0.011501061
508	CDH2	P19022	KCNH2	Q12809	0.011525742
509	CXCL6	P80162	FASLG	P48023	0.011525742
510	CRK	P46108	RAB18	Q9NP72	0.011525742
511	CFL1	P23528	TRAF4	Q9BUZ4	0.011599783
512	CDH2	P19022	PLXNB1	O43157	0.011599783
513	CRK	P46108	IQGAP1	P46940	0.011599783
514	CDH2	P19022	MCF2L	O15068	0.011624463
515	CST3	P01034	SPTBN1	Q01082	0.011673824
516	CRK	P46108	PSEN1	P49768	0.011747865
517	CRK	P46108	RRAS2	P62070	0.011747865
518	HMOX1	P09601	ILK	Q13418	0.011797226
519	IGF1	P05019	NF2	P35240	0.011821906
520	CFL1	P23528	GPR65	Q8IYL9	0.011846587
521	LGALS1	P09382	RAB5C	P51148	0.011846587
522	CRK	P46108	F2RL2	O00254	0.011846587
523	CTGF	P29279	INSR	P06213	0.011846587
524	CFL1	P23528	RALA	P11233	0.011945308
525	B2M	P61769	FGD2	Q7Z6J4	0.011945308
526	CFL1	P23528	ENG	P17813	0.012019349
527	MTPN	P58546	SPTBN1	Q01082	0.012019349
528	IGF1	P05019	NEDD4	P46934	0.012019349
529	CTGF	P29279	ITGA2	P17301	0.01206871
530	HMOX1	P09601	SLA2	Q9H6Q3	0.012093391
531	CRK	P46108	KITLG	P21583	0.012093391
532	IGF1	P05019	PLEKHA1	Q9HB21	0.012118071
533	CRK	P46108	ARHGAP33	O14559	0.012118071
534	APP	P05067	ZAP70	P43403	0.012118071
535	MYL12B	O14950	PSEN2	P49810	0.012192112
536	APP	P05067	ARL4C	P56559	0.012216793
537	B2M	P61769	ITGB1	P05556	0.012241473
538	CFL1	P23528	CTNND1	O60716	0.012290834
539	CDH2	P19022	MYO6	Q9UM54	0.012290834
540	CCL3	P10147	PTK2B	Q14289	0.012290834
541	LGALS1	P09382	NTRK3	Q16288	0.012290834
542	IGF1	P05019	F2R	P25116	0.012315514
543	CFL1	P23528	SH2D2A	Q9NP31	0.012340194
544	ARCN1	P48444	RAB4A	P20338	0.012340194
545	CCL3	P10147	WWP1	Q9H0M0	0.012414236
546	CDH2	P19022	PRKCA	P17252	0.012463596
547	B2M	P61769	IKBKB	O14920	0.012488277
548	ARCN1	P48444	RAB22A	Q9UL26	0.012488277
549	CFL1	P23528	FGFR1	P11362	0.012512957

550	CDH2	P19022	EPHA4	P54764	0.012512957
551	CRK	P46108	RHOB	P62745	0.012512957
552	B2M	P61769	HBEGF	Q99075	0.012537638
553	CFL1	P23528	SDCBP	O00560	0.012611679
554	CFL1	P23528	G3BP1	Q13283	0.012611679
555	NME2	P22392	TGFBR1	P36897	0.012611679
556	CTGF	P29279	TGFBR2	P37173	0.012636359
557	B2M	P61769	KLRD1	Q13241	0.01268572
558	NME1	P15531	INPP5D	Q92835	0.01268572
559	IGFBP5	P24593	TIE1	P35590	0.0127104
560	CFL1	P23528	SH2B2	O14492	0.012809122
561	MAPRE1	Q15691	KDR	P35968	0.012809122
562	APP	P05067	RASGRP2	Q7LDG7	0.012833802
563	CDH2	P19022	PTK2	Q05397	0.012858483
564	CRK	P46108	PDPK1	O15530	0.012907843
565	HNRNPK	P61978	ITGAV	P06756	0.012932524
566	CST3	P01034	THY1	P04216	0.012957204
567	CFL1	P23528	RRAS	P10301	0.012957204
568	B2M	P61769	TREML1	Q86YW5	0.012957204
569	CST3	P01034	ERBB2	P04626	0.013031245
570	PARK7	Q99497	RHOA	P61586	0.013080606
571	CRK	P46108	RAP1A	P62834	0.013105287
572	APP	P05067	RAB26	Q9ULW5	0.013105287
573	CFL1	P23528	STX2	P32856	0.013129967
574	CDH2	P19022	PTPRT	O14522	0.013129967
575	CTGF	P29279	LCK	P06239	0.013154647
576	APP	P05067	ROR1	Q01973	0.013154647
577	CST3	P01034	SHH	Q15465	0.013179328
578	HNRNPK	P61978	ITGB1	P05556	0.013204008
579	IGFBP5	P24593	TNF	P01375	0.013228688
580	CRK	P46108	RAC1	P63000	0.013228688
581	HP	P00738	LCK	P06239	0.013228688
582	PDIA3	P30101	RAB11A	P62491	0.013253369
583	CRK	P46108	RIT1	Q92963	0.013278049
584	CDH2	P19022	ITGB2	P05107	0.01330273
585	IGF1	P05019	ITGB3	P05106	0.01330273
586	CRK	P46108	CYTH1	Q15438	0.01330273
587	IGF1	P05019	PDGFRB	P09619	0.01332741
588	APP	P05067	RIT2	Q99578	0.01332741
589	B2M	P61769	BLNK	Q8WV28	0.013401451
590	IGF1	P05019	ZAP70	P43403	0.013426132
591	HNRNPK	P61978	S100A6	P06703	0.013450812
592	B2M	P61769	GABRG3	Q99928	0.013475492

593	CRK	P46108	RALGPS2	Q86X27	0.013549534
594	ARCN1	P48444	PTK2	Q05397	0.013549534
595	CST3	P01034	EPHB2	P29323	0.013574214
596	CDH2	P19022	DLG4	P78352	0.013598894
597	CRK	P46108	RAB29	O14966	0.013672935
598	B2M	P61769	CD3G	P09693	0.013697616
599	HMOX1	P09601	F2R	P25116	0.013746977
600	B2M	P61769	GHR	P10912	0.013771657
601	CRK	P46108	CYTH3	O43739	0.013771657
602	APP	P05067	ARHGAP17	Q68EM7	0.013771657
603	IGF1	P05019	INPP5D	Q92835	0.013919739
604	IGFBP5	P24593	ROBO1	Q9Y6N7	0.01394442
605	IGFBP5	P24593	NF2	P35240	0.0139691
606	B2M	P61769	CD44	P16070	0.0139691
607	CRK	P46108	SKAP1	Q86WV1	0.014043141
608	HNRNPK	P61978	NTRK1	P04629	0.014067822
609	APP	P05067	CTNND1	O60716	0.014067822
610	APP	P05067	RAP1B	P61224	0.014117182
611	CST3	P01034	FRS2	Q8WU20	0.014141863
612	CST3	P01034	PSEN2	P49810	0.014166543
613	B2M	P61769	TNFRSF11A	Q9Y6Q6	0.014191224
614	CFL1	P23528	SMO	Q99835	0.014215904
615	CDH2	P19022	ITSN1	Q15811	0.014215904
616	IGF1	P05019	ITGA2	P17301	0.014240584
617	CDH2	P19022	GRIN2B	Q13224	0.014314626
618	IGF1	P05019	RHOA	P61586	0.014363986
619	CRK	P46108	NGEF	Q8N5V2	0.014438028
620	CST3	P01034	PTK2	Q05397	0.014462708
621	APP	P05067	RAB8B	Q92930	0.014536749
622	CRK	P46108	RAB27A	P51159	0.014561429
623	APP	P05067	RAB10	P61026	0.01461079
624	CST3	P01034	LCK	P06239	0.014635471
625	IGF1	P05019	RAC1	P63000	0.014660151
626	CRK	P46108	RHOJ	Q9H4E5	0.014660151
627	CST3	P01034	PSEN1	P49768	0.014684831
628	CRK	P46108	RAB40C	Q96S21	0.014709512
629	CFL1	P23528	GRIN2A	Q12879	0.014734192
630	IGF1	P05019	PRKCA	P17252	0.014734192
631	MAPRE1	Q15691	RAB11A	P62491	0.014734192
632	ARCN1	P48444	VAPA	Q9P0L0	0.014758873
633	PEA15	Q15121	TNF	P01375	0.014808233
634	IGFBP5	P24593	JAK2	O60674	0.014857594
635	SOD2	P04179	VEGFA	P15692	0.014882275

636	CRK	P46108	RASGRP1	O95267	0.014906955
637	S100A6	P06703	TNFRSF1A	P19438	0.014956316
638	CRK	P46108	IL6ST	P40189	0.014980996
639	CRK	P46108	SMAP1	Q8IYB5	0.015030357
640	CFL1	P23528	PREX1	Q8TCU6	0.015079718
641	B2M	P61769	MST1R	Q04912	0.015104398
642	IGF1	P05019	NOS3	P29474	0.015153759
643	CRK	P46108	RAB31	Q13636	0.015153759
644	B2M	P61769	SKAP1	Q86WV1	0.015277161
645	HMOX1	P09601	INPP5D	Q92835	0.015326522
646	CRK	P46108	SH2B2	O14492	0.015326522
647	CRK	P46108	RAB11B	Q15907	0.015351202
648	B2M	P61769	EPOR	P19235	0.015425243
649	CRK	P46108	INPP5D	Q92835	0.015449923
650	APP	P05067	AGER	Q15109	0.015449923
651	B2M	P61769	BAIAP2	Q9UQB8	0.015499284
652	HNRNPK	P61978	VAPA	Q9P0L0	0.015499284
653	HNRNPK	P61978	PSD3	Q9NYI0	0.015523965
654	MAPRE1	Q15691	RALA	P11233	0.015647367
655	CRK	P46108	RAB28	P51157	0.015696727
656	CST3	P01034	NF2	P35240	0.015820129
657	CDH2	P19022	FGFR1	P11362	0.01584481
658	CRK	P46108	CTNNAL1	Q9UBT7	0.01586949
659	CRK	P46108	RAB4A	P20338	0.01586949
660	CRK	P46108	NF2	P35240	0.01586949
661	PRDX5	P30044	UBE2B	P63146	0.01589417
662	CRK	P46108	FCGR2B	P31994	0.01589417
663	SOD1	P00441	TGFBR2	P37173	0.015968212
664	LGALS3	P17931	TNF	P01375	0.015992892
665	CDH2	P19022	FYN	P06241	0.016017572
666	B2M	P61769	ITGA2	P17301	0.016116294
667	CST3	P01034	RHOB	P62745	0.016190335
668	B2M	P61769	AGER	Q15109	0.016190335
669	SEC22B	O75396	UBE2B	P63146	0.016264376
670	APP	P05067	RAB3D	O95716	0.016264376
671	B2M	P61769	ADGRA2	Q96PE1	0.016289057
672	CNBP	P62633	RAB11A	P62491	0.016289057
673	SOD2	P04179	SPTBN1	Q01082	0.016313737
674	CRK	P46108	GJA1	P17302	0.016387778
675	SOD2	P04179	TNFRSF1A	P19438	0.016412459
676	B2M	P61769	UBE2B	P63146	0.016535861
677	CST3	P01034	FYN	P06241	0.016708623
678	CST3	P01034	RAC1	P63000	0.016757984

679	B2M	P61769	PLEKHA1	Q9HB21	0.017029468
680	CCL3	P10147	LCK	P06239	0.017054149
681	CRK	P46108	RAB30	Q15771	0.017054149
682	APP	P05067	TNFSF14	O43557	0.017054149
683	SOD1	P00441	VEGFA	P15692	0.01710351
684	HNRNPK	P61978	RAB18	Q9NP72	0.01710351
685	B2M	P61769	PREX1	Q8TCU6	0.01712819
686	CRK	P46108	RAB43	Q86YS6	0.017226911
687	LGALS1	P09382	LYN	P07948	0.017251592
688	HNRNPK	P61978	SPRED1	Q7Z699	0.017276272
689	HNRNPF	P52597	PRKCI	P41743	0.017374994
690	ARCN1	P48444	JAK2	O60674	0.017473715
691	CST3	P01034	IL6ST	P40189	0.017498396
692	ARCN1	P48444	RAC1	P63000	0.017498396
693	B2M	P61769	RASA2	Q15283	0.017621798
694	CRK	P46108	CSF1	P09603	0.017671158
695	CFL1	P23528	RAB14	P61106	0.017695839
696	MTPN	P58546	PDPK1	O15530	0.017695839
697	CST3	P01034	TNFRSF1A	P19438	0.0177452
698	CST3	P01034	ITGB1	P05556	0.01779456
699	CFL1	P23528	NOS3	P29474	0.017893282
700	APP	P05067	SKAP1	Q86WV1	0.017893282
701	B2M	P61769	CARD11	Q9BXL7	0.017917962
702	B2M	P61769	GAB2	Q9UQC2	0.018016684
703	HNRNPK	P61978	RALA	P11233	0.018016684
704	HNRNPK	P61978	RHOA	P61586	0.018016684
705	B2M	P61769	TLR6	Q9Y2C9	0.018164766
706	HNRNPK	P61978	HOMER2	Q9NSB8	0.018189447
707	CDH2	P19022	SIPA1L1	O43166	0.018214127
708	CRK	P46108	RHOC	P08134	0.018214127
709	PRDX6	P30041	RAC1	P63000	0.018263488
710	PRDX6	P30041	RAC1	P63000	0.018263488
711	CST3	P01034	ROBO1	Q9Y6N7	0.018288168
712	CST3	P01034	NEDD4	P46934	0.018288168
713	CRK	P46108	RASGRP2	Q7LDG7	0.018337529
714	B2M	P61769	LEPR	P48357	0.01838689
715	NME2	P22392	INPP5D	Q92835	0.018534972
716	HMOX1	P09601	LYN	P07948	0.018559653
717	HNRNPK	P61978	PREX1	Q8TCU6	0.018584333
718	APP	P05067	RAB11B	Q15907	0.018732415
719	CDH2	P19022	TNFRSF1A	P19438	0.018831137
720	APP	P05067	NCR1	O76036	0.0190039
721	B2M	P61769	STX2	P32856	0.01902858

722	B2M	P61769	FYN	P06241	0.01902858
723	CST3	P01034	PTPRF	P10586	0.019077941
724	CRK	P46108	RAB3D	O95716	0.019201343
725	B2M	P61769	ITGA4	P13612	0.019250703
726	APP	P05067	RAB14	P61106	0.019324745
727	CFL1	P23528	SIPA1L1	O43166	0.019349425
728	B2M	P61769	LY6E	Q16553	0.019472827
729	CRK	P46108	RAB23	Q9ULC3	0.019472827
730	APP	P05067	RAB35	Q15286	0.019546868
731	CFL1	P23528	SPTBN1	Q01082	0.019571548
732	B2M	P61769	ENG	P17813	0.019571548
733	MAPRE1	Q15691	TGFBR1	P36897	0.019620909
734	CRK	P46108	RALGPS1	Q5JS13	0.019620909
735	ARCN1	P48444	RHOA	P61586	0.01964559
736	CFL1	P23528	RAC1	P63000	0.01969495
737	B2M	P61769	IL1RL1	Q01638	0.019744311
738	CRK	P46108	RASGRP4	Q8TDF6	0.019768992
739	B2M	P61769	ARHGAP10	A1A4S6	0.019867713
740	CRK	P46108	CSK	P41240	0.019966435
741	CRK	P46108	GEM	P55040	0.020213239
742	APP	P05067	PIP5K1A	Q99755	0.020213239
743	CST3	P01034	RHOA	P61586	0.020237919
744	B2M	P61769	LIFR	P42702	0.020262599
745	IGFBP2	P18065	NEDD4	P46934	0.02028728
746	HNRNPK	P61978	RAP1B	P61224	0.02031196
747	APP	P05067	G3BP1	Q13283	0.020336641
748	CRK	P46108	DGKZ	Q13574	0.020410682
749	LTBP2	Q14767	NEDD4	P46934	0.020484723
750	CFL1	P23528	ITGA2	P17301	0.020632805
751	CRK	P46108	RHOQ	P17081	0.020632805
752	GFAP	P14136	PRKCA	P17252	0.020731527
753	APP	P05067	RALGPS1	Q5JS13	0.020731527
754	CRK	P46108	PREX1	Q8TCU6	0.020756207
755	CCL3	P10147	JAK2	O60674	0.020805568
756	B2M	P61769	GRB14	Q14449	0.020830248
757	B2M	P61769	UNC5C	O95185	0.020854929
758	CRK	P46108	SLA2	Q9H6Q3	0.020854929
759	CRK	P46108	TACR1	P25103	0.020854929
760	B2M	P61769	SPN	P16150	0.020904289
761	B2M	P61769	JAK2	O60674	0.02095365
762	CRK	P46108	CRK	P46108	0.021003011
763	APP	P05067	RASL10B	Q96S79	0.021027691
764	CFL1	P23528	SHH	Q15465	0.021101733

765	CRK	P46108	NEDD4	P46934	0.021200454
766	GAA	P10253	NTRK1	P04629	0.021200454
767	IGFBP5	P24593	ITGA4	P13612	0.021225135
768	B2M	P61769	EDA2R	Q9HAV5	0.021274495
769	HMOX1	P09601	TNF	P01375	0.021422578
770	IGFBP2	P18065	LCK	P06239	0.021471938
771	CTGF	P29279	TGFBR1	P36897	0.02154598
772	CRK	P46108	SPTBN1	Q01082	0.021620021
773	CRK	P46108	RAB40B	Q12829	0.021620021
774	HMOX1	P09601	JAK2	O60674	0.021768103
775	APP	P05067	RAB40B	Q12829	0.021817464
776	CFL1	P23528	FYN	P06241	0.022014907
777	CRK	P46108	PLD2	O14939	0.022014907
778	SOD1	P00441	SORBS1	Q9BX66	0.022039587
779	IGFBP5	P24593	LYN	P07948	0.022064268
780	B2M	P61769	CSF1	P09603	0.022064268
781	LGALS3	P17931	TGFBR1	P36897	0.022113629
782	APP	P05067	GABRA4	P48169	0.02221235
783	CDH2	P19022	PKD1	P98161	0.022335752
784	B2M	P61769	LYN	P07948	0.022335752
785	HNRNPK	P61978	LCK	P06239	0.022335752
786	HMOX1	P09601	SH2B2	O14492	0.022385113
787	ARCN1	P48444	SPTBN1	Q01082	0.022582556
788	CRK	P46108	DIRAS1	O95057	0.022779999
789	CRK	P46108	MRAS	O14807	0.02282936
790	LTBP2	Q14767	TGFBR1	P36897	0.023002123
791	CRK	P46108	RGS19	P49795	0.023051483
792	B2M	P61769	TNFRSF19	Q9NS68	0.023248926
793	CRK	P46108	RASA4	O43374	0.023248926
794	B2M	P61769	BTK	Q06187	0.023347648
795	IGFBP5	P24593	NEDD4	P46934	0.02347105
796	B2M	P61769	ADGRA3	Q8IWK6	0.023594452
797	MAPRE1	Q15691	NF2	P35240	0.023619132
798	CRK	P46108	SPRED2	Q7Z698	0.023668493
799	B2M	P61769	IL9R	Q01113	0.023693173
800	APP	P05067	VAPA	Q9P0L0	0.023767215
801	B2M	P61769	DLL4	Q9NR61	0.023841256
802	CDH2	P19022	RAB5A	P20339	0.023890617
803	SOD1	P00441	ZAP70	P43403	0.023915297
804	B2M	P61769	CLEC1B	Q9P126	0.023964658
805	HNRNPK	P61978	RAB5A	P20339	0.024038699
806	APP	P05067	RAB39A	Q14964	0.02408806
807	APP	P05067	RAB38	P57729	0.02411274

808	APP	P05067	RAB28	P51157	0.02413742
809	IGFBP5	P24593	SPTBN1	Q01082	0.024186781
810	HNRNPK	P61978	RAB14	P61106	0.024186781
811	IGFBP2	P18065	JAK2	O60674	0.024310183
812	NME2	P22392	TNF	P01375	0.024359544
813	CTGF	P29279	PRKCA	P17252	0.024482946
814	HNRNPF	P52597	ITGA4	P13612	0.024482946
815	ARCN1	P48444	TGFBR1	P36897	0.024507626
816	ARCN1	P48444	NTRK1	P04629	0.024606348
817	CRK	P46108	GOLPH3	Q9H4A6	0.02475443
818	CRK	P46108	RASL10B	Q96S79	0.024828471
819	CRK	P46108	PLEK2	Q9NYT0	0.024927193
820	IGFBP2	P18065	ILK	Q13418	0.024951873
821	CNBP	P62633	UBE2B	P63146	0.024951873
822	APP	P05067	SMAP1	Q8IYB5	0.025001234
823	SOD1	P00441	TGFBR1	P36897	0.025124636
824	CTGF	P29279	FYN	P06241	0.025198677
825	CRK	P46108	IKBKB	O14920	0.025223358
826	CRK	P46108	RAB3C	Q96E17	0.025297399
827	B2M	P61769	TNFRSF1A	P19438	0.02539612
828	CFL1	P23528	ITGA4	P13612	0.025420801
829	CRK	P46108	VAPA	Q9P0L0	0.025544203
830	APP	P05067	PRKCA	P17252	0.025716965
831	CRK	P46108	RAB39A	Q14964	0.025791006
832	APP	P05067	ARRB1	P49407	0.02601313
833	B2M	P61769	ITGB2	P05107	0.026062491
834	CRK	P46108	USP8	P40818	0.026062491
835	APP	P05067	IL1RL1	Q01638	0.026185893
836	CRK	P46108	RAB39B	Q96DA2	0.026284614
837	LGALS1	P09382	RHOA	P61586	0.026333975
838	CCL3	P10147	SH2B2	O14492	0.026482057
839	HP	P00738	JAK2	O60674	0.026482057
840	HNRNPK	P61978	RAB11A	P62491	0.026506738
841	B2M	P61769	IL2RB	P14784	0.026802902
842	B2M	P61769	GLRB	P48167	0.027123747
843	CRK	P46108	PSD3	Q9NYI0	0.027148428
844	APP	P05067	GPR61	Q9BZJ8	0.027148428
845	CRK	P46108	SCRIB	Q14160	0.027173108
846	IGF1	P05019	SHH	Q15465	0.027345871
847	APP	P05067	VEGFA	P15692	0.027419912
848	LTBP2	Q14767	SPTBN1	Q01082	0.027469273
849	GSTM2	P28161	SPTBN1	Q01082	0.027790118
850	GSTM2	P28161	SPTBN1	Q01082	0.027790118

851	APP	P05067	LANCL2	Q9NS86	0.027790118
852	CRK	P46108	LAT	O43561	0.027839479
853	APP	P05067	DIRAS1	O95057	0.027864159
854	B2M	P61769	RAB9A	P51151	0.028086283
855	CST3	P01034	TGFBR1	P36897	0.028160324
856	B2M	P61769	DAPP1	Q9UN19	0.028185004
857	SOD2	P04179	TGFBR1	P36897	0.028209685
858	MAPRE1	Q15691	PSEN1	P49768	0.028234365
859	APP	P05067	SPTBN1	Q01082	0.028234365
860	MTPN	P58546	RHOA	P61586	0.028333087
861	B2M	P61769	RIT1	Q92963	0.02855521
862	CRK	P46108	FGD2	Q7Z6J4	0.028629251
863	APP	P05067	HOMER1	Q86YM7	0.028629251
864	APP	P05067	GRIA3	P42263	0.028703292
865	IGF1	P05019	TGFBR1	P36897	0.028777334
866	CST3	P01034	PRKCA	P17252	0.028826694
867	B2M	P61769	P2RX7	Q99572	0.028876055
868	B2M	P61769	CNTNAP2	Q9UHC6	0.029048818
869	B2M	P61769	TGFBR3	Q03167	0.029320302
870	B2M	P61769	RASGRF2	O14827	0.029369663
871	B2M	P61769	GABRA4	P48169	0.029419024
872	CRK	P46108	RHOF	Q9HBH0	0.029567106
873	B2M	P61769	GABRB2	P47870	0.029591786
874	B2M	P61769	SDCBP	O00560	0.029591786
875	B2M	P61769	SELE	P16581	0.029616467
876	CCL3	P10147	LYN	P07948	0.029739869
877	CRK	P46108	LEPR	P48357	0.029912631
878	CRK	P46108	RASGRF2	O14827	0.029961992
879	CRK	P46108	RND1	Q92730	0.030134755
880	APP	P05067	PTK2B	Q14289	0.030282837
881	CRK	P46108	PRKN	O60260	0.0304556
882	CRK	P46108	GPR65	Q8IYL9	0.030628363
883	CDH2	P19022	SORBS1	Q9BX66	0.030677723
884	CRK	P46108	PIK3C2A	O00443	0.030677723
885	B2M	P61769	VAPA	Q9P0L0	0.030776445
886	CDH2	P19022	LCK	P06239	0.030924527
887	B2M	P61769	FGFR1	P11362	0.031146651
888	HP	P00738	LYN	P07948	0.031270053
889	CRK	P46108	RAB3A	P20336	0.031418135
890	B2M	P61769	TLR1	Q15399	0.031541537
891	CRK	P46108	RAB17	Q9H0T7	0.031590898
892	APP	P05067	CDH2	P19022	0.03173898
893	CRK	P46108	DCBLD2	Q96PD2	0.031763661

894	APP	P05067	ADAP2	Q9NPF8	0.031788341
895	APP	P05067	ERBB2	P04626	0.031837702
896	APP	P05067	ARF6	P62330	0.032578113
897	IGFBP2	P18065	LYN	P07948	0.032726196
898	CDH2	P19022	INPP5D	Q92835	0.032800237
899	APP	P05067	FYN	P06241	0.032849598
900	B2M	P61769	EFNA1	P20827	0.032923639
901	HNRNPK	P61978	SDCBP	O00560	0.032923639
902	CRK	P46108	RAB33A	Q14088	0.03302236
903	B2M	P61769	ITGA3	P26006	0.033047041
904	APP	P05067	GRIN2B	Q13224	0.033071721
905	CRK	P46108	RIT2	Q99578	0.033244484
906	CRK	P46108	RAB38	P57729	0.033269164
907	APP	P05067	TNFSF13B	Q9Y275	0.03361469
908	CRK	P46108	RAB19	A4D1S5	0.03363937
909	APP	P05067	CHRNA1	P02708	0.033664051
910	CRK	P46108	PTPRF	P10586	0.033787452
911	HNRNPK	P61978	TGFBR1	P36897	0.033787452
912	CRK	P46108	UBE2B	P63146	0.033960215
913	CRK	P46108	ILK	Q13418	0.034207019
914	B2M	P61769	GABRQ	Q9UN88	0.03428106
915	LGALS3	P17931	LYN	P07948	0.034305741
916	B2M	P61769	TLR7	Q9NYK1	0.034330421
917	APP	P05067	ITPR1	Q14643	0.034651266
918	APP	P05067	EPS15	P42566	0.034700627
919	IGFBP2	P18065	PRKCA	P17252	0.034774668
920	CRK	P46108	ITGB1	P05556	0.034799348
921	B2M	P61769	GABRA2	P47869	0.034972111
922	B2M	P61769	NGFR	P08138	0.034996792
923	B2M	P61769	GABRD	O14764	0.035070833
924	APP	P05067	CD36	P16671	0.035070833
925	CRK	P46108	L1CAM	P32004	0.035589121
926	B2M	P61769	GABRA1	P14867	0.036033368
927	CDH2	P19022	SH2B2	O14492	0.036255491
928	CST3	P01034	JAK2	O60674	0.036304852
929	APP	P05067	DRD1	P21728	0.036378893
930	IGFBP5	P24593	TGFBR1	P36897	0.036502295
931	CRK	P46108	RHOD	O00212	0.036872501
932	B2M	P61769	CD244	Q9BZW8	0.036921862
933	HNRNPK	P61978	SPTBN1	Q01082	0.036921862
934	B2M	P61769	GABRR1	P24046	0.036995903
935	B2M	P61769	CD14	P08571	0.037069944
936	CRK	P46108	TGFBR1	P36897	0.037069944

937	APP	P05067	GRIA4	P48058	0.037069944
938	APP	P05067	STOML3	Q8TAV4	0.037119305
939	CFL1	P23528	INPP5D	Q92835	0.037143985
940	CRK	P46108	VEGFA	P15692	0.037168666
941	CFL1	P23528	LYN	P07948	0.037193346
942	B2M	P61769	GABRA5	P31644	0.037218027
943	B2M	P61769	GP6	Q9HCN6	0.037316748
944	APP	P05067	CDH13	P55290	0.037390789
945	CDH2	P19022	RHOA	P61586	0.03746483
946	B2M	P61769	BCAM	P50895	0.037514191
947	B2M	P61769	CDH2	P19022	0.037588232
948	APP	P05067	VIPR2	P41587	0.037637593
949	APP	P05067	CHRNB2	P17787	0.037662274
950	APP	P05067	ROBO1	Q9Y6N7	0.038007799
951	IGFBP2	P18065	TGFBR1	P36897	0.03805716
952	CRK	P46108	MAST1	Q9Y2H9	0.038106521
953	APP	P05067	GPR3	P46089	0.038106521
954	B2M	P61769	VAPB	O95292	0.038229923
955	CRK	P46108	SPRED1	Q7Z699	0.038303964
956	APP	P05067	F2R	P25116	0.03867417
957	CRK	P46108	RRAD	P55042	0.038822252
958	B2M	P61769	CD3D	P04234	0.039044375
959	B2M	P61769	GABRB3	P28472	0.039291179
960	CRK	P46108	GRIN2B	Q13224	0.03931586
961	MTPN	P58546	TGFBR1	P36897	0.03936522
962	B2M	P61769	GABRG1	Q8N1C3	0.039537983
963	CRK	P46108	SIPA1L1	O43166	0.039587344
964	APP	P05067	PTPRF	P10586	0.039908189
965	CRK	P46108	LCK	P06239	0.03998223
966	APP	P05067	S1PR1	P21453	0.040006911
967	CRK	P46108	RASL10A	Q92737	0.040105632
968	CRK	P46108	RGS9	O75916	0.040130312
969	B2M	P61769	ITGAV	P06756	0.040204354
970	APP	P05067	EPHB2	P29323	0.040204354
971	APP	P05067	DRD2	P14416	0.040253714
972	B2M	P61769	GLRA1	P23415	0.040352436
973	APP	P05067	RAB11A	P62491	0.040352436
974	APP	P05067	MCF2L	O15068	0.040549879
975	CRK	P46108	CTNND1	O60716	0.040772003
976	B2M	P61769	CD247	P20963	0.040870724
977	APP	P05067	ARAP3	Q8WWN8	0.040870724
978	B2M	P61769	CELSR1	Q9NYQ6	0.040895405
979	APP	P05067	ACVR2A	P27037	0.040994126

980	CRK	P46108	EFNA1	P20827	0.041364332
981	CRK	P46108	TRAF4	Q9BUZ4	0.041413693
982	CRK	P46108	F2R	P25116	0.041734538
983	CRK	P46108	TNFRSF1A	P19438	0.041833259
984	CRK	P46108	PSEN2	P49810	0.0419073
985	CRK	P46108	GRASP	Q7Z6J2	0.0419073
986	CRK	P46108	RAB25	P57735	0.041956661
987	CRK	P46108	RAB26	Q9ULW5	0.042055383
988	B2M	P61769	RAB29	O14966	0.042154104
989	B2M	P61769	SORBS1	Q9BX66	0.042252826
990	APP	P05067	CNR1	P21554	0.042351547
991	CRK	P46108	PSD4	Q8NDX1	0.042376228
992	APP	P05067	NOS3	P29474	0.04252431
993	CRK	P46108	TNFRSF11A	Q9Y6Q6	0.042697073
994	B2M	P61769	PTK2B	Q14289	0.042721753
995	B2M	P61769	GABRA3	P34903	0.042771114
996	APP	P05067	JAK2	O60674	0.042968557
997	B2M	P61769	GABRG2	P18507	0.042993238
998	APP	P05067	EPHA4	P54764	0.043017918
999	CRK	P46108	RYK	P34925	0.043067279
1000	APP	P05067	RND1	Q92730	0.04314132
1001	B2M	P61769	GABRR2	P28476	0.043190681
1002	CRK	P46108	LANCL2	Q9NS86	0.043240041
1003	B2M	P61769	EDA	Q92838	0.043314083
1004	CRK	P46108	IFNAR2	P48551	0.043437485
1005	APP	P05067	TNF	P01375	0.043511526
1006	ARCN1	P48444	PRKCA	P17252	0.043536206
1007	APP	P05067	CNTNAP2	Q9UHC6	0.043536206
1008	CRK	P46108	DAPP1	Q9UN19	0.043585567
1009	APP	P05067	SSTR2	P30874	0.043634928
1010	APP	P05067	MS4A2	Q01362	0.043857051
1011	B2M	P61769	FZD6	O60353	0.043980453
1012	CRK	P46108	RHOV	Q96L33	0.044005134
1013	APP	P05067	PLPP1	O14494	0.044054494
1014	APP	P05067	SIPA1L1	O43166	0.044103855
1015	CRK	P46108	RGS14	O43566	0.044128535
1016	APP	P05067	KIT	P10721	0.044153216
1017	APP	P05067	CHRNA3	P32297	0.04440002
1018	APP	P05067	ILK	Q13418	0.04440002
1019	CRK	P46108	ROBO1	Q9Y6N7	0.044646824
1020	APP	P05067	THY1	P04216	0.044720865
1021	APP	P05067	BRS3	P32247	0.044967669
1022	CRK	P46108	SOCS7	O14512	0.044992349

1023	B2M	P61769	DCBLD2	Q96PD2	0.045140431
1024	CRK	P46108	KL	Q9UEF7	0.045189792
1025	APP	P05067	STX2	P32856	0.045214473
1026	B2M	P61769	RHOQ	P17081	0.045263833
1027	APP	P05067	HBEGF	Q99075	0.045411916
1028	B2M	P61769	C3AR1	Q16581	0.045609359
1029	CRK	P46108	RASD2	Q96D21	0.04565872
1030	APP	P05067	GABRA2	P47869	0.04565872
1031	APP	P05067	NF2	P35240	0.045905523
1032	B2M	P61769	TGFBR2	P37173	0.045979565
1033	APP	P05067	GABRA3	P34903	0.045979565
1034	CRK	P46108	JAK2	O60674	0.046004245
1035	CDH2	P19022	JAK2	O60674	0.046053606
1036	SEC22B	O75396	TGFBR1	P36897	0.046053606
1037	APP	P05067	GRIN2A	Q12879	0.046201688
1038	B2M	P61769	CELSR2	Q9HCU4	0.046423812
1039	CRK	P46108	IL6R	P08887	0.046473172
1040	APP	P05067	CHRND	Q07001	0.046547214
1041	APP	P05067	ITSN1	Q15811	0.046571894
1042	CRK	P46108	LPAR1	Q92633	0.046621255
1043	APP	P05067	CHRNB1	P11230	0.046670616
1044	APP	P05067	GRIN3B	O60391	0.046695296
1045	APP	P05067	PARD3	Q8TEW0	0.046843378
1046	APP	P05067	FGFR1	P11362	0.046868059
1047	APP	P05067	ACVR1	Q04771	0.047016141
1048	APP	P05067	ADRB3	P13945	0.047065502
1049	APP	P05067	RHOA	P61586	0.047213584
1050	APP	P05067	ALCAM	Q13740	0.047805913
1051	CRK	P46108	DTNA	Q9Y4J8	0.047953996
1052	APP	P05067	GRIK2	Q13002	0.048077398
1053	CRK	P46108	RHEBL1	Q8TAI7	0.048151439
1054	APP	P05067	L1CAM	P32004	0.048176119
1055	CRK	P46108	PRKCA	P17252	0.048299521
1056	B2M	P61769	RAB10	P61026	0.048422923
1057	PEA15	Q15121	TGFBR1	P36897	0.048472284
1058	CRK	P46108	RAB9B	Q9NP90	0.048472284
1059	APP	P05067	GRIK1	P39086	0.048595686
1060	APP	P05067	GRID2	O43424	0.048694407
1061	APP	P05067	CELSR3	Q9NYQ7	0.04884249
1062	APP	P05067	CELSR2	Q9HCU4	0.04886717
1063	APP	P05067	GLRA1	P23415	0.048891851
1064	APP	P05067	FRS2	Q8WU20	0.049188015
1065	APP	P05067	PDGFRA	P16234	0.049212696

1066	CRK	P46108	ATP6AP2	O75787	0.049360778
1067	APP	P05067	PHLDA3	Q9Y5J5	0.049607582
1068	APP	P05067	GABRA5	P31644	0.050224592
1069	APP	P05067	HCRTR2	O43614	0.050224592
1070	APP	P05067	NPY5R	Q15761	0.050224592
1071	LGALS1	P09382	TGFBR1	P36897	0.050273952
1072	APP	P05067	AGTR1	P30556	0.050298633
1073	CFL1	P23528	JAK2	O60674	0.050347993
1074	APP	P05067	IL6ST	P40189	0.050422035
1075	APP	P05067	RASGRF2	O14827	0.050471395
1076	CRK	P46108	PLEKHA1	Q9HB21	0.05079224
1077	B2M	P61769	ILK	Q13418	0.050940323
1078	APP	P05067	GHR	P10912	0.051211807
1079	APP	P05067	TACR1	P25103	0.051582013
1080	APP	P05067	RHOQ	P17081	0.051631374
1081	APP	P05067	S100A6	P06703	0.051656054
1082	APP	P05067	SH2B2	O14492	0.051779456
1083	APP	P05067	TSHR	P16473	0.051804136
1084	CRK	P46108	GNA11	P29992	0.05202626
1085	CRK	P46108	RGS6	P49758	0.052248383
1086	CRK	P46108	SMO	Q99835	0.052396466
1087	APP	P05067	GALR2	O43603	0.052519868
1088	APP	P05067	SHH	Q15465	0.052791352
1089	APP	P05067	EGF	P01133	0.052865393
1090	LXN	Q9BS40	TGFBR1	P36897	0.053359001
1091	APP	P05067	GABBR1	Q9UBS5	0.053433042
1092	APP	P05067	GRIK4	Q16099	0.053507083
1093	APP	P05067	ARHGEF25	Q86VW2	0.053556444
1094	APP	P05067	CRK	P46108	0.05395133
1095	B2M	P61769	NEDD4	P46934	0.053976011
1096	APP	P05067	ADORA2A	P29274	0.054099413
1097	B2M	P61769	SOCS7	O14512	0.054222815
1098	APP	P05067	TGFBR1	P36897	0.054222815
1099	APP	P05067	UNC5B	Q8IZJ1	0.05459302
1100	CRK	P46108	SMURF2	Q9HAU4	0.054642381
1101	APP	P05067	HRH3	Q9Y5N1	0.054691742
1102	B2M	P61769	RHOC	P08134	0.054765783
1103	APP	P05067	CCKAR	P32238	0.054815144
1104	APP	P05067	CSF1	P09603	0.054938546
1105	APP	P05067	TMPRSS6	Q8IU80	0.055086628
1106	APP	P05067	CD44	P16070	0.055111309
1107	CRK	P46108	RGS17	Q9UGC6	0.055358112
1108	CRK	P46108	GRB14	Q14449	0.055456834

1109	CRK	P46108	TGFBR2	P37173	0.055752999
1110	CRK	P46108	MAGI3	Q5TCQ9	0.055802359
1111	APP	P05067	FGD2	Q7Z6J4	0.05585172
1112	APP	P05067	PTGER2	P43116	0.055999803
1113	CRK	P46108	MARK2	Q7KZI7	0.056073844
1114	APP	P05067	ADRB1	P08588	0.056295967
1115	APP	P05067	PTPRE	P23469	0.056394689
1116	CRK	P46108	ENG	P17813	0.056419369
1117	APP	P05067	GABRB3	P28472	0.057061059
1118	APP	P05067	HRH1	P35367	0.057233822
1119	APP	P05067	CHRNB4	P30926	0.057431265
1120	CRK	P46108	PTPRC	P08575	0.057628708
1121	APP	P05067	SLC7A1	P30825	0.05775211
1122	APP	P05067	BMPR2	Q13873	0.057776791
1123	APP	P05067	CD4	P01730	0.057924873
1124	APP	P05067	P2RY1	P47900	0.057949553
1125	CRK	P46108	NCAM1	P13591	0.058097636
1126	APP	P05067	NPY1R	P25929	0.058146996
1127	APP	P05067	CHRNA5	P30532	0.058221038
1128	APP	P05067	EPHA7	Q15375	0.058270398
1129	CRK	P46108	CXCR4	P61073	0.058443161
1130	APP	P05067	AVPR1A	P37288	0.058492522
1131	APP	P05067	ADORA1	P30542	0.059306975
1132	APP	P05067	TRIP10	Q15642	0.059455057
1133	APP	P05067	GRIK3	Q13003	0.059504418
1134	APP	P05067	PKD1	P98161	0.059701861
1135	APP	P05067	GRIA1	P42261	0.059726541
1136	APP	P05067	ERBB3	P21860	0.059973345
1137	APP	P05067	UNC5C	O95185	0.059998026
1138	CRK	P46108	PKN1	Q16512	0.060590355
1139	CRK	P46108	CD74	P04233	0.060689076
1140	APP	P05067	DNER	Q8NFT8	0.060763118
1141	CRK	P46108	TEK	Q02763	0.0609112
1142	APP	P05067	RAB5A	P20339	0.060985241
1143	APP	P05067	UNC5A	Q6ZN44	0.061182684
1144	APP	P05067	ARHGAP10	A1A4S6	0.061306086
1145	APP	P05067	CX3CR1	P49238	0.061478849
1146	B2M	P61769	RAB5A	P20339	0.06157757
1147	APP	P05067	OPRD1	P41143	0.061725653
1148	APP	P05067	NEDD4	P46934	0.062441384
1149	APP	P05067	F2RL1	P55085	0.062490745
1150	APP	P05067	JAG1	P78504	0.062564786
1151	APP	P05067	CHRM3	P20309	0.062663508

1152	APP	P05067	KITLG	P21583	0.06281159
1153	APP	P05067	FASLG	P48023	0.062934992
1154	CRK	P46108	CD40	P25942	0.063083074
1155	APP	P05067	CAP1	Q01518	0.063107755
1156	APP	P05067	GHRHR	Q02643	0.063280517
1157	APP	P05067	TDGF1	P13385	0.063305198
1158	APP	P05067	INSR	P06213	0.063379239
1159	APP	P05067	CCR4	P51679	0.063403919
1160	APP	P05067	ITGA1	P56199	0.063749445
1161	APP	P05067	CCKBR	P32239	0.063798805
1162	CFL1	P23528	TGFBR1	P36897	0.063872847
1163	APP	P05067	LEPR	P48357	0.063922207
1164	APP	P05067	HCRTR1	O43613	0.063996249
1165	APP	P05067	EPHB1	P54762	0.064193692
1166	APP	P05067	BDKRB2	P30411	0.064341774
1167	APP	P05067	RAB39B	Q96DA2	0.064415815
1168	APP	P05067	PLEKHA1	Q9HB21	0.064440496
1169	APP	P05067	GRB14	Q14449	0.064662619
1170	APP	P05067	IHH	Q14623	0.064860062
1171	APP	P05067	SOCS7	O14512	0.065279629
1172	APP	P05067	PREX1	Q8TCU6	0.065279629
1173	APP	P05067	KDR	P35968	0.065452392
1174	APP	P05067	BDKRB1	P46663	0.065575793
1175	B2M	P61769	RAC1	P63000	0.065773237
1176	B2M	P61769	TGFBR1	P36897	0.066316205
1177	APP	P05067	ADRA2A	P08913	0.066316205
1178	B2M	P61769	PRKCA	P17252	0.06661237
1179	APP	P05067	TRAF4	Q9BUZ4	0.066661731
1180	APP	P05067	ITGA8	P53708	0.066809813
1181	APP	P05067	C3AR1	Q16581	0.067056617
1182	APP	P05067	RAB22A	Q9UL26	0.067105978
1183	APP	P05067	FZD9	O00144	0.067994472
1184	APP	P05067	PDE6C	P51160	0.068685522
1185	APP	P05067	NGEF	Q8N5V2	0.069080409
1186	APP	P05067	ADRA2C	P18825	0.069228491
1187	APP	P05067	DLL4	Q9NR61	0.069425934
1188	APP	P05067	FZD5	Q13467	0.069697418
1189	APP	P05067	CD28	P10747	0.06977146
1190	APP	P05067	BCL10	O95999	0.070018263
1191	APP	P05067	PTPRC	P08575	0.070191026
1192	APP	P05067	ENG	P17813	0.07041315
1193	APP	P05067	ADCY6	O43306	0.07043783
1194	APP	P05067	CD8A	P01732	0.070659954

1195	APP	P05067	SDCBP	O00560	0.070857397
1196	APP	P05067	LCK	P06239	0.071178242
1197	APP	P05067	MIB1	Q86YT6	0.071351004
1198	APP	P05067	CYSLTR1	Q9Y271	0.071770571
1199	APP	P05067	EPHB3	P54753	0.072116097
1200	APP	P05067	CCR10	P46092	0.072214818
1201	APP	P05067	GABRB2	P47870	0.072510983
1202	APP	P05067	SHC4	Q6S5L8	0.072609704
1203	APP	P05067	RHOU	Q7L0Q8	0.072733106
1204	APP	P05067	MC4R	P32245	0.073029271
1205	APP	P05067	CNKSR2	Q8WXI2	0.073399477
1206	APP	P05067	LPAR1	Q92633	0.073448838
1207	APP	P05067	TNFRSF1A	P19438	0.073572239
1208	APP	P05067	RALA	P11233	0.073942445
1209	APP	P05067	ITGA2	P17301	0.074016486
1210	APP	P05067	RGS6	P49758	0.074287971
1211	APP	P05067	KCNIP3	Q9Y2W7	0.074534775
1212	APP	P05067	LHCGR	P22888	0.074707537
1213	APP	P05067	RXFP2	Q8WXD0	0.074979022
1214	APP	P05067	ADRB2	P07550	0.075053063
1215	APP	P05067	MRAS	O14807	0.075151784
1216	APP	P05067	XCR1	P46094	0.075225826
1217	APP	P05067	ACVR1B	P36896	0.07611432
1218	APP	P05067	PATJ	Q8NI35	0.076262402
1219	APP	P05067	DCBLD2	Q96PD2	0.076361123
1220	APP	P05067	FZD2	Q14332	0.076484525
1221	APP	P05067	RYK	P34925	0.076583247
1222	APP	P05067	CCR7	P32248	0.076731329
1223	APP	P05067	TRPV4	Q9HBA0	0.077002814
1224	APP	P05067	PLXNB1	O43157	0.07742238
1225	APP	P05067	KCNH2	Q12809	0.078508317
1226	APP	P05067	CCR1	P32246	0.078582358
1227	APP	P05067	CXCR3	P49682	0.078755121
1228	APP	P05067	ELMO1	Q92556	0.078779802
1229	APP	P05067	SMO	Q99835	0.078952564
1230	APP	P05067	GABRD	O14764	0.079125327
1231	APP	P05067	OR2A7	Q96R45	0.07932277
1232	APP	P05067	GABRR2	P28476	0.079520213
1233	APP	P05067	SEMA4C	Q9C0C4	0.079742337
1234	APP	P05067	CHRNG	P07510	0.079915099
1235	APP	P05067	PKN1	Q16512	0.080384027
1236	APP	P05067	CHRNA4	P43681	0.080408707
1237	APP	P05067	ROCK2	O75116	0.080408707

1238	APP	P05067	LRP12	Q9Y561	0.080458068
1239	APP	P05067	RGS17	Q9UGC6	0.080729552
1240	APP	P05067	INPP5D	Q92835	0.081173799
1241	APP	P05067	LYN	P07948	0.08122316
1242	APP	P05067	FAS	P25445	0.081346562
1243	APP	P05067	ITGA5	P08648	0.081346562
1244	APP	P05067	DGKQ	P52824	0.081667407
1245	APP	P05067	GABRG3	Q99928	0.081790809
1246	APP	P05067	PDGFRB	P09619	0.082210376
1247	APP	P05067	RAB40C	Q96S21	0.08250654
1248	APP	P05067	NMUR1	Q9HB89	0.082580581
1249	APP	P05067	WWP1	Q9H0M0	0.082876746
1250	APP	P05067	RHOJ	Q9H4E5	0.082950787
1251	APP	P05067	ITPR3	Q14573	0.082975468
1252	APP	P05067	ARHGEF28	Q8N1W1	0.083320993
1253	APP	P05067	ADCY9	O60503	0.084234168
1254	APP	P05067	GABRG1	Q8N1C3	0.084283528
1255	APP	P05067	ADCY2	Q08462	0.084431611
1256	APP	P05067	USP8	P40818	0.084480971
1257	APP	P05067	BSG	P35613	0.084826497
1258	APP	P05067	DLL1	O00548	0.085320105
1259	APP	P05067	ITGB1	P05556	0.08566563
1260	APP	P05067	SLA2	Q9H6Q3	0.08566563
1261	APP	P05067	CCR9	P51686	0.08569031
1262	APP	P05067	EPOR	P19235	0.085937114
1263	APP	P05067	CCR8	P51685	0.085937114
1264	APP	P05067	GPR1	P46091	0.085986475
1265	APP	P05067	PTGDR2	Q9Y5Y4	0.086381361
1266	APP	P05067	CHRNB3	Q05901	0.086430722
1267	APP	P05067	DGKZ	Q13574	0.086480083
1268	APP	P05067	RASD2	Q96D21	0.086652846
1269	APP	P05067	JAG2	Q9Y219	0.086677526
1270	APP	P05067	CCR2	P41597	0.087245175
1271	APP	P05067	CALCR	P30988	0.087245175
1272	APP	P05067	TGFBR3	Q03167	0.087368577
1273	APP	P05067	DAPP1	Q9UN19	0.087442618
1274	APP	P05067	NCAM1	P13591	0.087689422
1275	APP	P05067	FGFR3	P22607	0.088355792
1276	APP	P05067	MYO6	Q9UM54	0.088355792
1277	APP	P05067	SPRED1	Q7Z699	0.088380473
1278	APP	P05067	RGS19	P49795	0.088775359
1279	APP	P05067	RGS20	O76081	0.08882472
1280	APP	P05067	FLT1	P17948	0.088898761

1281	APP	P05067	EPHA3	P29320	0.089145565
1282	APP	P05067	FZD6	O60353	0.089219606
1283	APP	P05067	RGR	P47804	0.089960018
1284	APP	P05067	ADCYAP1R1	P41586	0.090009379
1285	APP	P05067	CACNA1A	O00555	0.090428945
1286	APP	P05067	DOK3	Q7L591	0.090971914
1287	APP	P05067	GRM3	Q14832	0.091119996
1288	APP	P05067	TNS2	Q63HR2	0.091169357
1289	APP	P05067	CCR6	P51684	0.091811047
1290	APP	P05067	ITGB4	P16144	0.091909768
1291	APP	P05067	ADCY4	Q8NFM4	0.091934449
1292	APP	P05067	CXCR4	P61073	0.092057851
1293	APP	P05067	NOD1	Q9Y239	0.092304655
1294	APP	P05067	LY6E	Q16553	0.092403376
1295	APP	P05067	ACVRL1	P37023	0.092576139
1296	APP	P05067	S1PR3	Q99500	0.093785478
1297	APP	P05067	S1PR3	Q99500	0.093785478
1298	APP	P05067	ADCY7	P51828	0.094056962
1299	APP	P05067	RAB31	Q13636	0.094254405
1300	APP	P05067	CHRNA2	Q15822	0.094402488
1301	APP	P05067	NTRK1	P04629	0.094797374
1302	APP	P05067	SLC9A3R1	O14745	0.095290982
1303	APP	P05067	DTNA	Q9Y4J8	0.095315662
1304	APP	P05067	PTPRT	O14522	0.095735229
1305	APP	P05067	GPC3	P51654	0.095932672
1306	APP	P05067	IL6R	P08887	0.096130115
1307	APP	P05067	CALCRL	Q16602	0.096500321
1308	APP	P05067	CD38	P28907	0.096747125
1309	APP	P05067	NPFFR2	Q9Y5X5	0.096796486
1310	APP	P05067	ITGA11	Q9UKX5	0.097166691
1311	APP	P05067	CHRM1	P11229	0.097265413
1312	APP	P05067	ITGAV	P06756	0.097265413
1313	APP	P05067	ADGRL1	O94910	0.097388815
1314	APP	P05067	P2RY4	P51582	0.097388815
1315	APP	P05067	CAP2	P40123	0.097388815
1316	APP	P05067	BLNK	Q8WV28	0.097907103
1317	APP	P05067	TULP3	O75386	0.098153907
1318	APP	P05067	ITK	Q08881	0.098795597
1319	APP	P05067	EFNA1	P20827	0.098869638
1320	APP	P05067	SPRED2	Q7Z698	0.098894319
1321	APP	P05067	LRP6	O75581	0.099215164
1322	APP	P05067	GNA11	P29992	0.100177699
1323	APP	P05067	EPHA8	P29322	0.100350462

1324	APP	P05067	DDR1	Q08345	0.100695987
1325	APP	P05067	RGS7BP	Q6MZT1	0.10089343
1326	APP	P05067	PIP4K2B	P78356	0.101041512
1327	APP	P05067	TLR2	O60603	0.101436399
1328	APP	P05067	CHRNA6	Q15825	0.101781924
1329	APP	P05067	ADCY3	O60266	0.102078089
1330	APP	P05067	EPHA1	P21709	0.10215213
1331	APP	P05067	SORBS1	Q9BX66	0.103040624
1332	APP	P05067	TSPAN12	O95859	0.103262747
1333	APP	P05067	PLXNA1	Q9UIW2	0.103287428
1334	APP	P05067	CSK	P41240	0.104743571
1335	APP	P05067	APC2	O95996	0.105483982
1336	APP	P05067	TGFA	P01135	0.105558024
1337	APP	P05067	MAGI1	Q96QZ7	0.105681426
1338	APP	P05067	GABRG2	P18507	0.105706106
1339	APP	P05067	LINGO1	Q96FE5	0.106051631
1340	APP	P05067	SSTR1	P30872	0.106964806
1341	APP	P05067	VIPR1	P32241	0.107112888
1342	APP	P05067	UBE2B	P63146	0.107137568
1343	APP	P05067	TIE1	P35590	0.107162249
1344	APP	P05067	CSF1R	P07333	0.107803939
1345	APP	P05067	SMURF2	Q9HAU4	0.108050743
1346	APP	P05067	SRI	P30626	0.108667753
1347	APP	P05067	MAGI3	Q5TCQ9	0.108667753
1348	APP	P05067	CD79B	P40259	0.108791155
1349	APP	P05067	GRIN1	Q05586	0.110025174
1350	APP	P05067	ARFGAP2	Q8N6H7	0.110938348
1351	APP	P05067	ITGAM	P11215	0.111308554
1352	APP	P05067	RAB8A	P61006	0.111900884
1353	APP	P05067	OR1M1	Q8NGA1	0.111950244
1354	APP	P05067	ROR2	Q01974	0.112419172
1355	APP	P05067	CX3CL1	P78423	0.112814058
1356	APP	P05067	ADRA1B	P35368	0.113505109
1357	APP	P05067	UNC13A	Q9UPW8	0.113776593
1358	APP	P05067	AVPR1B	P47901	0.113924675
1359	APP	P05067	CD274	Q9NZQ7	0.11545486
1360	APP	P05067	IL9R	Q01113	0.115553581
1361	APP	P05067	IKBKB	O14920	0.115726344
1362	APP	P05067	PLD2	O14939	0.115849746
1363	APP	P05067	PLCH2	O75038	0.116664199
1364	APP	P05067	RASGRP4	Q8TDF6	0.117009724
1365	APP	P05067	ADCY5	O95622	0.117305889
1366	APP	P05067	NTRK2	Q16620	0.117528012

1367	APP	P05067	GRM7	Q14831	0.118564589
1368	APP	P05067	OR5AR1	Q8NGP9	0.118589269
1369	APP	P05067	OR5P2	Q8WZ92	0.119749247
1370	APP	P05067	CD2	P06729	0.120711782
1371	APP	P05067	KLRK1	P26718	0.120736463
1372	APP	P05067	KLRK1	P26718	0.120736463
1373	APP	P05067	S1PR4	O95977	0.120761143
1374	APP	P05067	LPAR6	P43657	0.120810504
1375	APP	P05067	RAB18	Q9NP72	0.121106669
1376	APP	P05067	RXFP1	Q9HBX9	0.121131349
1377	APP	P05067	PLXND1	Q9Y4D7	0.121526235
1378	APP	P05067	IL31RA	Q8NI17	0.121624957
1379	APP	P05067	ADGRV1	Q8WXG9	0.1218224
1380	APP	P05067	CELSR1	Q9NYQ6	0.121896441
1381	APP	P05067	OR5H1	A6NKK0	0.122019843
1382	APP	P05067	OR5H1	A6NKK0	0.122019843
1383	APP	P05067	NPR1	P16066	0.122069204
1384	APP	P05067	CD83	Q01151	0.122784935
1385	APP	P05067	C5AR2	Q9P296	0.123698109
1386	APP	P05067	BCAM	P50895	0.123895553
1387	APP	P05067	PSD3	Q9NYI0	0.1243398
1388	APP	P05067	TACR3	P29371	0.124586603
1389	APP	P05067	RGS9	O75916	0.124660645
1390	APP	P05067	PTCH1	Q13635	0.124710005
1391	APP	P05067	ADAM9	Q13443	0.125277654
1392	APP	P05067	GABRB1	P18505	0.125401056
1393	APP	P05067	SCRIB	Q14160	0.12816526
1394	APP	P05067	PDE6A	P16499	0.128239301
1395	APP	P05067	TNFRSF19	Q9NS68	0.128560146
1396	APP	P05067	ARL6	Q9H0F7	0.128880991
1397	APP	P05067	PRKD1	Q15139	0.129127795
1398	APP	P05067	GPR45	Q9Y5Y3	0.129201836
1399	APP	P05067	APBB1IP	Q7Z5R6	0.129646083
1400	APP	P05067	AGTR2	P50052	0.129794166
1401	APP	P05067	LAG3	P18627	0.130337134
1402	APP	P05067	ITGB5	P18084	0.130929463
1403	APP	P05067	GPR183	P32249	0.132237524
1404	APP	P05067	ARL4A	P40617	0.132755812
1405	APP	P05067	MAS1	P04201	0.132977936
1406	APP	P05067	GRIN2C	Q14957	0.133002616
1407	APP	P05067	RAB3B	P20337	0.133570265
1408	APP	P05067	SH2D2A	Q9NP31	0.133965151
1409	APP	P05067	CTNNAL1	Q9UBT7	0.134211955

1410	APP	P05067	P2RX6	O15547	0.134434079
1411	APP	P05067	PTGER4	P35408	0.135001728
1412	APP	P05067	TNFSF12	O43508	0.135273212
1413	APP	P05067	FCER1A	P12319	0.135988943
1414	APP	P05067	IQGAP1	P46940	0.136285108
1415	APP	P05067	PKD2	Q13563	0.136679994
1416	APP	P05067	VAPB	O95292	0.138876549
1417	APP	P05067	CD244	Q9BZW8	0.144207513
1418	APP	P05067	KCNK2	O95069	0.144923244
1419	APP	P05067	MST1R	Q04912	0.145885779
1420	APP	P05067	LRRN3	Q9H3W5	0.14655215
1421	APP	P05067	F2RL2	O00254	0.147095118
1422	APP	P05067	OR2C1	O95371	0.147712128
1423	APP	P05067	EDNRB	P24530	0.148847426
1424	APP	P05067	PIK3C2A	O00443	0.149044869
1425	APP	P05067	GRM5	P41594	0.149587838
1426	APP	P05067	GRM1	Q13255	0.149933363
1427	APP	P05067	GRIP1	Q9Y3R0	0.150180167
1428	APP	P05067	PROKR2	Q8NFJ6	0.150501012
1429	APP	P05067	MARK2	Q7KZI7	0.151118022
1430	APP	P05067	FZD3	Q9NPG1	0.151735031
1431	APP	P05067	RALB	P11234	0.152376721
1432	APP	P05067	OR7D4	Q8NG98	0.153215855
1433	APP	P05067	PTGER3	P43115	0.153684782
1434	APP	P05067	RHOB	P62745	0.153931586
1435	APP	P05067	PTPRU	Q92729	0.154425194
1436	APP	P05067	CYTH1	Q15438	0.155140925
1437	APP	P05067	OXGR1	Q96P68	0.155683894
1438	APP	P05067	CD3E	P07766	0.155807296
1439	APP	P05067	ITGB8	P26012	0.155881337
1440	APP	P05067	GABRQ	Q9UN88	0.156399625
1441	APP	P05067	GPR50	Q13585	0.156523027
1442	APP	P05067	OR5AP2	Q8NGF4	0.15669579
1443	APP	P05067	ADGRE5	P48960	0.157189397
1444	APP	P05067	SUCNR1	Q9BXA5	0.157905129
1445	APP	P05067	MRGPRF	Q96AM1	0.158349376
1446	APP	P05067	OR5B21	A6NL26	0.159040426
1447	APP	P05067	RIT1	Q92963	0.159089787
1448	APP	P05067	ADGRL4	Q9HBW9	0.159608075
1449	APP	P05067	OR5P3	Q8WZ94	0.160225085
1450	APP	P05067	GPR75	O95800	0.161236981
1451	APP	P05067	FLT4	P35916	0.162051434
1452	APP	P05067	ADGRF5	Q8IZF2	0.162248877

1453	APP	P05067	CNTNAP1	P78357	0.162569722
1454	APP	P05067	RAC1	P63000	0.163458216
1455	APP	P05067	FURIN	P09958	0.163482896
1456	APP	P05067	P2RY13	Q9BPV8	0.165062441
1457	APP	P05067	FZD4	Q9ULV1	0.166148378
1458	APP	P05067	ARHGAP32	A7KAX9	0.166173059
1459	APP	P05067	ANXA1	P04083	0.166173059
1460	APP	P05067	CD47	Q08722	0.166691347
1461	APP	P05067	RASA2	Q15283	0.166814749
1462	APP	P05067	GABRE	P78334	0.167900686
1463	APP	P05067	GPR87	Q9BY21	0.168690459
1464	APP	P05067	PTGER1	P34995	0.169233427
1465	APP	P05067	FCGR1A	P12314	0.169480231
1466	APP	P05067	FZD10	Q9ULW2	0.170294684
1467	APP	P05067	FPR2	P25090	0.170492127
1468	APP	P05067	FPR2	P25090	0.170492127
1469	APP	P05067	RAB9A	P51151	0.170961054
1470	APP	P05067	OPRL1	P41146	0.171059776
1471	APP	P05067	PSD4	Q8NDX1	0.171207858
1472	APP	P05067	OR5J2	Q8NH18	0.171726146
1473	APP	P05067	ARHGAP33	O14559	0.172269115
1474	APP	P05067	NOX1	Q9Y5S8	0.172392517
1475	APP	P05067	RRAS	P10301	0.175057999
1476	APP	P05067	RRAS2	P62070	0.175551607
1477	APP	P05067	OR5T3	Q8NGG3	0.176143936
1478	APP	P05067	RHOC	P08134	0.176489461

Supplementary Table 3. VIPER inference of differentially activated membrane receptors between ES-derived MNs exposed to mutSOD1 or NTg astrocyte conditioned media. The reported p-values are computed from the NES scores as described in the Methods section. Since these p-values are not yet integrated in a final list, no multiple hypothesis testing correction was performed.

No.	Receptor	p-value	Activity
1	Adgrl1	4.39E-33	11.98
2	Gria2	1.42E-30	11.49
3	Gng3	5.96E-25	10.32
4	Adgrb3	3.97E-22	9.67
5	Ryk	1.56E-21	-9.53
6	Gabrg2	7.65E-20	9.12
7	Lingo1	1.19E-19	9.07
8	Gria1	4.51E-18	8.67
9	Gabrb1	1.01E-16	8.3
10	Rit2	1.57E-16	8.25
11	Diras1	6.41E-16	8.08
12	Cap2	4.09E-15	7.85
13	Ptprn	5.69E-15	7.81
14	Gria3	1.09E-14	7.73
15	Scrib	1.12E-14	-7.72
16	Tnfrsf21	4.53E-14	7.54
17	Mast1	4.75E-14	7.54
18	Rgs7bp	9.28E-14	7.45
19	Gng2	2.79E-13	7.3
20	Ube2d3	2.92E-13	-7.3
21	Dgkz	4.52E-13	7.24
22	Rab39b	6.27E-13	7.19
23	Rhod	6.55E-13	-7.19
24	Kcnip3	7.32E-13	7.17
25	Itpr3	7.55E-13	-7.17
26	Gap43	7.76E-13	7.17
27	Cx3cl1	1.41E-12	7.08
28	Ngef	2.03E-12	7.03
29	Ptprf	3.08E-12	6.97
30	Ntrk2	4.39E-12	6.92
31	Itsn1	8.28E-12	6.83
32	Lrrn3	9.27E-12	6.82
33	Gas1	1.57E-11	-6.74
34	Cd164	1.9E-11	-6.71
35	Adgra1	3.44E-11	6.63
36	Cdh2	6.6E-11	6.53
37	P2ry12	9.69E-11	6.47

38	Ncs1	1.06E-10	6.46
39	Cnksr2	1.37E-10	6.42
40	Hbegf	1.41E-10	-6.41
41	Lancl1	1.51E-10	6.4
42	Rhoj	1.91E-10	-6.37
43	Cntnap2	3E-10	6.3
44	Glrb	3.18E-10	6.29
45	Gabra2	6.27E-10	6.18
46	Rgs17	8.61E-10	6.13
47	Arhgef25	9.75E-10	6.11
48	Cnih3	1.13E-09	6.09
49	Hrh1	1.18E-09	6.08
50	Clic1	1.23E-09	-6.08
51	Gabra1	1.36E-09	6.06
52	P2ry13	1.44E-09	6.05
53	Mas1	1.44E-09	6.05
54	Tulp3	2.25E-09	-5.98
55	Magi2	2.43E-09	5.97
56	Grasp	3.28E-09	5.92
57	Arrb1	3.42E-09	5.91
58	Cnr1	5.32E-09	5.84
59	Adcy9	6.53E-09	5.8
60	Rab3a	9.38E-09	5.74
61	Adipor1	9.98E-09	-5.73
62	Adcyap1r1	1.26E-08	5.69
63	Chrna4	1.26E-08	5.69
64	Map3k12	1.63E-08	5.65
65	Adcy2	1.68E-08	5.64
66	Arf6	2.27E-08	-5.59
67	Gper1	2.63E-08	5.56
68	Slc9a3r1	3.89E-08	-5.5
69	Adam9	4.31E-08	-5.48
70	Gabrb3	4.39E-08	5.47
71	Rap1a	0.000000045	-5.47
72	Dner	5.12E-08	5.45
73	Tnfrsf19	5.18E-08	5.45
74	Tyro3	0.000000071	5.39
75	Rhoh	9.02E-08	-5.35
76	Cdk5r1	9.02E-08	5.35
77	Plxnd1	9.68E-08	5.33
78	Bcl10	0.000000106	-5.32
79	Rnd1	0.000000121	5.29
80	Rhob	0.000000146	5.26

81	Gabbr1	0.00000015	5.25
82	Rasl10a	0.000000156	5.25
83	Apbb1	0.000000175	5.22
84	Gpr26	0.000000201	5.2
85	Alk	0.000000241	5.16
86	Lancl2	0.000000269	5.14
87	Gpr162	0.000000285	5.13
88	Grm5	0.000000318	5.11
89	Rab3b	0.000000347	5.1
90	Osmr	0.000000352	-5.09
91	Rhoc	0.000000367	-5.09
92	S1pr1	0.00000038	5.08
93	Rasl10b	0.000000403	5.07
94	Grm8	0.000000405	5.07
95	Atp6ap2	0.000000424	5.06
96	Epha2	0.000000429	-5.06
97	Rab33a	0.000000507	5.02
98	Ptpru	0.000000508	5.02
99	Gpr88	0.000000545	5.01
100	Celsr3	0.00000055	5.01
101	Itgb2	0.000000579	-5
102	Rasgrf2	0.000000598	4.99
103	Cx3cr1	0.000000675	4.97
104	Grm3	0.00000068	4.97
105	Cyth3	0.00000075	-4.95
106	Chrng	0.000000784	4.94
107	Epha4	0.000000994	4.89
108	Ghr	0.00000102	-4.89
109	Itgb5	0.0000012	-4.86
110	Elmo1	0.00000122	4.85
111	Cnih2	0.00000128	4.84
112	Erbb3	0.00000148	4.81
113	Cd79b	0.00000163	-4.79
114	Rras	0.00000217	-4.74
115	Rhoq	0.00000221	-4.73
116	Adcy3	0.00000228	4.73
117	Rgs9	0.00000234	4.72
118	Trip10	0.00000282	-4.68
119	Ltb4r1	0.00000338	4.65
120	Ephb1	0.00000358	4.63
121	Gpr182	0.00000361	4.63
122	Sorbs1	0.00000389	4.62
123	Grm7	0.00000399	4.61

124	Amhr2	0.00000408	-4.61
125	Cd14	0.0000042	-4.6
126	Homer1	0.00000438	4.59
127	Rab40b	0.00000471	4.58
128	Rab31	0.00000521	-4.56
129	Epha7	0.00000523	4.56
130	Grin2a	0.00000685	4.5
131	Adgrg1	0.00000693	4.5
132	Tenm2	0.00000847	4.45
133	Cspg4	0.00000876	4.45
134	Unc5b	0.00000943	-4.43
135	Btk	0.0000108	-4.4
136	Anxa1	0.0000118	-4.38
137	Homer2	0.0000118	4.38
138	Ptger4	0.0000127	-4.36
139	Celsr2	0.0000138	4.35
140	Ltk	0.0000154	4.32
141	Rab9b	0.0000168	4.3
142	Rgs20	0.0000196	4.27
143	Vapb	0.0000204	4.26
144	Bst2	0.0000228	-4.24
145	Npy1r	0.0000229	4.23
146	Ralgps2	0.0000235	-4.23
147	Gabra4	0.0000241	4.22
148	Ephb3	0.0000267	4.2
149	Tnk2	0.0000269	4.2
150	Hcar1	0.0000271	4.2
151	Cd83	0.0000272	4.2
152	Gabrb2	0.0000281	4.19
153	Sema4c	0.0000297	-4.18
154	Adgrl3	0.0000306	4.17
155	Itgb1	0.0000309	-4.17
156	Htr6	0.0000325	4.16
157	Rab39	0.0000343	4.14
158	Lpar1	0.0000373	-4.12
159	Map4k2	0.0000374	4.12
160	Ifnar2	0.0000381	-4.12
161	Thy1	0.0000395	4.11
162	Cdc42se2	0.0000396	4.11
163	Cd274	0.0000449	-4.08
164	Lrp12	0.0000451	4.08
165	Ptprc	0.0000479	-4.07
166	Slc12a2	0.0000492	-4.06

167	Pkd2	0.0000507	-4.05
168	Rasd2	0.0000515	4.05
169	Itga6	0.0000518	-4.05
170	Ptk2	0.0000693	3.98
171	Prokr1	0.0000746	3.96
172	Cckbr	0.000075	3.96
173	Rab14	0.000077	3.95
174	Mark2	0.0000888	3.92
175	L1cam	0.0000893	3.92
176	Ednrb	0.0000982	3.9
177	Cdh13	0.0000994	3.89
178	Clec7a	0.000104	-3.88
179	Arhgap17	0.000112	-3.86
180	Iqgap1	0.000112	-3.86
181	Tenm3	0.000114	3.86
182	Rab3d	0.000115	3.86
183	Kcnh6	0.000119	3.85
184	Gpr171	0.00012	-3.85
185	Grb14	0.000145	3.8
186	Arhgap32	0.000148	3.79
187	Furin	0.000153	-3.79
188	Il12rb1	0.000162	-3.77
189	Hrh3	0.000165	3.77
190	Nisch	0.000174	3.75
191	Magi3	0.000198	3.72
192	Gpr83	0.000204	3.71
193	Nrg3	0.000205	3.71
194	Rab4a	0.000211	3.71
195	Grip1	0.000214	3.7
196	Rgs14	0.000216	3.7
197	Mras	0.000226	3.69
198	Usp8	0.000249	-3.66
199	Rala	0.000265	-3.65
200	Gpr34	0.000275	3.64
201	Tspan12	0.000277	3.64
202	Cd74	0.000305	-3.61
203	Cav1	0.000307	-3.61
204	Cd36	0.000343	-3.58
205	Plp2	0.000343	-3.58
206	Bcam	0.00035	3.58
207	Pdpn	0.000356	-3.57
208	Jag2	0.00037	3.56
209	Smo	0.00038	-3.55

210	Npr1	0.000389	-3.55
211	Oprd1	0.000416	3.53
212	Kitl	0.000426	-3.52
213	Gng13	0.000444	3.51
214	Sri	0.000445	-3.51
215	Itga8	0.000472	3.5
216	Rgs6	0.000476	3.49
217	Chrnb2	0.000485	-3.49
218	Arhgap33	0.00052	3.47
219	Il1rl1	0.000523	-3.47
220	Itk	0.000543	-3.46
221	Sstr2	0.00056	3.45
222	Alcam	0.000569	3.45
223	Tnfsf12	0.000589	-3.44
224	Plch2	0.000623	3.42
225	Gabrg1	0.00067	3.4
226	Gpr22	0.000676	3.4
227	Wwp1	0.000691	-3.39
228	Nkd1	0.000707	3.39
229	Klrd1	0.000712	-3.39
230	Vegfa	0.000744	-3.37
231	Gabrd	0.000757	3.37
232	Sptbn1	0.000776	-3.36
233	Gpr65	0.000802	-3.35
234	Gfra1	0.000895	3.32
235	Cntnap1	0.000908	3.32
236	Nos3	0.000954	3.3
237	Gng8	0.000959	-3.3
238	Ccr2	0.000971	3.3
239	Itpr1	0.00102	3.28
240	Ctnnal1	0.00106	-3.27
241	Arl4a	0.00106	3.27
242	Rgmb	0.00111	3.26
243	Gpr85	0.00117	3.25
244	Cyth4	0.00119	3.24
245	Gabrr2	0.00127	3.22
246	Gem	0.00132	-3.21
247	Itga4	0.00149	3.18
248	Ephb4	0.00154	-3.17
249	Oprk1	0.00163	3.15
250	Sla2	0.00163	3.15
251	Bdkrb2	0.00164	3.15
252	Ptger3	0.00168	3.14

253	Gabra3	0.00169	3.14
254	Grin1	0.00176	3.13
255	Adgre5	0.00191	-3.1
256	Pip5k1a	0.00197	3.09
257	Adgrl4	0.00205	3.08
258	Rab11b	0.00216	3.07
259	Csf1	0.00222	-3.06
260	Kcnk2	0.00224	3.06
261	Itgb6	0.00225	-3.06
262	Fcer1g	0.00226	-3.05
263	Ntrk3	0.00226	3.05
264	Rab3c	0.00237	3.04
265	Gpr37l1	0.00273	3
266	Caly	0.00276	2.99
267	Rab30	0.00287	2.98
268	Dlg4	0.00302	2.97
269	Flt1	0.00306	2.96
270	Adra2c	0.0032	2.95
271	Grik4	0.00329	2.94
272	Rab13	0.00334	-2.93
273	Homer3	0.00335	-2.93
274	Slc20a1	0.0035	-2.92
275	Ptk2b	0.00355	2.92
276	Ptprt	0.00375	2.9
277	Tmed1	0.00381	-2.89
278	Adgra2	0.00436	-2.85
279	Chrm3	0.00441	2.85
280	Rasa4	0.00452	-2.84
281	Mrgprf	0.00506	-2.8
282	Lyn	0.00517	-2.8
283	Treml1	0.00527	-2.79
284	Dnm2	0.00529	-2.79
285	G3bp1	0.00538	-2.78
286	Mc4r	0.00539	2.78
287	Sipa1l1	0.00562	2.77
288	Ikbkb	0.00656	-2.72
289	Arhgef28	0.00671	2.71
290	Prex1	0.00679	2.71
291	Grik1	0.00693	2.7
292	Ube2b	0.00724	2.69
293	Slc7a3	0.00801	2.65
294	Robo1	0.00844	2.63
295	Ece1	0.00923	2.6

296	Fzd5	0.00952	2.59
297	Trhr	0.00953	2.59
298	Tgfbr2	0.00986	-2.58
299	Smurf2	0.0108	-2.55
300	Cd3g	0.0114	-2.53
301	Rab17	0.0115	2.53
302	Sstr1	0.0115	2.53
303	Ephb6	0.0122	2.51
304	Sdcbp	0.0124	-2.5
305	Epor	0.0136	2.47
306	Acvr1	0.0143	-2.45
307	P2ry4	0.0143	2.45
308	Jak2	0.0144	-2.45
309	Cntfr	0.0148	2.44
310	Psenen	0.0149	2.44
311	Rasgrp1	0.0151	2.43
312	Itgax	0.0153	-2.43
313	Bmpr2	0.0155	2.42
314	Lat	0.0162	-2.4
315	Cxcr4	0.0163	-2.4
316	Gabra5	0.0163	2.4
317	Fcgr2b	0.0168	-2.39
318	Aplp2	0.0171	2.38
319	Rasgrp2	0.0175	2.38
320	Adgrl2	0.0175	2.38
321	Scarb1	0.0176	-2.37
322	Itgam	0.0178	2.37
323	Gngt2	0.0181	-2.36
324	Shc4	0.0184	2.36
325	Asic1	0.0185	2.36
326	Frs2	0.0192	2.34
327	Npr2	0.0195	2.34
328	Eng	0.0196	2.33
329	Olfr1444	0.0196	-2.33
330	Cd47	0.0205	2.32
331	Fzd3	0.0207	2.31
332	Adrb2	0.0213	-2.3
333	Ncstn	0.0215	-2.3
334	Inadl	0.0222	-2.29
335	Ppap2a	0.0223	2.29
336	Pdgfrb	0.0238	-2.26
337	Il15ra	0.024	2.26
338	Htr2c	0.0246	2.25

339	Lck	0.0248	-2.24
340	Gpr4	0.0262	2.22
341	Csf1r	0.0267	2.22
342	Sh2d2a	0.0267	2.22
343	Lpar6	0.0273	-2.21
344	Npy5r	0.0277	2.2
345	Fgfr3	0.0295	2.18
346	P2rx1	0.0296	2.18
347	Lat2	0.0305	-2.16
348	Rab2b	0.0309	2.16
349	Adcy4	0.0316	2.15
350	Gng4	0.034	-2.12
351	Ralb	0.034	2.12
352	Adora1	0.0346	2.11
353	Gpsm1	0.0346	-2.11
354	Ilk	0.0351	-2.11
355	Psen2	0.0354	2.1
356	Lgr5	0.0361	2.1
357	Arhgap10	0.0385	2.07
358	Nphs1	0.0397	-2.06
359	Ncr1	0.0416	-2.04
360	Htr2a	0.0423	2.03
361	Gng7	0.0426	2.03
362	Plxna4	0.0436	2.02
363	Fzd8	0.0445	2.01
364	Spred1	0.0446	2.01
365	Il1rap	0.0471	-1.99
366	Fyn	0.0476	1.98
367	Ddr1	0.0494	-1.96
368	Atrnl1	0.0495	1.96
369	Dtna	0.0504	1.96
370	Npsr1	0.0509	1.95
371	Lrp1	0.0533	-1.93
372	P2ry14	0.0565	1.91
373	Btla	0.0627	-1.86
374	Adcy5	0.0629	1.86
375	Hhip	0.0636	1.86
376	Rab28	0.0639	1.85
377	Ror2	0.0654	1.84
378	Mst1r	0.0662	1.84
379	Eda	0.067	1.83
380	Pmepa1	0.0672	1.83
381	Gpr12	0.0685	1.82

382	Olfr1052	0.0686	1.82
383	Cd70	0.0725	1.8
384	Ptger2	0.0725	-1.8
385	Fshr	0.0731	1.79
386	Cd4	0.0781	1.76
387	Ms4a1	0.0784	-1.76
388	Lrp6	0.0794	-1.75
389	Rras2	0.0808	-1.75
390	Jag1	0.0809	1.75
391	Zap70	0.081	-1.74
392	Rhoa	0.0815	-1.74
393	Adora2b	0.0835	1.73
394	Plek2	0.0837	-1.73
395	Prkd1	0.084	1.73
396	Itgav	0.0853	-1.72
397	Dok3	0.0853	-1.72
398	Gpr156	0.0858	1.72
399	Traf4	0.088	-1.71
400	Vapa	0.0882	-1.7
401	Cd160	0.0913	-1.69
402	Pik3c2a	0.0914	1.69
403	Chrna1	0.0917	-1.69
404	Htr1d	0.092	1.69
405	Tnf	0.0942	1.67
406	Tas1r1	0.0974	1.66
407	Adcy10	0.0977	1.66
408	Chrna5	0.0978	1.66
409	Mib1	0.0985	-1.65
410	Itga11	0.102	1.64
411	Chrnb4	0.103	1.63
412	Epha8	0.104	1.63
413	Gpr17	0.104	-1.62
414	Baiap2	0.105	1.62
415	Arl6	0.107	1.61
416	Ptgdr2	0.107	-1.61
417	Itga5	0.107	-1.61
418	Rhov	0.108	-1.61
419	Ecel1	0.11	1.6
420	Cd80	0.11	-1.6
421	Acvr2a	0.111	1.59
422	Grid2	0.111	1.59
423	Nmur1	0.111	1.59
424	Il12rb2	0.112	1.59

425	Gabrg3	0.115	1.58
426	Asah2	0.117	1.57
427	Gpr82	0.117	1.57
428	Cd2	0.119	-1.56
429	Kit	0.119	1.56
430	Gng10	0.119	1.56
431	Ccr10	0.12	-1.55
432	Nf2	0.123	1.54
433	Card11	0.124	-1.54
434	Itga1	0.124	1.54
435	Fzd4	0.132	1.51
436	Rab8a	0.137	-1.49
437	Sele	0.137	-1.49
438	Clec1b	0.138	-1.48
439	Cd38	0.139	-1.48
440	Gpr50	0.139	-1.48
441	Gpr160	0.14	1.48
442	Pdpk1	0.141	1.47
443	Acvr1b	0.142	1.47
444	Nod1	0.143	-1.46
445	Rab29	0.144	-1.46
446	Tas1r2	0.149	-1.44
447	Abca1	0.15	-1.44
448	Gfra2	0.151	-1.44
449	Olfr15	0.154	1.43
450	Nmbr	0.154	1.42
451	Nedd4	0.155	-1.42
452	P2ry10	0.155	1.42
453	Fgfr1	0.156	-1.42
454	C5ar2	0.157	1.42
455	Rxfp1	0.16	1.41
456	Htr2b	0.16	1.4
457	Gng12	0.162	-1.4
458	Il2ra	0.162	1.4
459	Sectm1a	0.163	-1.4
460	Chrm4	0.165	1.39
461	Lsp1	0.165	-1.39
462	Slc7a1	0.167	-1.38
463	Efna1	0.168	-1.38
464	Itgb7	0.168	-1.38
465	Cyth1	0.168	1.38
466	Stoml3	0.169	-1.38
467	Celsr1	0.169	1.37

468	Olfr1020	0.17	1.37
469	Adcy6	0.176	-1.35
470	Klrg1	0.176	1.35
471	Kcnip1	0.177	1.35
472	Spred2	0.178	-1.35
473	Adap2	0.178	-1.35
474	Xpr1	0.182	1.33
475	Vipr2	0.183	1.33
476	Rab27a	0.183	-1.33
477	Rit1	0.183	1.33
478	Magi1	0.185	1.32
479	Gna12	0.187	1.32
480	Tnfrsf13b	0.187	1.32
481	Cabp2	0.19	1.31
482	Fpr1	0.19	1.31
483	Ntrk1	0.191	1.31
484	Nae1	0.191	-1.31
485	Ccrl2	0.191	-1.31
486	Unc5c	0.195	1.3
487	Lifr	0.196	1.29
488	Rab5a	0.197	1.29
489	Ihh	0.198	1.29
490	Calcrl	0.202	-1.28
491	Gngt1	0.205	-1.27
492	Cd3e	0.207	-1.26
493	Tns2	0.208	-1.26
494	Rhebl1	0.208	-1.26
495	Ptch1	0.209	1.26
496	C9	0.212	1.25
497	Gpr146	0.215	-1.24
498	Cd48	0.22	1.23
499	Dcbld2	0.223	1.22
500	Rxfp2	0.224	1.22
501	Drd2	0.226	1.21
502	Mcf2l	0.23	-1.2
503	Gna13	0.23	-1.2
504	Fpr3	0.236	1.19
505	Il2rg	0.236	-1.19
506	Gria4	0.237	-1.18
507	Trpv4	0.24	-1.18
508	Tnfsf13b	0.24	1.18
509	Pld2	0.241	-1.17
510	Gpr63	0.242	-1.17

511	Lepr	0.243	1.17
512	Gnaz	0.248	1.16
513	Ngfr	0.25	1.15
514	Tacr3	0.252	1.15
515	Tacstd2	0.254	1.14
516	Park2	0.258	1.13
517	Pde6c	0.26	1.13
518	Kl	0.26	-1.13
519	Tnfrsf11a	0.261	1.12
520	Rock2	0.263	1.12
521	Chrna7	0.264	1.12
522	Sucnr1	0.264	1.12
523	Sort1	0.264	1.12
524	Itgb3	0.266	-1.11
525	Egf	0.27	1.1
526	Ccr9	0.271	-1.1
527	Prokr2	0.275	-1.09
528	Apc2	0.279	1.08
529	Hcar2	0.28	-1.08
530	Gpr68	0.281	1.08
531	Grin2b	0.284	1.07
532	Mc2r	0.284	-1.07
533	Tgfa	0.29	1.06
534	Itga2b	0.291	-1.06
535	Fgfbp1	0.293	1.05
536	Rrad	0.295	-1.05
537	Adgra3	0.295	-1.05
538	Epha3	0.295	1.05
539	Gpr19	0.297	1.04
540	Rab19	0.298	1.04
541	C3ar1	0.3	1.04
542	Rhog	0.303	-1.03
543	Sh2b2	0.304	1.03
544	Cav3	0.305	1.03
545	Gna11	0.307	1.02
546	Rasd1	0.309	1.02
547	Cmklr1	0.311	-1.01
548	Adra2a	0.311	-1.01
549	Shh	0.315	-1
550	Fpr2	0.317	1
551	Ror1	0.318	-1
552	Phlda3	0.32	0.99
553	Cnga3	0.321	-0.99

554	Olfr478	0.324	0.99
555	Rasgrp4	0.326	-0.98
556	Tlr2	0.327	0.98
557	Rhou	0.328	0.98
558	Tas1r3	0.332	0.97
559	Cd28	0.334	0.97
560	Avpr2	0.336	-0.96
561	Stx2	0.337	-0.96
562	Tnfrsf18	0.338	-0.96
563	Hcrtr2	0.338	0.96
564	Fasl	0.338	0.96
565	Sit1	0.339	-0.96
566	Gpr87	0.339	-0.96
567	P2rx7	0.34	0.96
568	F2r	0.349	-0.94
569	Fzd9	0.356	0.92
570	Adgrv1	0.356	0.92
571	Brs3	0.357	-0.92
572	Ptgfr	0.359	0.92
573	Gpr132	0.366	-0.9
574	Mill2	0.366	0.9
575	Adgrg7	0.368	-0.9
576	Smap1	0.369	-0.9
577	Ppp1r16b	0.373	0.89
578	Apbb1ip	0.373	-0.89
579	Cd247	0.374	0.89
580	Bdkrb1	0.375	0.89
581	Arl4c	0.375	0.89
582	Rab5b	0.376	0.89
583	Tgfbr1	0.378	0.88
584	Gpr149	0.384	-0.87
585	Tek	0.385	0.87
586	Il6ra	0.386	-0.87
587	Plxna1	0.388	-0.86
588	Olfr867	0.388	0.86
589	Gprc5b	0.391	-0.86
590	Gpr158	0.395	0.85
591	Grin3b	0.401	-0.84
592	Fzd2	0.401	-0.84
593	Glra1	0.402	0.84
594	Adgrf5	0.402	-0.84
595	Pigu	0.406	0.83
596	Adrb1	0.406	-0.83

597	Ptpre	0.406	0.83
598	Kcnh2	0.407	-0.83
599	Ereg	0.41	-0.82
600	Rab5c	0.41	0.82
601	Blnk	0.411	-0.82
602	Gprc6a	0.411	0.82
603	Rac1	0.414	-0.82
604	Eps15	0.415	0.82
605	Slc2a8	0.415	-0.82
606	Icosl	0.415	0.81
607	Adra1b	0.421	0.8
608	Pkd1	0.425	-0.8
609	Gja1	0.426	-0.8
610	Chrnb1	0.426	-0.8
611	Skap1	0.427	-0.79
612	Cd8b1	0.427	0.79
613	Adcy7	0.428	-0.79
614	Ptger1	0.429	0.79
615	Rab10	0.429	-0.79
616	Gpr1	0.432	-0.79
617	Ccr1	0.434	0.78
618	Rap1b	0.434	-0.78
619	Ltb4r2	0.435	0.78
620	Csk	0.435	-0.78
621	Epha1	0.438	-0.78
622	Arrb2	0.439	0.77
623	Grm1	0.44	0.77
624	P2rx3	0.441	-0.77
625	Akt1	0.441	-0.77
626	Slc4a11	0.446	-0.76
627	Socs7	0.446	0.76
628	Gpr173	0.446	0.76
629	Cngb3	0.448	0.76
630	Dgkq	0.454	0.75
631	Adrb3	0.455	0.75
632	Vipr1	0.457	0.74
633	Avpr1a	0.46	0.74
634	Htr1f	0.464	0.73
635	Flt4	0.467	0.73
636	Cap1	0.467	0.73
637	Gp1bb	0.467	0.73
638	Rab23	0.47	0.72
639	Il2rb	0.473	-0.72

640	Plxnb1	0.476	0.71
641	Lag3	0.478	0.71
642	Drd1	0.481	0.7
643	Amfr	0.482	-0.7
644	Hcrtr1	0.488	0.69
645	Tnfsf14	0.491	0.69
646	Gpbar1	0.491	0.69
647	Tdgf1	0.494	-0.68
648	Kdr	0.497	0.68
649	Adra1d	0.498	-0.68
650	Gcgr	0.506	0.66
651	Dll4	0.507	0.66
652	Rab40c	0.508	0.66
653	Tie1	0.511	-0.66
654	Gfra3	0.511	0.66
655	Insr	0.512	-0.66
656	Rab38	0.513	-0.65
657	Gpr3	0.514	0.65
658	Eda2r	0.515	-0.65
659	Fcer1a	0.52	0.64
660	Grik2	0.523	0.64
661	Ccr4	0.528	-0.63
662	Rasa2	0.533	-0.62
663	Gprc5d	0.538	-0.62
664	Oxgr1	0.546	-0.6
665	Gab2	0.548	0.6
666	Prkca	0.552	0.59
667	P2rx6	0.552	0.59
668	Pdgfra	0.552	-0.59
669	Smpd2	0.553	-0.59
670	Cd244	0.553	-0.59
671	Uts2r	0.554	-0.59
672	Fzd6	0.56	-0.58
673	Mchr1	0.563	0.58
674	Cxcr1	0.564	-0.58
675	Epha6	0.564	0.58
676	Il6st	0.564	0.58
677	Gpr27	0.572	-0.57
678	Olfr186	0.572	0.56
679	Acvrl1	0.577	-0.56
680	Olfr24	0.577	-0.56
681	Gpc3	0.577	0.56
682	Tlr1	0.582	-0.55

683	Grik3	0.584	-0.55
684	Arap3	0.587	0.54
685	Erbb2	0.59	0.54
686	Gpr21	0.594	0.53
687	Itgae	0.599	-0.53
688	Gpr35	0.602	0.52
689	Fzd10	0.607	0.51
690	Psen1	0.607	-0.51
691	Rpe65	0.609	0.51
692	Fgd2	0.609	0.51
693	Fzd1	0.611	-0.51
694	S1pr5	0.623	0.49
695	Adora2a	0.626	0.49
696	Mrgprg	0.629	0.48
697	Gabre	0.633	-0.48
698	Gabrr1	0.634	0.48
699	Bsg	0.634	-0.48
700	Gpr151	0.636	0.47
701	Adgrf1	0.638	-0.47
702	Avpr1b	0.64	-0.47
703	Adgrf4	0.64	0.47
704	Cd40	0.642	-0.47
705	Adora3	0.644	-0.46
706	Rab22a	0.644	0.46
707	Lgr6	0.645	-0.46
708	Lgr4	0.649	-0.46
709	Cd19	0.651	0.45
710	Rgr	0.651	-0.45
711	Golph3	0.654	-0.45
712	Rab43	0.659	-0.44
713	Pag1	0.664	0.43
714	Cnr2	0.667	0.43
715	Fzd7	0.669	0.43
716	Pde6a	0.671	0.43
717	Cav2	0.671	0.42
718	Tlr6	0.678	-0.42
719	S100a6	0.685	0.4
720	Plekha1	0.686	-0.4
721	Adra1a	0.693	0.4
722	Trat1	0.693	0.39
723	Myo6	0.694	-0.39
724	Psd3	0.702	0.38
725	Pard3	0.703	-0.38

726	Olfr187	0.706	-0.38
727	Tgfbr3	0.707	-0.38
728	Lax1	0.708	0.37
729	Ephb2	0.711	0.37
730	Pkn1	0.713	0.37
731	Cdc42se1	0.716	-0.36
732	Gpr37	0.722	0.36
733	Ghrhr	0.723	0.35
734	Tas2r108	0.723	-0.35
735	Spn	0.725	-0.35
736	Cacna1a	0.727	-0.35
737	Cxcr6	0.728	0.35
738	Chrm1	0.73	0.34
739	Epgn	0.732	-0.34
740	Chrna6	0.732	-0.34
741	Chrnb3	0.732	-0.34
742	Cdc42ep1	0.734	0.34
743	Chrne	0.737	0.34
744	Ccr7	0.738	-0.34
745	Fas	0.741	-0.33
746	Fcgr1	0.743	-0.33
747	Cxcr3	0.744	0.33
748	Adgre1	0.746	-0.32
749	P2ry6	0.746	-0.32
750	Galr2	0.749	-0.32
751	Olfr63	0.751	0.32
752	Galr3	0.756	0.31
753	Mc5r	0.756	0.31
754	Gpr183	0.757	0.31
755	Nkd2	0.761	0.3
756	Gng11	0.761	-0.3
757	Gpr143	0.762	0.3
758	Rab26	0.763	-0.3
759	Cd3d	0.767	-0.3
760	Crhr2	0.771	0.29
761	Gpr75	0.772	0.29
762	Cckar	0.773	-0.29
763	Itgb4	0.774	0.29
764	Olfr18	0.775	0.29
765	Cd8a	0.777	-0.28
766	Ager	0.781	-0.28
767	Cd44	0.782	0.28
768	Crk	0.786	0.27

769	Edar	0.793	-0.26
770	Il9r	0.794	-0.26
771	Rho	0.795	-0.26
772	Inpp5d	0.797	0.26
773	Chrna2	0.798	-0.26
774	Prkci	0.801	0.25
775	Ccr6	0.803	-0.25
776	Gpr152	0.803	-0.25
777	Dapp1	0.809	-0.24
778	Gpr61	0.81	0.24
779	Ctnnd1	0.811	-0.24
780	P2ry1	0.813	0.24
781	Gp1ba	0.813	-0.24
782	Tacr1	0.813	0.24
783	Gp6	0.817	-0.23
784	Glp2r	0.819	0.23
785	Slc9a1	0.823	-0.22
786	Ly6e	0.83	-0.21
787	Tnfrsf1a	0.831	0.21
788	Ackr3	0.835	0.21
789	Grin2c	0.838	-0.2
790	S1pr4	0.839	0.2
791	Casr	0.841	0.2
792	Tbxa2r	0.843	-0.2
793	Rgs19	0.844	0.2
794	Ralgps1	0.848	0.19
795	Unc13a	0.853	-0.19
796	Rab35	0.859	0.18
797	Chrna3	0.859	-0.18
798	P2rx5	0.861	0.18
799	Il7r	0.861	-0.18
800	Gprc5c	0.861	-0.18
801	Nox1	0.861	0.18
802	Gabrq	0.861	-0.17
803	Rab8b	0.861	0.17
804	Agtr1a	0.864	-0.17
805	P2rx4	0.866	0.17
806	Rab9	0.868	0.17
807	Irs4	0.871	-0.16
808	Gpr20	0.873	-0.16
809	Opn1mw	0.875	-0.16
810	S1pr3	0.875	-0.16
811	Ffar2	0.877	-0.15

812	Guca1b	0.878	-0.15
813	Itgb8	0.88	-0.15
814	Gpr45	0.883	0.15
815	Arfgap2	0.885	0.15
816	Gpr84	0.886	-0.14
817	Adgrg2	0.887	0.14
818	Itgal	0.887	0.14
819	Olfr13	0.888	-0.14
820	Tmprss6	0.891	-0.14
821	Mc3r	0.894	-0.13
822	Dll1	0.896	-0.13
823	Ms4a2	0.896	0.13
824	Adgrg3	0.907	0.12
825	F2rl1	0.909	0.11
826	F2rl2	0.91	0.11
827	Pip4k2b	0.911	0.11
828	Htr3b	0.914	0.11
829	Itga2	0.918	-0.1
830	Nfam1	0.921	-0.1
831	Pth2r	0.922	-0.1
832	Ticam2	0.924	0.1
833	Mpl	0.933	0.08
834	Adgrg6	0.934	0.08
835	Chrnd	0.934	0.08
836	Mrgpre	0.94	0.08
837	Itga3	0.94	0.07
838	Olfr1019	0.941	0.07
839	Npbwr1	0.941	0.07
840	Ncam1	0.941	-0.07
841	Agtr2	0.944	-0.07
842	Rhof	0.944	-0.07
843	Oprl1	0.945	-0.07
844	Xcr1	0.945	0.07
845	Rab18	0.95	0.06
846	Calcr	0.95	0.06
847	Il31ra	0.951	-0.06
848	Taar1	0.958	-0.05
849	Olfr480	0.964	0.05
850	Il4ra	0.967	0.04
851	Ackr4	0.967	-0.04
852	Olfr1044	0.967	0.04
853	Npffr2	0.969	-0.04
854	Adcy1	0.969	-0.04

855	Rab11a	0.969	0.04
856	Tshr	0.97	-0.04
857	Rgma	0.971	-0.04
858	Lhcgr	0.971	-0.04
859	Psd4	0.973	0.03
860	Gnrhr	0.976	-0.03
861	Cd79a	0.977	0.03
862	Rab25	0.977	0.03
863	Olfr1094	0.978	0.03
864	Tacr2	0.983	0.02
865	Unc5a	0.986	0.02
866	Aplnr	0.986	-0.02
867	Tlr7	0.987	-0.02
868	Klrk1	0.988	-0.02
869	Cysltr1	0.992	-0.01
870	Ccr5	0.994	-0.01
871	Ccr8	0.994	0.01
872	Tlr9	0.994	0.01
873	Hrh4	0.996	-0.01

Supplementary Table 4. List of upstream candidate modulators of NFKB1 transcription factor activity based on CINDy analysis. The reported p-values are computed as one-tailed test from a null model based on the empirical distribution of the number of triplets (i.e., receptor-TF-targets) inferred by the CINDy algorithm as described in the Methods section. The function empPvals from the package qvalue-2.8.0 in the R environment version 3.4.1. was used. Since these p-values are not yet integrated in a final list, no multiple hypothesis testing correction was performed.

No.	Modulator	Transcription Factor	p-value
1	Tgfbr2	Nfkb1	1.28479E-06
2	Ptprn	Nfkb1	1.34805E-06
3	Treml1	Nfkb1	2.51884E-06
4	Grik3	Nfkb1	7.26632E-06
5	Il31ra	Nfkb1	2.0507E-05
6	Itgb1	Nfkb1	3.61916E-05
7	Csf1	Nfkb1	4.08456E-05
8	Itgb5	Nfkb1	4.72293E-05
9	Apbb1ip	Nfkb1	5.66343E-05
10	P2rx4	Nfkb1	5.66343E-05
11	Nod1	Nfkb1	7.48326E-05
12	G3bp1	Nfkb1	0.000102567
13	Adgrl1	Nfkb1	0.000114402
14	Adrb1	Nfkb1	0.000114402
15	Slc7a1	Nfkb1	0.000120093
16	Cd48	Nfkb1	0.000124547
17	Mark2	Nfkb1	0.000142344
18	Kcnk2	Nfkb1	0.000168734
19	Rhoq	Nfkb1	0.000179306
20	Rasgrp1	Nfkb1	0.000245992
21	Lingo1	Nfkb1	0.000277833
22	Cyth3	Nfkb1	0.000302554
23	Rab18	Nfkb1	0.000345941
24	Anxa1	Nfkb1	0.000354476
25	Rab29	Nfkb1	0.000372186
26	Ager	Nfkb1	0.000376751
27	Osmr	Nfkb1	0.000390785
28	Kitl	Nfkb1	0.000452375
29	Gp6	Nfkb1	0.000457928
30	Ephb3	Nfkb1	0.00050489
31	Pdgfra	Nfkb1	0.000549936
32	Prkca	Nfkb1	0.000599021
33	Pdgfrb	Nfkb1	0.000668657
34	Tnfsf13b	Nfkb1	0.00067688
35	Adgra3	Nfkb1	0.000702165

36	Acvrl1	Nfkb1	0.000728398
37	Grik2	Nfkb1	0.000746433
38	Ryk	Nfkb1	0.000907847
39	Rasl10a	Nfkb1	0.001064532
40	Glrb	Nfkb1	0.001118013
41	Tnfsf12	Nfkb1	0.001145759
42	Rgs14	Nfkb1	0.001233221
43	Rap1a	Nfkb1	0.001428822
44	Mas1	Nfkb1	0.001519271
45	Gab2	Nfkb1	0.001615484
46	Olfr1444	Nfkb1	0.001635451
47	Itsn1	Nfkb1	0.001966569
48	Rhod	Nfkb1	0.002307727
49	Itgb2	Nfkb1	0.002365265
50	Akt1	Nfkb1	0.002394574
51	Cdh13	Nfkb1	0.002610238
52	Rras	Nfkb1	0.00281061
53	Cap2	Nfkb1	0.002880788
54	Cd3e	Nfkb1	0.00298938
55	Arhgap17	Nfkb1	0.003026486
56	Rab11a	Nfkb1	0.003299563
57	Itk	Nfkb1	0.003424074
58	Inpp5d	Nfkb1	0.003553323
59	Itpr3	Nfkb1	0.00359749
60	Furin	Nfkb1	0.00364221
61	C5ar2	Nfkb1	0.00373334
62	Grasp	Nfkb1	0.004070789
63	Lpar6	Nfkb1	0.004277261
64	Adap2	Nfkb1	0.004962298
65	Aplp2	Nfkb1	0.005024156
66	Rras2	Nfkb1	0.005024156
67	Adam9	Nfkb1	0.00527947
68	Rab40b	Nfkb1	0.005345319
69	Grm5	Nfkb1	0.005411997
70	Gabrb1	Nfkb1	0.005687215
71	Bcl10	Nfkb1	0.006126773
72	Rab3d	Nfkb1	0.006280791
73	Gria3	Nfkb1	0.006359261
74	Bmpr2	Nfkb1	0.00643872
75	Traf4	Nfkb1	0.00643872
76	Gabra4	Nfkb1	0.007290646
77	Gabre	Nfkb1	0.007381871
78	Pdpk1	Nfkb1	0.007381871

79	Tbxa2r	Nfkb1	0.007855597
80	Rhoc	Nfkb1	0.008154464
81	P2ry12	Nfkb1	0.00867835
82	Rab3b	Nfkb1	0.00923629
83	Rab39b	Nfkb1	0.009588331
84	Rab9	Nfkb1	0.009588331
85	Arhgap10	Nfkb1	0.00970867
86	Spn	Nfkb1	0.010078933
87	Gpr12	Nfkb1	0.010594949
88	Gem	Nfkb1	0.011137717
89	Fcgr2b	Nfkb1	0.011277753
90	Gabrr2	Nfkb1	0.011419572
91	Hbegf	Nfkb1	0.01170865
92	Hrh3	Nfkb1	0.012309234
93	Adcy2	Nfkb1	0.01310406
94	Arrb1	Nfkb1	0.013605718
95	Rhoh	Nfkb1	0.013605718
96	Sdcbp	Nfkb1	0.014126847
97	Rrad	Nfkb1	0.014485463
98	Unc5b	Nfkb1	0.01523063
99	Rgs19	Nfkb1	0.015617674
100	Trpv4	Nfkb1	0.015814921
101	Itgam	Nfkb1	0.016014696
102	Cx3cl1	Nfkb1	0.017707926
103	Gabra3	Nfkb1	0.017931985
104	Rasd2	Nfkb1	0.017931985
105	Ghr	Nfkb1	0.018388777
106	Mast1	Nfkb1	0.018388777
107	Cd79b	Nfkb1	0.018857386
108	Gabrr1	Nfkb1	0.021120957
109	Chrna6	Nfkb1	0.021388944
110	Gnaz	Nfkb1	0.021388944
111	Rgs20	Nfkb1	0.021388944
112	Sipa1l1	Nfkb1	0.021388944
113	C3ar1	Nfkb1	0.021660387
114	Fgfr1	Nfkb1	0.021935332
115	Dnm2	Nfkb1	0.022213825
116	Dok3	Nfkb1	0.02336423
117	Oprl1	Nfkb1	0.02336423
118	Sri	Nfkb1	0.023961973
119	Cd14	Nfkb1	0.025204564
120	Itpr1	Nfkb1	0.025204564
121	Fzd5	Nfkb1	0.025850253

122	Avpr1a	Nfkb1	0.027192618
123	Ifnar2	Nfkb1	0.027192618
124	Cd274	Nfkb1	0.027890208
125	Diras1	Nfkb1	0.028245812
126	Nfam1	Nfkb1	0.02897094
127	Chrna4	Nfkb1	0.029340589
128	Bdkrb2	Nfkb1	0.030094373
129	Rac1	Nfkb1	0.030094373
130	Unc5a	Nfkb1	0.030094373
131	Gria1	Nfkb1	0.03166172
132	Chrng	Nfkb1	0.032066403
133	Homer2	Nfkb1	0.032891671
134	Jag2	Nfkb1	0.033312398
135	Mras	Nfkb1	0.033312398
136	Il1rl1	Nfkb1	0.034607832
137	Rab35	Nfkb1	0.035954729
138	Rasa4	Nfkb1	0.035954729
139	P2ry4	Nfkb1	0.036415493
140	Plxna1	Nfkb1	0.036415493
141	Epha4	Nfkb1	0.037355199
142	Drd1	Nfkb1	0.03930965
143	Csf1r	Nfkb1	0.040325765
144	Adcy9	Nfkb1	0.040843855
145	Slc9a3r1	Nfkb1	0.041368753
146	Rgs17	Nfkb1	0.042985238
147	Iqgap1	Nfkb1	0.044098676
148	Rgs6	Nfkb1	0.045241655
149	Tulp3	Nfkb1	0.045241655
150	Ly6e	Nfkb1	0.045824477
151	Rab10	Nfkb1	0.046414991
152	Fgd2	Nfkb1	0.047013303
153	Ddr1	Nfkb1	0.048856103
154	Rala	Nfkb1	0.048856103
155	Tnfrsf21	Nfkb1	0.04948669
156	Ralgps2	Nfkb1	0.050125626
157	Dner	Nfkb1	0.050773024
158	Ptk2b	Nfkb1	0.050773024
159	Tnfrsf19	Nfkb1	0.052093678
160	Itgb3	Nfkb1	0.052767171
161	Il6ra	Nfkb1	0.053449604
162	Usp8	Nfkb1	0.053449604
163	Gna11	Nfkb1	0.057000248
164	Adgra2	Nfkb1	0.05848755

165	Nmur1	Nfkb1	0.05848755
166	Rock2	Nfkb1	0.060014777
167	Celsr2	Nfkb1	0.060793711
168	Unc13a	Nfkb1	0.060793711
169	Rasl10b	Nfkb1	0.061583048
170	Adcy4	Nfkb1	0.063193519
171	Rab14	Nfkb1	0.064847377
172	Rgs9	Nfkb1	0.065690958
173	Rhoa	Nfkb1	0.065690958
174	Grin2c	Nfkb1	0.066545849
175	Grik4	Nfkb1	0.067412207
176	Itga6	Nfkb1	0.068290195
177	P2ry6	Nfkb1	0.068290195
178	Olfr187	Nfkb1	0.069179976
179	Gabra2	Nfkb1	0.070995587
180	Rasgrf2	Nfkb1	0.071921756
181	Rab5a	Nfkb1	0.0728604
182	Ptprt	Nfkb1	0.073811694
183	Ror2	Nfkb1	0.074775818
184	Lancl2	Nfkb1	0.075752955
185	Dgkz	Nfkb1	0.076743289
186	Elmo1	Nfkb1	0.076743289
187	Npffr2	Nfkb1	0.076743289
188	Plek2	Nfkb1	0.077747008
189	Ube2b	Nfkb1	0.077747008
190	Homer3	Nfkb1	0.079795368
191	Tgfbr1	Nfkb1	0.080840399
192	Dlg4	Nfkb1	0.084061306
193	Rhoj	Nfkb1	0.086282158
194	Magi3	Nfkb1	0.090908203
195	Olfr1052	Nfkb1	0.090908203
196	Arhgap33	Nfkb1	0.094546144
197	Atp6ap2	Nfkb1	0.094546144
198	Epha1	Nfkb1	0.094546144
199	Sla2	Nfkb1	0.094546144
200	Grin2b	Nfkb1	0.09579212
201	Lpar1	Nfkb1	0.09579212
202	Gabrb3	Nfkb1	0.097055167
203	Mib1	Nfkb1	0.097055167
204	Adcy3	Nfkb1	0.099633472
205	Arap3	Nfkb1	0.100949241
206	Rab9b	Nfkb1	0.100949241
207	Chrnb1	Nfkb1	0.103635319

208	Rit1	Nfkb1	0.103635319
209	Gpr65	Nfkb1	0.107804838
210	Grm1	Nfkb1	0.107804838
211	Ptprc	Nfkb1	0.107804838
212	L1cam	Nfkb1	0.110681377
213	Cav1	Nfkb1	0.112149569
214	Skap1	Nfkb1	0.112149569
