# Supplementary material for: Systematic elucidation of neuron-astrocyte interaction in models of amyotrophic lateral sclerosis using multi-modal integrated bioinformatics workflow
Source: Nat Commun. 2020 Nov 4;11:5579. doi: 10.1038/s41467-020-19177-y (PMC7642391; doi:10.1038/s41467-020-19177-y)
Supplement: Supplementary file 2 — Reporting Summary [file 41467_2020_19177_MOESM2_ESM.pdf]

nature research

Corresponding author(s): Serge Przedborski and Andrea Califano

Last updated by author(s): Sep 23, 2020

Reporting Summary

Nature Research wishes to improve the reproducibility of the work that we publish. This form provides structure for consistency and transparency in reporting. For further information on Nature Research policies, see our Editorial Policies and the Editorial Policy Checklist.

Statistics

For all statistical analyses, confirm that the following items are present in the figure legend, table legend, main text, or Methods section.

n/a	Confirmed	
☐	☒	The exact sample size (n) for each experimental group/condition, given as a discrete number and unit of measurement
☐	☒	A statement on whether measurements were taken from distinct samples or whether the same sample was measured repeatedly
☐	☒	The statistical test(s) used AND whether they are one- or two-sided<br>*Only common tests should be described solely by name; describe more complex techniques in the Methods section.*
☐	☒	A description of all covariates tested
☐	☒	A description of any assumptions or corrections, such as tests of normality and adjustment for multiple comparisons
☐	☒	A full description of the statistical parameters including central tendency (e.g. means) or other basic estimates (e.g. regression coefficient) AND variation (e.g. standard deviation) or associated estimates of uncertainty (e.g. confidence intervals)
☐	☒	For null hypothesis testing, the test statistic (e.g. F, t, r) with confidence intervals, effect sizes, degrees of freedom and P value noted<br>*Give P values as exact values whenever suitable.*
☒	☐	For Bayesian analysis, information on the choice of priors and Markov chain Monte Carlo settings
☒	☐	For hierarchical and complex designs, identification of the appropriate level for tests and full reporting of outcomes
☐	☒	Estimates of effect sizes (e.g. Cohen's d, Pearson's r), indicating how they were calculated

Our web collection on statistics for biologists contains articles on many of the points above.

Software and code

Policy information about availability of computer code

Data collection	The source code for SEARCHIN algorithm and tutorial are available under the GNU GPLv3 license at the following URL: http://www.github.com/Califano-lab/SEARCHIN.
Data analysis	ES-derived MNs were measured using Metamorph Software analysis.<br>Graphpad Prism v7, SigmaPlot v12 were used for statistical analysis<br>Photoshop v19 was used for adjusting brightness and contrast to the images<br>Fiji ImageJ was used for measuring intensity for RNAscope data analysis<br>Python v3.6 was used in bioinformatics analysis.<br>Statistical package R version 3.4.1. RobustRankAggreg version 1.1 was used for final proiritizing of the the candidates.<br>Various algorithms like VIPER, ARACNe and CINDy were used for analysis

For manuscripts utilizing custom algorithms or software that are central to the research but not yet described in published literature, software must be made available to editors and reviewers. We strongly encourage code deposition in a community repository (e.g. GitHub). See the Nature Research guidelines for submitting code & software for further information.

Data

Policy information about availability of data

All manuscripts must include a data availability statement. This statement should provide the following information, where applicable:

- Accession codes, unique identifiers, or web links for publicly available datasets
- A list of figures that have associated raw data
- A description of any restrictions on data availability

The authors declare that the main data supporting the findings of this study are available within the article and its Supplementary Information files. The dataset

used in the manuscript are available at https://doi.org/10.5281/zenodo.403725971. The source data file has all the individual replicate values used in the paper.
Other databases used are PrePPI Database at https://honiglab.c2b2.columbia.edu/PrePPI/
Mouse brain expression profiles GEO: GSE10415

Field-specific reporting

Please select the one below that is the best fit for your research. If you are not sure, read the appropriate sections before making your selection.

- [x] Life sciences
- [ ] Behavioural & social sciences
- [ ] Ecological, evolutionary & environmental sciences

For a reference copy of the document with all sections, see nature.com/documents/nr-reporting-summary-flat.pdf

Life sciences study design

All studies must disclose on these points even when the disclosure is negative.

Sample size	No sample size calculation was performed for this study. Sample size was chosen based on historical data generated from individual assay . A minimum of 3 to maximum of 6-7 samples were chosen accounting for variability in biological samples. Highly variable datasets (e.g. mouse survival and behavior studies) included higher number of replicates (10-20) to get better power from results.
Data exclusions	No data were excluded from analysis.
Replication	All the replicates performed for the study are reported here. Key experiments were performed atleast 3 independent times. All replication attempts showed similar trends.
Randomization	Our study is not subject to randomization since it does not involve allocation of participants or samples into experimental groups.
Blinding	Investigator performing the data analysis was blind to the condition being tested. The samples were numbered during analysis to make sure there is no bias. Various procedures were put in place to eliminate sample identification during data analysis.

Reporting for specific materials, systems and methods

We require information from authors about some types of materials, experimental systems and methods used in many studies. Here, indicate whether each material, system or method listed is relevant to your study. If you are not sure if a list item applies to your research, read the appropriate section before selecting a response.

Materials & experimental systems

n/a	Involved in the study	
☐	☒	Antibodies
☐	☒	Eukaryotic cell lines
☒	☐	Palaeontology and archaeology
☐	☒	Animals and other organisms
☒	☐	Human research participants
☒	☐	Clinical data
☒	☐	Dual use research of concern

Methods

n/a	Involved in the study	
☒	☐	ChIP-seq
☒	☐	Flow cytometry
☒	☐	MRI-based neuroimaging

Antibodies

Antibodies used

rabbit polyclonal anti-GFP (1:1000; A11122; Molecular Probes, Eugene, OR) Manufacturer's website lists reactivity in mouse and humans and 1708 references.
rabbit polyclonal anti-GFP (1:1000; A11120, Molecular Probes, Eugene, OR) Manufacturer's website lists reactivity in mouse and humans and 580 references.
chicken polyclonal anti-MAP-2 (1:5000; ab5392; Abcam, Cambridge, England) Manufacturer's website lists reactivity in mouse and humans and 390 references.
mouse anti-SMI-32 for non-phosphorylated neurofilament heavy chain (1:500; SMI-32R; Covance, Princeton, NJ) Manufacturer's website lists reactivity in mouse and humans and 36 references.
mouse monoclonal anti-vimentin (1:500; V2258 Sigma-Aldrich, St. Louis, MO) Manufacturer's website lists reactivity in mouse and humans and 103 references
rabbit monoclonal anti-GFAP (1:500; a gift from Dr. James Goldman at Columbia University, not a commercial antibody)
rabbit polyclonal anti-SOD1 (1:10; ab13498; Abcam, Cambridge, England) Manufacturer's website lists reactivity in mouse and humans and 93 references
mouse monoclonal neutralizing anti-p75 (1:10; 05-446; clone ME20.4; Millipore Sigma, Massachussetts, USA) Manufacturer's website lists reactivity in mouse and humans and 9 references
anti-Fas antibody (1:50; AB16982; Millipore Sigma,Massachussetts, USA)
Alexa Fluor donkey-anti goat 594 (1:250; A-11058; Thermofisher Scientific, Massachussetts, USA)

	Alexa Fluor donkey anti-rabbit Alexa 488 (1:400; A-21206; Thermofisher Scientific, Massachussetts, USA)<br>Alexa Fluor goat anti-chicken 488 (1:400; A32931; Thermofisher Scientific, Massachussetts, USA)<br>Alexa Fluor donkey anti-mouse 488 (1:400; A-21202; Thermofisher Scientific, Massachussetts, USA)
Validation	rabbit polyclonal anti-GFP (1:1000; A11122; Molecular Probes, Eugene, OR) Manufacturer's website lists reactivity in mouse and humans and 1708 references.<br>rabbit polyclonal anti-GFP (1:1000; A11120, Molecular Probes, Eugene, OR) Manufacturer's website lists reactivity in mouse and humans and 580 references.<br>chicken polyclonal anti-MAP-2 (1:5000; ab5392; Abcam, Cambridge, England) Manufacturer's website lists reactivity in mouse and humans and 390 references.<br>mouse anti-SMI-32 for non-phosphorylated neurofilament heavy chain (1:500; SMI-32R; Covance, Princeton, NJ) Manufacturer's website lists reactivity in mouse and humans and 36 references.<br>mouse monoclonal anti-vimentin (1:500; V2258 Sigma-Aldrich, St. Louis, MO) Manufacturer's website lists reactivity in mouse and humans and 103 references<br>rabbit monoclonal anti-GFAP (1:500; a gift from Dr. James Goldman at Columbia University, not a commercial antibody)<br>rabbit polyclonal anti-SOD1 (1:10; ab13498; Abcam, Cambridge, England) Manufacturer's website lists reactivity in mouse and humans and 93 references<br>mouse monoclonal neutralizing anti-p75 (1:10; 05-446; clone ME20.4; Millipore Sigma, Massachussetts, USA) Manufacturer's website lists reactivity in mouse and humans and 9 references<br>anti-Fas antibody (1:50; AB16982; Millipore Sigma,Massachussetts, USA) Manufacturer's website lists reactivity in mouse and humans and 5 references<br>Alexa Fluor donkey-anti goat 594 (1:250; A-11058; Thermofisher Scientific, Massachussetts, USA) Manufacturer's website 80 references and the antibody is highly cross absorbed.<br>Alexa Fluor donkey anti-rabbit Alexa 488 (1:400; A-21206; Thermofisher Scientific, Massachussetts, USA) Manufacturer's website 307 references and the antibody is highly cross absorbed.<br>Alexa Fluor goat anti-chicken 488 (1:400; A-11039; Thermofisher Scientific, Massachussetts, USA) Manufacturer's website 165 references and the antibody is highly cross absorbed.

Eukaryotic cell lines

Policy information about cell lines

Cell line source(s)	Mouse Hb9::eGFP cell line obtained from Hynek Wichterle (Columbia University)
Authentication	Mouse Hb9::eGFP cell line were obtained from the lab that originally develped the cell line. qPCR and Immunostaining were performed to validate the mouse ES cell line.
Mycoplasma contamination	Cell lines are routinely tested for Mycoplasma contamination using qPCR method and the above mentioned cell lines are free of mycoplasma.
Commonly misidentified lines (See ICLAC register)	No commonly misidentified cell lines were used in this study

Animals and other organisms

Policy information about studies involving animals; ARRIVE guidelines recommended for reporting animal research

Laboratory animals	The mouse were housed under pathogen-free facility under standard 12 hr light/dark cycle. The temperature of the facility is constantly monitored and always maintained at 21+/-1.5°C with humidity of 50 +/-10% RH.<br>The entire mouse study was performed using B6.Cg-Tg(SOD1*G93A)1Gur/J mice (Cat# 004435; Jackson Laboratory) and non-transgenic controls B6SJLF1/J mice (Cat# 100012; Jackson Laboratory). Both males and females were used for the study. The animals were tested at P90 and P125 for morphological endpoints and monitored throughout life for behavior and survival studies.<br>DR6 null mice used for recovering DR6 null MNs were a gift from Genentech ((Stock # 2994, B6.129X.tm1Sjk/J from the Jackson Labs., MI, USA). Both males and females were used in the study. Spinal cord from E12.5 embryos from these mice were used for the study.
Wild animals	No wild animals were involved in the study
Field-collected samples	No samples were collected from the field for this study
Ethics oversight	All of the studies with human postmortem tissues were approved by Columbia IRB Committee protocol AAAA8153. Procedures related to mice handling and euthanasia were approved by Columbia University's Institutional Animal Care and Use Committee. Procedures related to in vitro experimentation with cells produced or derived from mice B6SJL were approved by Columbia IACUC protocols AAAD8107, AAAL2502 and AAAN2050

Note that full information on the approval of the study protocol must also be provided in the manuscript.
